# Impact of stereochemistry in 3D energetic materials science: a case based on peripheral editing of the 2,4,10-trioxaadamantane backbone

**DOI:** 10.1039/d5sc02800k

**Published:** 2025-07-21

**Authors:** Huan Li, Qi Zhou, Tianjiao Hou, Zhenxin Yi, Guixiang Wang, Long Zhu, Yuan Gao, Yu Zhang, Jun Luo

**Affiliations:** a School of Chemistry and Chemical Engineering, Nanjing University of Science and Technology Nanjing 210094 China y_zhang@njust.edu.cn luojun@njust.edu.cn; b College of Chemical Engineering, Nanjing Tech University Nanjing 211816 China

## Abstract

Stereoisomerism in energetic materials remains an underexplored area of research. Herein, two series of energetic stereoisomers, including four diastereomers of 2,4,10-trioxaadamantane-6,8,9-triyl trinitrate and three diastereomers of 9,9-dinitro-2,4,10-trioxaadamantane-6,8-diyl dinitrate, were synthesized based on the systematic alteration of their stereochemistry. The first cage-like energetic materials were generated that incorporate considerations of configurational isomerism. Notably, despite having the same molecular formulas and functional group positions, these stereoisomers exhibited some differences in density, stability, and detonation performance. These results suggest that rationally designed stereochemical editing can serve as an effective strategy for further development of high-performance energetic materials.

## Abstract 2

Two series of energetic stereoisomers were synthesized *via* peripheral editing strategy. The differences in density and detonation performance indicate that stereochemistry holds promise as an effective strategy for designing and synthesizing HEDMs.

## Graphical abstract

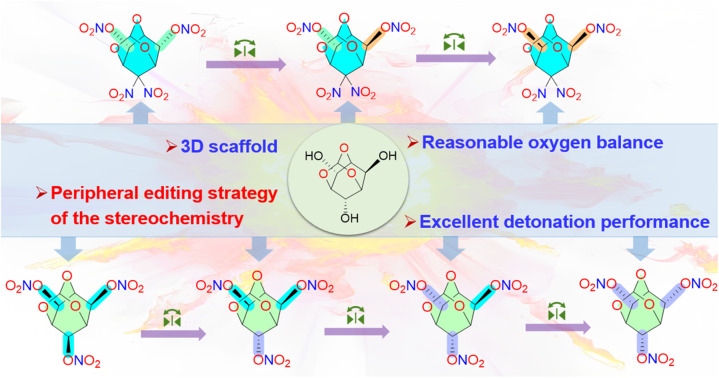

## Introduction

**Fig. 1:**
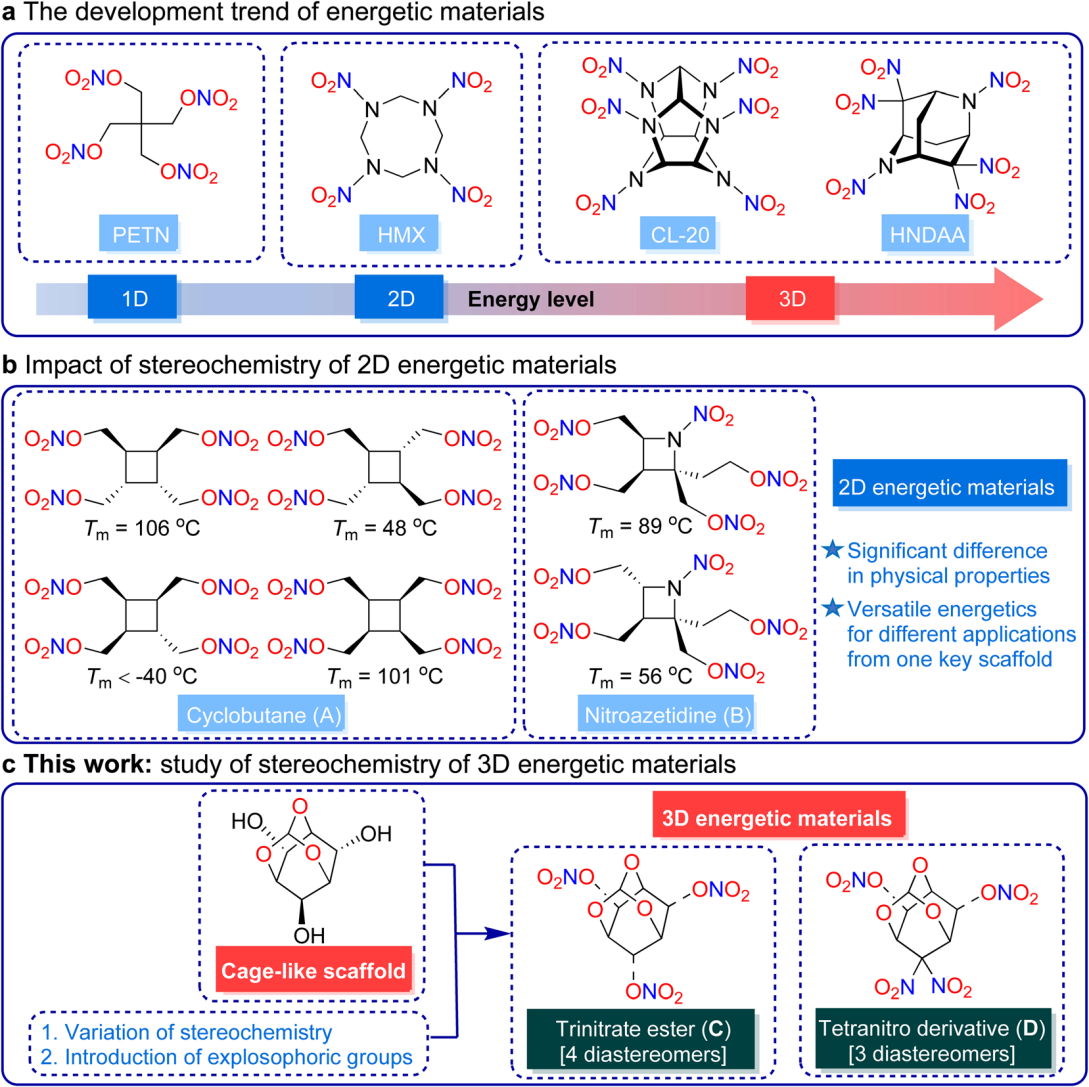
(a) The developmental trend of energetic materials. (b) Study of the stereochemistry of 2D energetic materials. (c) Study of the stereochemistry of 3D energetic materials.

The research of energetic materials has garnered considerable interest due to their growing military and civilian applications in the past few decades.^1–5^ As illustrated in Fig. 1a, the development of energetic materials is trending towards the creation of more complex molecular architectures, such as cage-like structures.^6–8^ These structures, such as CL-20 and 2,4,4,6,8,8-hexanitro-2,6-diazaadamantane (HNDAA), concentrate a significant amount of strain energy within their molecular construction, which contributes to higher energy density.^9–11^ Extensive theoretical and experimental studies have established adamantane as one of the most promising cage-like scaffolds for developing new high-energy density materials (HEDMs).^12–20^ In addition, editing the adamantane framework by replacing CH_2_ with O can effectively improve the oxygen balance of the resulting energetic compound, thereby enhancing the detonation performance.^21–24^

Density is one of the most critical parameters for energetic compounds, as the detonation velocity is directly proportional to density, and the detonation pressure is proportional to the square of the density.^25,26^ According to numerous studies of energetic compounds, there are various strategies for enhancing the density of energetic materials, but they are not completely effective. They can be summarized as: (1) introducing more explosophoric groups (NO_2_, ONO_2_, –N_3_, N–O, and NHNO_2_, *etc*.) into a molecule;^27,28^ (2) designing molecules with high symmetry and compact structures to reduce the intermolecular gaps;^29,30^ and (3) introducing additional donors or acceptors to strengthen hydrogen-bonding (HB) interactions.^31,32^ However, these strategies for promoting the density of energetic compounds rely on the continuous search for molecules with more energetic groups or complex structures, which inhibits the synthesis work and practical applications. Therefore, finding simpler and more efficient approaches to increase the density of energetic compounds remains a significant focus in the field of energetic materials.

Stereochemistry is an effective means of modifying molecular properties that has been widely applied in various fields, such as chiral catalysis,^33–35^ medicinal chemistry,^36,37^ and biochemistry.^38–40^ Energetic stereoisomers represent a class of fascinating compounds derived from distinct stereochemical configurations. However, studies examining the influence of stereochemistry on energetic materials are relatively scarce, primarily because the difference in the predicted energetic parameters of the stereoisomers is minimal.

Recent studies have challenged the previous assumption that stereochemistry does not affect the properties and detonation performance of energetic compounds. In 2019, Sabatini and Baran *et al.* pioneeringly investigated the impact of stereoisomerism on energetic materials using a series of structural and stereoisomers of cyclobutane-based nitrate esters.^41^ In 2022, Sabatini and Schindler *et al.* reported the facile synthesis of a series of azetidine-based energetic compounds with varying stereochemistries.^42^ Although energetic stereoisomers share the same molecular formula and oxygen balance, their physical properties (especially melting points) were found to be obviously different (Fig. 1b).

Depending on the stereochemistry, tunable molecules show diverse application potential as solid melt-castable explosives, as well as potential liquid propellant plasticizers, which offer new ideas and directions for the design and application of energetic materials. These energetic stereoisomers are all based on monocyclic two-dimensional (2D) backbones. To date, there have been no reports on the systematic investigation regarding the impact of configurational isomerism on the physical properties and detonation performances of energetic materials with cage-like three-dimensional (3D) scaffolds.

2,4,10-Trioxaadamantane-6,8,9-triol exhibits favorable molecular designability with its three stereocenters capable of accommodating energetic groups, and thus, it is an ideal cage-like framework for developing high-performance energetic materials and investigating the impact of configurational isomerism on 3D energetic materials. Bearing this idea in mind, we planned to synthesize two series of energetic stereoisomers bearing a 2,4,10-trioxaadamantane backbone and investigate the influence of configurational isomerism on the physical properties and detonation performances of the deduced energetic materials through peripheral editing of the stereochemistry on the 6,8,9-positions (Fig. 1c).

## Results and discussion

Although we encountered no difficulties in the preparation and handling of the compounds described in this study, it should be noted that some compounds are potential energetic materials with explosive properties and are sensitive to impact and friction. All mechanical actions on these energetic materials, such as scraping or scratching, must be strictly avoided. Appropriate standard safety precautions must be implemented for any operations involving these substances.

**Scheme 1:**
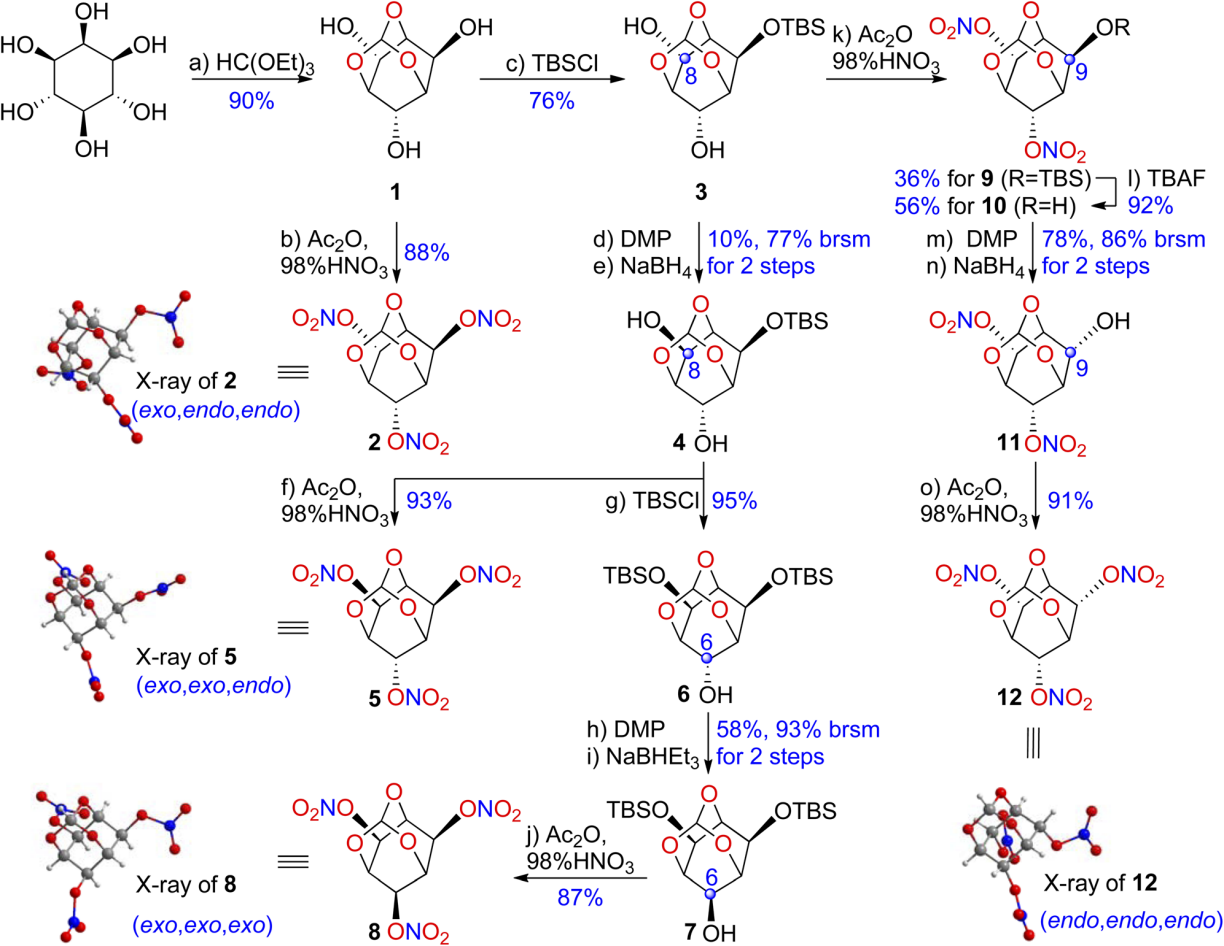
General synthetic route of all possible diastereomers of trinitrate ester.

The syntheses of all four diastereomers of 2,4,10-trioxaadamantane-6,8,9-triyl trinitrate (2, 5, 8, and 12) are shown in Scheme 1. We began the synthesis to firstly construct the cage-like framework through an acid-catalyzed transesterification. (*exo*,*endo*,*endo*)-2,4,10-Trioxaadamantane-6,8,9-triol (1) was prepared from commercially available inositol in 90% yield following a previous report.^43^ The *O*-nitration of 1 was successfully carried out in Ac_2_O/HNO_3_ and afforded the corresponding (*exo*,*endo*,*endo*)-2,4,10-trioxaadamantane-6,8,9-triyl trinitrate (2) in 88% yield, which contains two nitrato substituents axial and one equatorial in view of the cyclohexane ring. Triol 1 was amenable to selective silylation of its equatorial hydroxyl group and generated diol 3 in 76% yield.

Compound 3 was converted to 4 through mono-oxidation of a hydroxyl group, followed by reduction of the ketone. Unfortunately, even after extensive optimization, the desired product 4 could only be isolated in 10% yield (77% brsm). The poor reduction stereoselectivity might have occurred because the conforming effects of the C6 hydroxyl group contributed to a decrease in hindrance in the equatorial direction.

It is interesting that when 4 was treated with Ac_2_O/HNO_3_ at 50 °C for 4 h, the nitration of hydroxyls and nitrolysis of silyl ether simultaneously occurred and delivered (*exo*,*exo*,*endo*)-2,4,10-trioxaadamantane-6,8,9-triyl trinitrate (5) in 93% yield, which contained two equatorial nitrato substituents and one axial. TBS protection of the equatorial hydroxyl gave 6 in a yield as high as 95%, and then, a similar sequence of oxidation and reduction furnished 7 in 58% yield (93% brsm). Subsequently, 7 was treated with Ac_2_O/HNO_3_ at 50 °C for 6 h to deliver (*exo*,*exo*,*exo*)-2,4,10-trioxaadamantane-6,8,9-triyl trinitrate (8) in 87% yield.

When diol 3 was treated with Ac_2_O/HNO_3_, a mixture of 9 and 10 was isolated with yields of 36% and 56%, respectively. Further treatment of 9 with TBAF afforded 10 in 92% yield. Finally, (*endo*,*endo*,*endo*)-2,4,10-trioxaadamantane-6,8,9-triyl trinitrate (12) could be accessed in 91% yield from the known compound 10 after oxidation, reduction, and nitration.

**Scheme 2:**
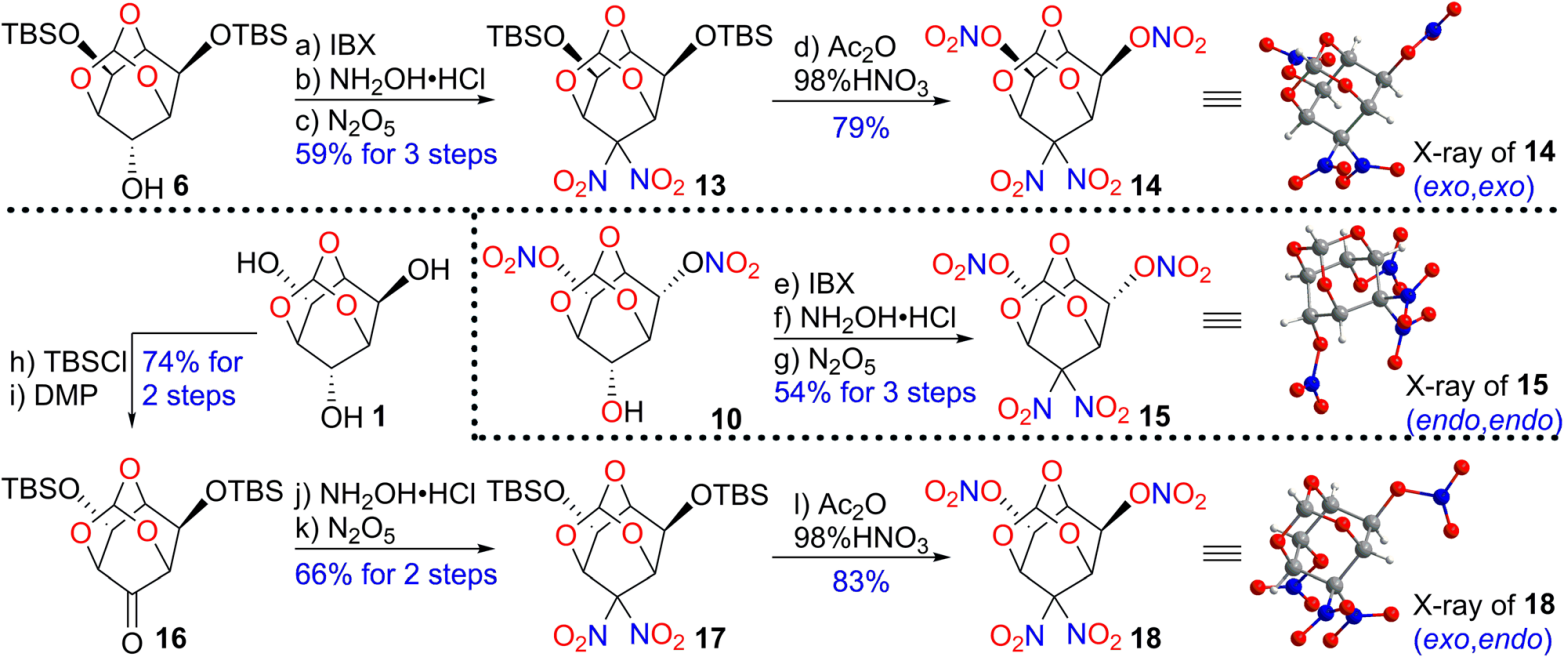
General synthetic route of all possible diastereomers of a tetranitro derivative.

Our next task was to install more nitro groups on the skeleton to enhance the density and detonation performance. Scheme 2 outlines the syntheses of three diastereomers (14, 15, and 18) of a tetranitro derivative.

Compound 6 was subjected to IBX and delivered the corresponding ketone with an almost quantitative yield. The reaction was subsequently carried out with oximation and oxidative nitration in a solution of N_2_O_5_ in CH_2_Cl_2_ to generate the *gem*-dinitro product 13 in 59% yield over 3 steps. Two silyl-ether substituents were then nitrolyzed by Ac_2_O/HNO_3_ at 50 °C for 7 h to furnish (*exo*,*exo*)-9,9-dinitro-2,4,10-trioxaadamantane-6,8-diyl dinitrate (14) in 79% yield.

Next, the (*endo*,*endo*)-stereoisomer 15 was synthesized in 54% yield over three steps using the same method, starting from the previously prepared intermediate 10. Finally, 16 could be accessed from triol 1 after TBS protection and oxidation, with a 74% yield. Ketone 16 was smoothly transformed to 17 in 66% yield through oximation and *gem*-dinitration, which further underwent nitration to give (*exo*,*endo*)-stereoisomer 18 in 83% yield.

The detonation velocity of energetic materials is directly proportional to their packing density, and thus, packing density is a crucial parameter for indicating high energy. In fact, the augmentation in the density of crystals is not only ascribed to the substitution of heavy atoms/groups but is also intimately connected with the molecular packing efficiency, which in turn is influenced by the molecular arrangement and the interlayer distances.^44^

High-performance energetic materials that feature a complex network of hydrogen bonds exhibit tighter packing due to stronger interlayer interactions, which reduces the interlayer distance.^45^ Single-crystal X-ray diffraction was performed on the configurational diastereomers of trinitrate esters 2, 5, 8, and 12, as well as the configurational diastereomers of tetranitro derivatives 14, 15, and 18, successfully confirming their structures and allowing further exploration of the relationship between structure and properties. Seven crystals of all the as-prepared energetic materials were obtained by slow recrystallization from a mixture of acetone and ethanol at room temperature. The crystallographic data are available in the SI.

For all seven crystals, the electron-withdrawing substituents on the carbon atoms play a pivotal role: there are numerous C–H⋯O H-bonds between the hydrogen atoms and the oxygen atoms in the nitro groups and on the adamantane skeleton. Molecules are symmetrically and continuously connected through hydrogen bonds, forming a three-dimensional (3D) network structure or stacking arrangement. In fact, the directional hydrogen bond interaction resulting from the conformational change of the nitrato functionalities of these stereoisomers will lead to different molecular arrangements, thus causing differences in packing coefficients.

Compound 2 crystallizes in the monoclinic space group *P*12_1_/*c*1 with four moieties per unit cell (*Z* = 4) and a crystal density of 1.816 g cm^−3^ at 296.15 K (Fig. S1). There are five types of intermolecular hydrogen bonds: C1–H1⋯O11, C1–H1⋯O12, C2–H2⋯O3, C4–H4⋯O2, and C6–H6⋯O1 (Fig. S2). Compound 5 adopted a monoclinic crystal structure within the *P*2_1_/*c* space group (*Z* = 4), and the crystal density was measured to be 1.865 g cm^−3^ at 293.15 K (Fig. S3). Compound 5 exhibits a significant number of intermolecular hydrogen bonds, involving C1–H1⋯O6, C3–H3⋯O3, C4–H4⋯O4, C5–H5⋯O10, C6–H6⋯O12, and C7–H7⋯O1 (Fig. S4).

**Fig. 2:**
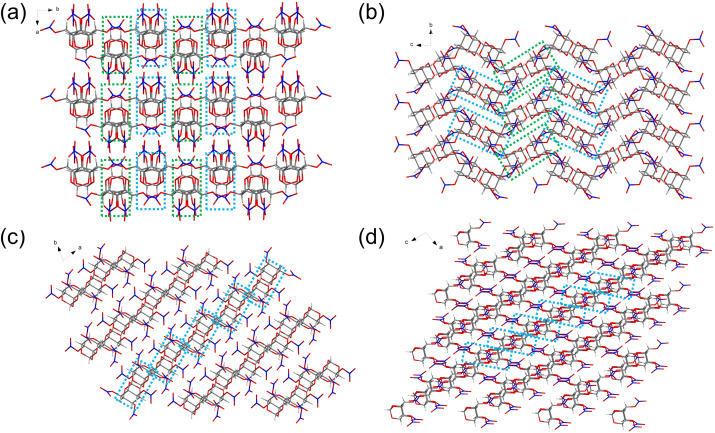
Packing diagrams of (a) 2, (b) 5, (c) 8, and (d) 12.

Compound 8 was found in the triclinic space group with *P̄*1 (*Z* = 2) symmetry, with a calculated density of 1.894 g cm^−3^ at 293 K (Fig. S5). Intermolecular hydrogen bonds of compound 8 involving C1–H1⋯O9, C2–H2⋯O4, C2–H2⋯O8, C5–H5⋯O10, and C6–H6⋯O1 were observed (Fig. S6). Compound 12 appears in the monoclinic space group with *P*2_1_/*c* (*Z* = 4) and a crystal density of 1.887 g cm^−3^ at 301 K (Fig. S7). In compound 12, the observed intermolecular hydrogen bonds include C1–H1⋯O2, C2–H2⋯O8, C3–H3⋯O2, C4–H4⋯O10, and C7–H7⋯O6 (Fig. S8). The disparities in density can be explained by comparing the crystalline packing arrangements of these stereoisomers. As shown in Fig. 2, isomers 2, 8, and 12 exhibit a layer-by-layer arrangement; however, the intralayer molecular packings of 8 and 12 are closer compared to that of 2. Additionally, isomer 5 features crossed stacking, with its interlayer hydrogen bond (2.4691 Å) shorter than that of 2 (2.5244 Å). The calculated packing coefficients of the four crystals are 70.22% (2), 72.10% (5), 73.24% (8), and 72.95% (12), which align with the order of their crystal densities.

Among the tetranitro derivatives, compound 14 exhibits monoclinic (*P*2_1_/*n*) symmetry with four moieties in the unit cell (*Z* = 4), and a crystal density of 1.899 g cm^−3^ at 294.8 K (Fig. S9). The intermolecular hydrogen bonds of 14 involve C1–H1⋯O12, C2–H2⋯O4, C5–H5⋯O13, and C6–H6⋯O10 (Fig. S10). Compound 15 occurs in the monoclinic space group with *P*2_1_/*c* symmetry, possessing a crystal density of 1.884 g cm^−3^ at 296.15 K, with four molecules in each unit cell (*Z* = 4) (Fig. S11). The structure of compound 15 is primarily governed by numerous intermolecular hydrogen bonds (C8–H8⋯O5 and C8–H8⋯N3) and intramolecular hydrogen bonds (C5–H5⋯O11 and C11–H11⋯O10) (Fig. S12).

Compound 18 forms crystals in the orthorhombic crystal system with space group *P* 21 21 21 (*Z* = 4), and the crystal density is 1.98 g cm^−3^ at 299 K (Fig. S13), which is larger than most currently used energetic compounds. Compound 18 exhibits a significant number of intermolecular hydrogen bonds, involving C3–H3⋯O16, C4–H4⋯O17, C5–H5⋯O11, C7–H7⋯O20, and C9–H9⋯O14 (Fig. S14).

**Fig. 3:**
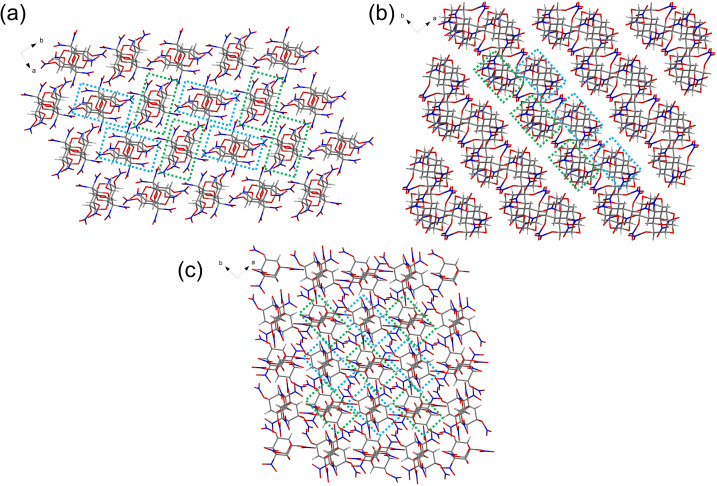
Packing diagrams of (a) 14, (b) 15, and (c) 18.

Fig. 3 clearly shows that isomer 15 exhibits a layer-by-layer arrangement, and isomers 14 and 18 are found in crossed stacking arrangements. However, isomer 18 features tighter crystal packing due to the presence of a greater number of intermolecular hydrogen bonds. Therefore, the estimated atomic packing rate in the crystals of 18 is 75.51%, which is 3% higher than that of the other two stereoisomers (72.41% for 14 and 71.74% for 15), leading to a significant increase in its crystal density (1.98 g cm^−3^).

The physical properties and detonation performance of the seven as-synthesized compounds were studied to evaluate their potential as HEDMs. As shown in Table 1, the densities of all the as-prepared compounds are higher than that of RDX.

**Table 1:** Physicochemical properties and detonation performances of the synthesized energetic materials

Compound	*ρ* a [g cm^−3^]	Δ*H*_f_^b^ [kJ mol^−1^]	*D* c [m s^−1^]	*P* d [GPa]	*T*_d_ e [°C]	IS^f^ [J]	FS^g^ [N]	OB_CO_^h^ [%]
2	1.816	−715.17	7860	27.52	165	>40	>240	+7.38
5	1.865	−722.18	8010	28.78	173	32.5	64	+7.38
8	1.894	−720.13	8109	29.89	190	>40	120	+7.38
12	1.887	−702.51	8102	29.74	173	35	140	+7.38
14	1.899	−602.79	8397	32.01	166	5	20	+13.55
15	1.884	−601.64	8348	31.64	174	30	60	+13.55
18	1.980	−611.87	8668	34.77	168	7.5	36	+13.55
TNT^23^	1.65	15.89	6958	20.14	300	15	240	−24.7
RDX^5^	1.80	177.70	8934	35.34	205	7.4	120	0
PETN^52^	1.85	−404.11	8805	34.66	166	10.2	60	+15.18

a Density determined from single-crystal X-ray diffraction analysis.

b Heat of formation.

c Calculated detonation velocity from EXPLO5_V6.05.02.

d Calculated detonation pressure from EXPLO5_V6.05.02.

e Decomposition temperature (onset).

f Impact sensitivity.

g Friction sensitivity.

h Oxygen balance assuming the formation of CO.

After determining the seven isomers, the Gaussian 09 suite of programs was used to compute some of their physicochemical properties.^46^ The molecular structures were optimized using density functional theory (DFT) based on the B3LYP functional and the 6-311++G* basis set.^47–49^ Their heats of formation were estimated using the semiempirical MO PM3 method.^50^ Using the calculated heats of formation and crystal densities, their detonation velocities and detonation pressures were calculated by the EXPLO5 (V6.05.02) program.^51^ The heats of formation, detonation velocities, and detonation pressures of TNT, RDX, and PETN were calculated using the same method that was used to evaluate the relative energy levels of these new compounds.

The adamantane skeleton was edited by replacing CH_2_ with O, and the introduction of –ONO_2_ and *gem*-dinitro groups significantly improved the oxygen balances of these novel HEDMs (Table 1). All seven compounds exhibited positive oxygen balance values based on CO (OB_CO_), indicating that their chemical energy can be fully released. These oxygen balances and high densities are in favor of enhancing the detonation performances. As listed in Table 1, the calculated detonation velocities range between 7860 and 8668 m s^−1^, while the theoretical detonation pressures span from 27.52 to 34.77 GPa. Additionally, the detonation performances of all synthesized compounds are better than that of TNT.

The most superior detonation performance was obtained for compound 18, with a detonation velocity of 8668 m s^−1^ and detonation pressure of 34.77 GPa. Overall, the detonation performances of stereoisomeric compounds are directly proportional to their single crystal densities, while tetranitro derivatives exhibit superior detonation performances as compared to trinitrate esters due to the presence of one more explosophoric group (–NO_2_).

Apart from the differences in density and detonation performance, there were also notable distinctions in the thermal stability between the as-synthesized stereoisomers. The thermal behaviors of these compounds were investigated by thermogravimetric analysis (TGA) and differential scanning calorimetry (DSC) at a heating rate of 5 °C min^−1^ (Fig. S15–S21). The onset decomposition temperatures of all the compounds ranged from 165 to 190 °C, with compound 8 exhibiting the highest value at 190 °C. The onset decomposition temperatures of compounds 8 and 15 were higher than those of their stereoisomers. Additionally, most of the trinitrate esters exhibited enhanced thermal stabilities when compared to the tetranitro derivatives. This result might be attributed to the combined effects of intermolecular hydrogen-bonding interactions and molecular symmetry.

The friction sensitivity (FS) and impact sensitivity (IS) of the as-synthesized materials were measured using standard BAM friction testing techniques (Table 1). The impact sensitivities of all the compounds, except for compound 14, were lower than that of RDX. Additionally, the friction sensitivities of compounds 2 and 12 were lower than that of RDX, while the friction sensitivities of compound 5 and all the tetranitro derivatives were higher than that of RDX. Overall, the mechanical stabilities of the trinitrate esters are greater than those of the tetranitro derivatives.

**Fig. 4:**
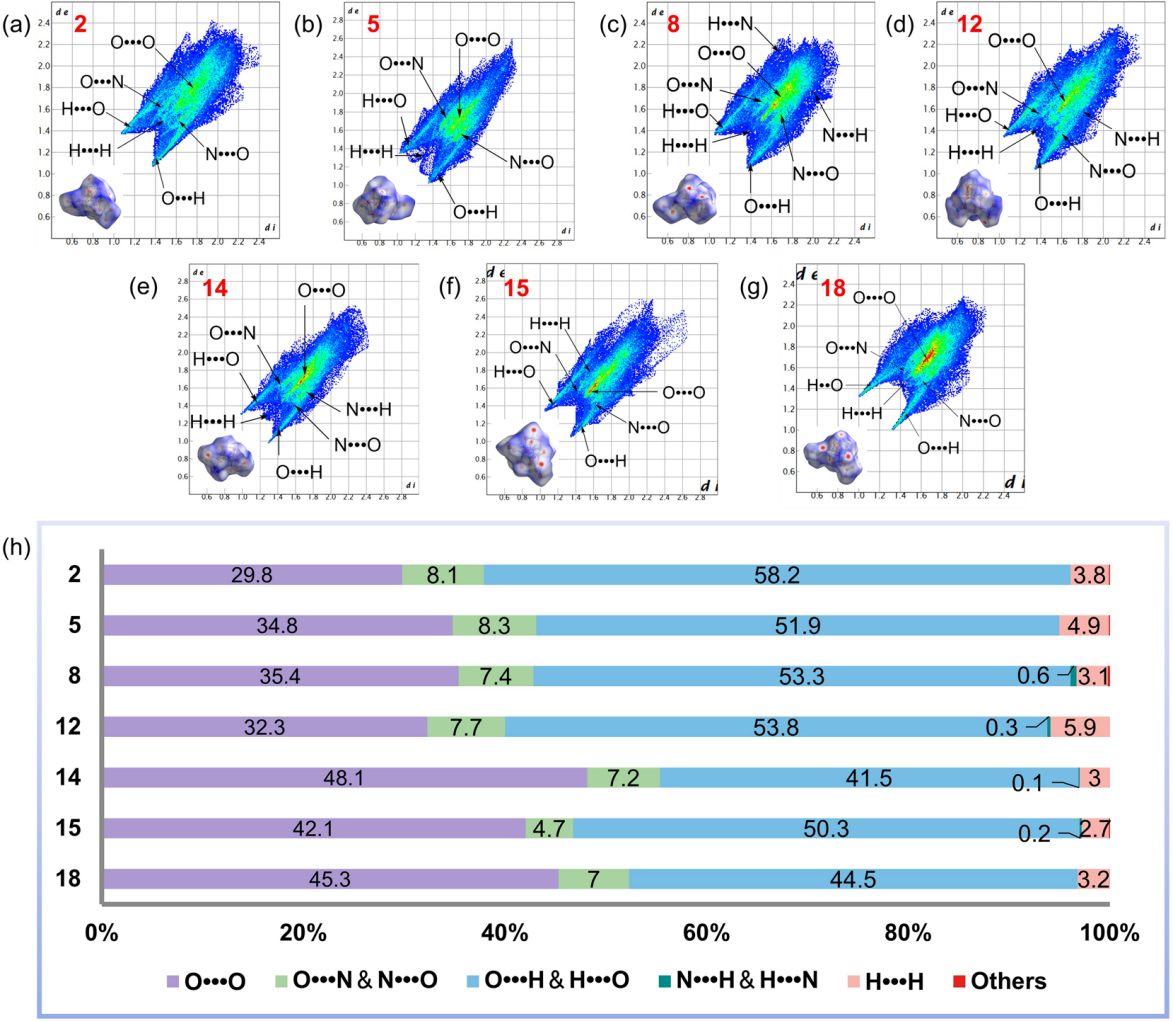
Hirshfeld surfaces and 2D fingerprint plots for (a) 2, (b) 5, (c) 8, (d) 12, (e) 14, (f) 15, and (g) 18. (h) A bar plot of the percentage contribution of a single atom contact to the Hirshfeld surface for 2, 5, 8, 12, 14, 15, and 18.

To further investigate the sensitivity of the as-synthesized stereoisomers, we examined their intermolecular interactions using 2D fingerprint plots and Hirshfeld surfaces derived from crystals (Fig. 4). In Hirshfeld surface analysis, the red and blue areas on the surface denote high and low close-contact populations, respectively. O⋯O interactions represent a highly significant form of close-contact interaction, and in most cases, a high proportion of O⋯O contacts is associated with increased sensitivity.^53^

Fig. 4h clearly demonstrates that 14 is the most sensitive compound. Among the four stereoisomers of trinitrate esters, 8 exhibits the highest proportion of O⋯O contacts at 35.4%, surpassing 2 with 29.8%, 5 with 34.8%, and 12 with 32.3%. Additionally, with 48.1% of O⋯O contacts, 14 underwent the highest proportion of these interactions, compared to 15 with 42.1% and 18 with 45.3%. Strong O⋯H interactions are typically observed in compounds with lower sensitivity.^53^ The two distinct peaks in the lower left corner of the 2D fingerprint plots of the crystals indicate a substantial presence of strong O⋯H bonding, which contributes to molecular stability and efficient crystal packing.

Compound 2 exhibits the most and strongest hydrogen bonds (58.2%), while 14 possesses the fewest and weakest H-bridges (41.5%). Based on the percentage contribution of O⋯O and O⋯N contacts and the strength and the percentage contribution of H-bridges, the order of sensitivity reduction for the as-synthesized compounds should be 14 > 18 > 15 > 5 > 8 > 12 > 2, which is consistent with the experimental results.

**Fig. 5:**
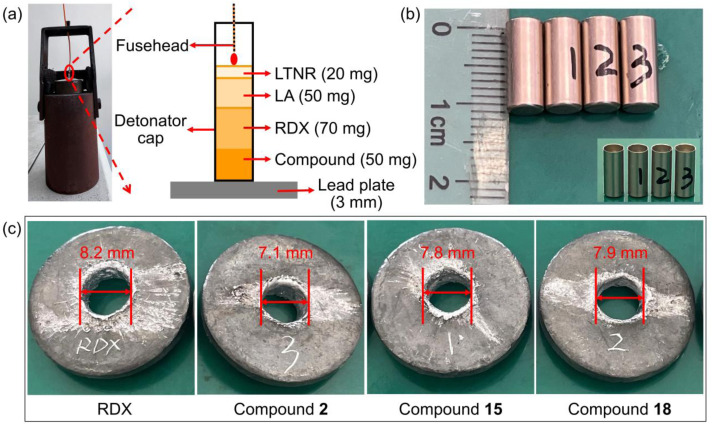
(a) Setup of the explosive test. (b) Image of detonators. (c) Perforated lead plate as a result of the detonation test.

The lead plate perforation test is a straightforward and qualitative method for assessing the performance of explosives. The setup of the explosive test is displayed in Fig. 5a, where the lead plate maintained contact with the bottom of the detonator. The diameter of the perforation formed in the lead plate after detonation is an indicator of the explosive's detonation capability. Due to limitations in route length and some yield issues, it was difficult to accumulate sufficient samples to support the lead plate experiment for all seven compounds. Therefore, considering the required time and resources, we adequately evaluated the detonation performance of these synthesized compounds by conducting lead plate perforation tests on compound 2 (with the poorest calculated detonation performance) and compound 18 (which exhibited the best calculated detonation performance). Additionally, to investigate the influence of stereochemical variations on the detonation performance of explosives, stereoisomer 15 was also chosen for lead plate testing and compared with 18.

Under the same experimental conditions, the detonation capabilities of compounds 2, 15, and 18 were tested to determine their feasibility as secondary explosives and were compared with the commonly used RDX (Fig. 5c). The lead plate was perforated by compounds 2, 15, and 18, which shows that all the compounds could be used as potential secondary explosives. Moreover, the diameters of the holes of compounds 15 and 18 are significantly larger than that of compound 2, indicating that the introduction of additional explosophoric groups on the same skeleton can significantly improve the detonation performance of energetic materials.

Interestingly, the diameter of the hole of 18 is larger than that of its diastereomer 15. The main reason for this is that compound 18 exhibits a tighter packing arrangement due to a greater number of intermolecular interactions, resulting in better detonation performance. These results demonstrate the feasibility of using stereochemistry to improve the density and detonation performance of energetic materials.

## Conclusions

Two series of three-dimensional energetic stereoisomers based on 2,4,10-trioxaadamantane skeleton, including four diastereomers (2, 5, 8, 12) of 2,4,10-trioxaadamantane-6,8,9-triyl trinitrate and three diastereomers (14, 15, 18) of 9,9-dinitro-2,4,10-trioxaadamantane-6,8-diyl dinitrate, were systematically synthesized from inositol *via* a peripheral editing strategy of the stereochemistry of hydroxyl groups. X-ray diffraction analysis revealed that these stereoisomers exhibited distinct differences in their packing arrangements, which led to a variation in density.

Specifically, among the trinitrate isomers, all-*exo* isomer 8 (1.894 g cm^−3^) and all-*endo* isomer 12 (1.887 g cm^−3^) exhibited higher crystal densities compared to (*exo*,*endo*,*endo*)-isomer 2 (1.816 g cm^−3^) and (*exo*,*exo*,*endo*)-isomer 5 (1.865 g cm^−3^) due to their superior molecular symmetry. In comparison, due to the presence of more intermolecular hydrogen bonds, (*exo*,*endo*)-isomer 18 exhibited a significantly higher crystal density (1.98 g cm^−3^) than its all-*endo* isomer 14 (1.899 g cm^−3^) and all-*exo* isomer 15 (1.884 g cm^−3^). The differences in density observed between the synthesized cage-like energetic isomers indicate that configurational isomerism holds promise as an effective strategy for designing and constructing high-energy density materials.

## Author contributions

Huan Li: synthetic experiments, investigation, writing – original draft. Qi Zhou: structural identification. Tianjiao Hou: crystallographic structural analysis. Zhenxin Yi: detonation test. Guixiang Wang: theoretical calculations. Long Zhu: investigation. Yuan Gao: supervision, writing – review and editing. Yu Zhang: data curation, supervision. Jun Luo: supervision, funding acquisition, writing – review and editing.

## Conflicts of interest

There are no conflicts to declare.

## Supplementary Material

SC-016-D5SC02800K-s001

SC-016-D5SC02800K-s002

## Data Availability

All the data needed to evaluate the study's conclusions are presented in the paper and/or in the SI. Synthesis, characterization data, X-ray crystallographic data for compounds 2, 5, 8, 12, 14, 15 and 18, calculated method for the heat of formation, and differential scanning calorimetry data. See DOI: https://doi.org/10.1039/d5sc02800k.

## References

[cit1] Zhang J., Feng Y., Bo Y., Staples R. J., Zhang J., Shreeve J. M. (2021). One step closer to an ideal insensitive energetic molecule: 3,5-diamino-6-hydroxy-2-oxide-4-nitropyrimidone and its derivatives. J. Am. Chem. Soc..

[cit2] Chen F., Wang Y., Song S., Wang K., Zhang Q. (2023). Impact of positional isomerism on melting point and stability in new energetic melt-castable materials. J. Phys. Chem. C.

[cit3] Muravyev N. V., Fershtat L., Zhang Q. (2024). Synthesis, design and development of energetic materials: Quo Vadis?. Chem. Eng. J..

[cit4] Shan Y., Huang S., Jiang T., Cao Y., Wang J., Cao Y., Zhang W. (2025). An effective strategy for balancing energy and sensitivity: design, synthesis, and properties of chimeric energetic molecules. J. Mater. Chem. A.

[cit5] Lang Q., Sun Q., Wang Q., Lin Q., Lu M. (2020). Embellishing *bis*-1,2,4-triazole with four nitroamino groups: advanced high-energy-density materials with remarkable performance and good stability. J. Mater. Chem. A.

[cit6] Zhou J., Zhang J., Wang B., Qiu L., Xu R., Sheremetev A. B. (2022). Recent synthetic efforts towards high energy density materials: how to design high-performance energetic structures?. FirePhysChem.

[cit7] Zhang J., Chen G., Gong X. (2022). Theoretical design of nitrogen-rich cages for energetic materials. Comput. Theor. Chem..

[cit8] Wu Q., Hu Q., Tan L., Zhu W. (2023). New cage super insensitive high energetic materials constructed by the Diels-Alder reaction based on nitroazoles: a DFT study. Mater. Chem. Phys..

[cit9] Bayse C. A., Jaffar M. (2020). Bonding analysis of the effect of strain on trigger bonds in organic-cage energetic materials. Theor. Chem. Acc..

[cit10] Wen L., Yu T., Lai W., Shi J., Liu M., Liu Y., Wang B. (2021). Accelerating molecular design of cage energetic materials with zero oxygen balance through large-scale database search. J. Phys. Chem. Lett..

[cit11] Kotha S., Salman M., Lal S., Cheekatla S. R., Ansari S. (2023). Design and synthesis of nitro cage heterocycles as energetic materials derived from pentacycloundecane (PCUD) systems. Asian J. Org. Chem..

[cit12] Ling Y., Zhang P., Sun L., Lai W., Luo J. (2014). Efficient synthesis of 2,2,4,4,6,6-hexanitroadamantane under mild conditions. Synthesis.

[cit13] Hou T., Ruan H., Wang G., Luo J. (2017). 2,4,4,8,8-Pentanitro-2-azaadamantane: a high-density energetic compound. Eur. J. Org Chem..

[cit14] Hou T., Zhang J., Wang C., Luo J. (2017). A facile method to construct a 2,4,9-triazaadamantane skeleton and synthesize nitramine derivatives. Org. Chem. Front..

[cit15] Zhang J., Hou T., Zhang L., Luo J. (2018). 2,4,4,6,8,8-Hexanitro-2,6-diazaadamantane: a high-energy density compound with high stability. Org. Lett..

[cit16] Zhang J., Ling Y., Wang G., Zhang L., Luo J. (2018). Synthesis of two new *gem*-fluoronitro containing tetranitroadamantanes and property comparison with their nitro and *gem*-dinitro analogues. Org. Biomol. Chem..

[cit17] Cai R., Zhou Q., Hou T., Li B., Liu Y., Li H., Gao Y., Zhu L., Luo J. (2022). Facile construction of the all-bridge-carbon-functionalized
2,4,6,8-tetraazaadamantane skeleton and conversion of its *N*-functionalities. Org. Chem. Front..

[cit18] Cai R., Li B., Hou T., Zhou Q., Liu Y., Li H., Gao Y., Zhu L., Luo J. (2023). A new polynitrogen-heterocyclic scaffold 2,4,6,8-tetraazanoradamantane skeleton: synthesis and conversion of its functionalities. Chin. J. Chem..

[cit19] Zhu L., Zhou Q., Wang G., Li H., Li B., Zhang Y., Luo J. (2025). Synthesis and characterization of a new cage-like energetic compound 3,7-dinitrato-9-nitro-9-azanoradamantane. Energy. Mater. Front..

[cit20] Zhou Q., Li H., Zhu L., Li B., Liu Y., Wang G., Zhang Y., Luo J. (2025). Construction of an all-bridge-carbon-oxidized 2-azaadamantane skeleton and synthesis of two energetic derivatives. Org. Lett..

[cit21] Liu Y., Cai R., Hou T., Wang G., Luo J. (2023). Synthesis and characterization of 6-nitro-2-oxa-6-azaadamantane-4,8-diol dinitrate. China J. Energ. Mater..

[cit22] Zhou Q., Zhu L., Cai R., Li H., Luo J. (2023). J. Synthesis of a new oxa-type cage-like energetic compound 4,4,8,8-tetranitro-2-oxaadamantane. FirePhysChem.

[cit23] Li H., Zhou Q., Zhao J., Hou T., Wang G., Zhu L., Li B., Zhang Y., Luo J. (2024). Construction of three novel oxygen-containing cage-like frameworks and synthesis of their energetic derivatives. Synlett.

[cit24] Zhou Q., Li H., Zhu L., Li B., Liu Y., Wang G., Zhang Y., Luo J. (2025). 2-Oxaadamantane-4,8,9,10-tetrayl tetranitrate: a novel oxa-type cage-like energetic compound. Energy. Mater. Front..

[cit25] Kamlet M. J., Jacobs S. J. (1968). Chemistry of Detonations. I. A simple method for calculating detonation properties of C–H–N–O explosives. J. Chem. Phys..

[cit26] Kamlet M. J., Dickinson C. (1968). Chemistry of Detonations. III. Evaluation of the simplified calculational method for Chapman-Jouguet detonation pressures on the basis of available experimental information. J. Chem. Phys..

[cit27] Yin P., Zhang J., Imler G. H., Parrish D. A., Shreeve J. M. (2017). Polynitro-functionalized dipyrazolo-1,3,5-triazinanes: energetic polycyclization toward high density and excellent molecular stability. Angew. Chem., Int. Ed..

[cit28] Gao H., Zhang Q., Shreeve J. M. (2020). Fused heterocycle-based energetic materials (2012–2019). J. Mater. Chem. A.

[cit29] Zhang W., Zhang J., Deng M., Qi X., Nie F., Zhang Q. (2017). A promising high-energy-density material. Nat. Commun..

[cit30] Zhang R., Xu Y., Yang F., Wang P., Lin Q., Huang H., Lu M. (2024). A review of ultra-high temperature heat-resistant energetic materials. Def. Technol..

[cit31] Zhang J., Zhang Q., Vo T. T., Parrish D. A., Shreeve J. M. (2015). Energetic salts with π-stacking and hydrogen-bonding interactions lead the way to future energetic materials. J. Am. Chem. Soc..

[cit32] Sun Q., Chen W., Ding N., Zhao C., Jiang Z., Li S., Pang S. (2022). Unraveling the direct effect of hydrogen bonding on density and thermostability of energetic materials through isomerism. Chem. Eng. J..

[cit33] Schmidt T., Hutskalova V., Sparr C. (2024). Atroposelective catalysis. Nat. Rev. Chem..

[cit34] Zhang Y., Palani V., Seim A. E., Wang Y., Wang K. J., Wendlandt A. E. (2022). Stereochemical editing logic powered by the epimerization of unactivated tertiary stereocenters. Science.

[cit35] Wu X., Witzig R. M., Beaud R., Fischer C., Häussinger D., Sparr C. (2021). Catalyst control over sixfold stereogenicity. Nat. Catal..

[cit36] Elder F. C., Feil E. J., Snape J., Gaze W. H., Kasprzyk-Hordern B. (2020). The role of stereochemistry of antibiotic agents in the development of antibiotic resistance in the environment. Environ. Int..

[cit37] Gandhi K., Shah U., Patel S. (2020). Drug stereochemistry: a prodigy for pharmacology and drug development. Curr. Drug Discovery Technol..

[cit38] Shakour N., Mohadeszadeh M., Iranshahi M. (2024). Biomimetic synthesis of biologically active natural products: an updated review. Mini-Rev. Med. Chem..

[cit39] Xu Y., Wang H., Yang Z., Zhou Y., Liu Y., Feng X. (2022). Stereodivergent total synthesis of rocaglaol initiated by synergistic dual-metal-catalyzed asymmetric allylation of benzofuran-3(2H)-one. Chem.

[cit40] Feng J., Pan H., Tang G. (2024). Research advances in biosynthesis of natural product drugs within the past decade. Synth. Biol. J..

[cit41] Barton L. M., Edwards J. T., Johnson E. C., Bukowski E. J., Sausa R. C., Byrd E. F., Orlicki J. A., Sabatini J. J., Baran P. S. (2019). Impact of stereo- and regiochemistry on energetic materials. J. Am. Chem. Soc..

[cit42] Rykaczewski K. A., Becker M. R., Anantpur M. J., Sausa R. C., Johnson E. C., Orlicki J. A., Bukowski E. J., Sabatini J. J., Schindler C. S. (2022). Photochemical strategies enable the synthesis of tunable azetidine-based energetic materials. J. Am. Chem. Soc..

[cit43] Duss M., Capolicchio S., Linden A., Ahmed N., Jessen H. J. (2015). Desymmetrization of *myo*-Inositol derivatives by lanthanide catalyzed phosphitylation with *C*_2_-symmetric phosphites. Bioorg. Med. Chem..

[cit44] Zhang J., Mitchell L. A., Parrish D. A., Shreeve J. M. (2015). Enforced layer-by-layer stacking of energetic salts towards high-performance insensitive energetic materials. J. Am. Chem. Soc..

[cit45] Lai Q., Pei L., Fei T., Yin P., Pang S., Shreeve J. M. (2022). Size-matched hydrogen bonded hydroxylammonium frameworks for regulation of energetic materials. Nat. Commun..

[cit46] Frisch M. J. , Trucks G. W., Schlegel H. B., Scuseria G. E., Robb M. A., Cheeseman J. R., Scalmani G., Barone V., Mennucci B., Petersson G. A., Nakatsuji H., Caricato M., Li X., Hratchian H. P., Izmaylov A. F., Bloino J., Zheng G., Sonnenberg J. L., Hada M., Ehara M., Toyota K., Fukuda R., Hasegawa J., Ishida M., Nakajima T., Honda Y., Kitao O., Nakai H., Vreven T., Montgomery J. A., Peralta J. E., Ogliaro F., Bearpark M., Heyd J. J., Brothers E., Kudin K. N., Staroverov V. N., Kobayashi R., Normand J., Raghavachari K., Rendell A., Burant J. C., Iyengar S. S., Tomasi J., Cossi M., Rega N., Millam J. M., Klene M., Knox J. E., Cross J. B., Bakken V., Adamo C., Jaramillo J., Gomperts R., Stratmann R. E., Yazyev O., Austin A. J., Cammi R., Pomelli C., Ochterski J. W., Martin R. L., Morokuma K., Zakrzewski V. G., Voth G. A., Salvador P. J., Dannenberg J., Dapprich S., Daniels A. D., Farkas Ö., Foresman J. B., Ortiz J. V., Cioslowski J. and Fox D. J., Gaussian 09, rev. Gaussian Inc, Wallingford. 2009

[cit47] Lee C. T., Yang W., Parr R. G. (1988). Development of the Colle-Salvetti Correlation-Energy formula into a functional of the electron density. Phys. Rev. B:Condens. Matter Mater. Phys..

[cit48] Becke A. D. (1992). Density-Functional Thermochemistry. II. The effect of the Perdew Wang Generalized Gradient Correlation Correction. J. Chem. Phys..

[cit49] Hariharan P. C., Pople J. A. (1973). The influence of polarization functions on molecular orbital hydrogenation energies. Theor. Chim. Acta.

[cit50] Stewart J. J. P. (1989). Optimization of parameters for semiempirical methods I. method. J. Comput. Chem..

[cit51] Suceska M. (1999). Evaluation of detonation energy from EXPLO5 computer code results. Propellants, Explos., Pyrotech..

[cit52] Lease N., Spielvogel K. D., Davis J. V., Tisdale J. T., Klamborowski L. M., Cawkwell M. J., Manner V. M. (2023). Halogenated PETN derivatives: interplay between physical and chemical factors in explosive sensitivity. Chem. Sci..

[cit53] Reichel M., Dosch D., Klapöötke T., Karaghiosoff K. (2019). Correlation between structure and energetic properties of three nitroaromatic compounds: bis(2,4-dinitrophenyl) ether, bis(2,4,6-trinitrophenyl) ether, and bis(2,4,6-trinitrophenyl) thioether. J. Am. Chem. Soc..

