# Supplementary material for: Impact of stereochemistry in 3D energetic materials science: a case based on peripheral editing of the 2,4,10-trioxaadamantane backbone
Source: Chem Sci. 2025 Jul 21;16(34):15587–96. doi: 10.1039/d5sc02800k (PMC12315248; doi:10.1039/d5sc02800k)
Supplement: SC-016-D5SC02800K-s001 [file SC-016-D5SC02800K-s001.pdf]

Supplementary Information (SI) for Chemical Science.
This journal is © The Royal Society of Chemistry 2025

Supporting Information

Impact of Stereochemistry in 3D Energetic Materials Science: A Case Based on Peripheral Editing of 2,4,10-Trioxaadamantane Backbone

Huan Li [a], Qi Zhou [a], Tianjiao Hou [b], Zhenxin Yi [a], Guixiang Wang [a], Long Zhu [a], Yuan Gao [a], Yu Zhang [a,*] and Jun Luo [a,*]

[a]. School of Chemistry and Chemical Engineering, Nanjing University of Science and Technology, Nanjing 210094, China
b. College of Chemical Engineering, Nanjing Tech University, Nanjing 211816, China
**. Corresponding authors. y_zhang@njust.edu.cn (Y. Zhang); luojun@njust.edu.cn (J. Luo)*

Table of Contents

1 General Information 1
2 Synthetic Procedures and Characterization Data 1
3 X-ray Crystallographic Analysis 13
X-ray Crystal Structure and Data of Compound **2** 13
X-ray Crystal Structure and Data of Compound **5** 20
X-ray Crystal Structure and Data of Compound **8** 25
X-ray Crystal Structure and Data of Compound **12** 29
X-ray Crystal Structure and Data of Compound **14** 34
X-ray Crystal Structure and Data of Compound **15** 39
X-ray Crystal Structure and Data of Compound **18** 44
4 Identification Spectra of Compounds 53
5 The Heat of Formation 84
6 TG-DSC curves of Energetic Compounds 85

1 General Information

Unless otherwise specified, the chemicals used in this study were obtained from commercial sources and were of analytical reagent (AR) grade. They were used without further purification. It is important to note that N_2O_5 is a strong oxidizing agent and can form explosive mixtures with organic compounds. Its decomposition produces highly toxic NO_2 gas. Therefore, N_2O_5 must be handled in closed systems under a well-ventilated hood. Eye protection and leather gloves should be worn at all times when working with N_2O_5. Petroleum ether refers to the fraction boiling in the temperature range of 60-90 °C. The progress of the reactions was monitored using thin-layer chromatography (TLC) on silica gel plates (Polygram SILG/UV 254). Column chromatography was performed using either aluminum oxide gel (spherical, neutral, 200-300 mesh) or silica gel (200-300 mesh). ^{1}H and ^{13}C nuclear magnetic resonance (NMR) spectra were obtained at frequencies of 500 MHz and 126 MHz, respectively. The solvents used for NMR measurements were $CDCl_3$, CD_3OD and C_3D_6O, with tetramethylsilane (TMS) serving as the internal standard. Fourier transform infrared (FTIR) spectra were acquired using a Nicolet FTIR IS10 Spectrometer. Thermogravimetric analysis (TGA) and differential scanning calorimetry (DSC) measurements were conducted using TGA/SDTA851e and DSC823e instruments, respectively. The heating rate employed was 5 °C/min. Elemental analysis of the newly synthesized compounds was performed using a Vario EL III CHN elemental analyzer. X-ray intensity data were collected on a Bruker D8 CMOS detector with graphite-monochromated Mo-Kα radiation (λ=0.71073 Å). The known compounds were identified by comparing their physical and spectral data with those reported in the literature. The reported yields represent the isolated yield of analytically pure material unless stated otherwise.

2 Synthetic Procedures and Characterization Data

(1*R*,3*s*,5*r*,6*R*,7*S*,8*s*,9*S*)-2,4,10-trioxaadamantane-6,8,9-triol (1)

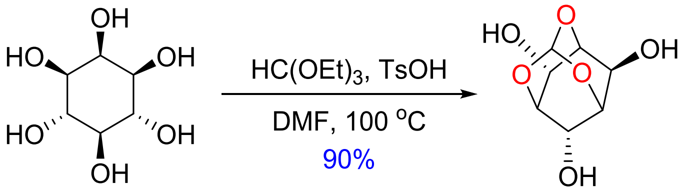

1

Triethyl orthoformate (10.0 mL, 60.1 mmol) was added to a solution of inositol (6.00 g, 33.0 mmol) in DMF (40 mL), and followed by the addition of TsOH (1.00 g, 5.0 mmol). The reaction mixture was then refluxed at 100 °C for 24 h. After completion of the reaction, the mixture was cooled to room temperature, and saturated aqueous sodium bicarbonate ($NaHCO_3$) solution (4 mL) was added. The resulting mixture was stirred for 30 minutes at room temperature and then filtered. The filtrate was subjected to evaporation using a high-vacuum rotary evaporator, resulting in the formation of a thick orange oil. To this oil, a small amount of ethyl acetate (EtOAc) was added, causing the precipitation of a yellow solid. The solid was separated by filtration, washed with EtOAc, dried, yielding compound **1** (5.64 g, 90%) as white solid.

^{1}H NMR (500 MHz, Methanol-d_4): δ 5.41 (s, 1H), 4.43 (s, 2H), 4.14 (d, J = 12.9 Hz, 2H), 4.08 (s,

2H).
^{13}C NMR (126 MHz, Methanol-d_4): δ 103.92, 75.98, 70.55, 69.05, 61.05.

(1*R*,3*s*,5*r*,6*R*,7*S*,8*s*,9*S*)-2,4,10-trioxaadamantane-6,8,9-triyl trinitrate (2)

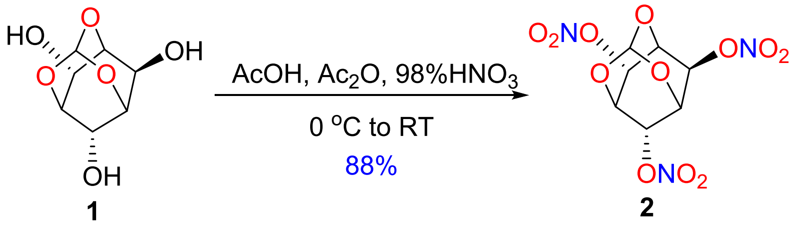

In a reaction flask, acetic acid (2 mL) and acetic anhydride (0.5 mL) were charged, and then HNO_3 (0.65 mL, 98%) was added while maintaining the reaction temperature below 5 °C (0 °C in this case). The reaction mixture was stirred for 20 minutes. Then, compound **1** (380 mg, 2.0 mmol) was added slowly in portions. After stirring for 4 h at room temperature, the reaction mixture was poured into a mixture of ice and water (10 mL). Ethyl acetate (10 mL) was added, and the mixture was extracted with EtOAc (10 mL × 3). The organic layer was washed with brine, dried with Na_2SO_4, and filtered. The solvent was then removed using a high-vacuum rotary evaporator, and the remaining residue was subjected to purification by flash column chromatography on silica gel (1:15 ethyl acetate/petroleum ether). This process yielded product **2** (493 mg, 88%) as a white solid.
^{1}H NMR (500 MHz, Acetone-d_6): δ 5.96 (q, J = 4.3 Hz, 2H), 5.82 (t, J = 5.5 Hz, 1H), 5.50 – 5.40 (m, 1H), 5.00 (d, J = 7.1 Hz, 1H), 4.88 (d, J = 6.9 Hz, 2H).
^{13}C NMR (126 MHz, Acetone-d_6): δ 103.61, 74.97, 71.59, 67.84, 66.45.
IR (thin film, υ cm^{-1}): 1652, 1636, 1271, 1156, 997, 945, 829, 744.
EA: Anal. Calcd for $C_7H_7N_3O_{12}$: C, 25.86; H, 2.17; N, 12.92. Found: C, 25.91; H, 2.18; N, 12.91.
HRMS (ESI): *m/z* calcd for $C_7H_7N_3O_{12}H^+$ $[M+H]^+$ 326.0108, found 326.0110.

(1*R*,3*s*,5*S*,6*R*,7*s*,8*S*,9*s*)-9-(*tert*-butyldimethylsiloxy)-2,4,10-trioxaadamantane-6,8-diol (3)

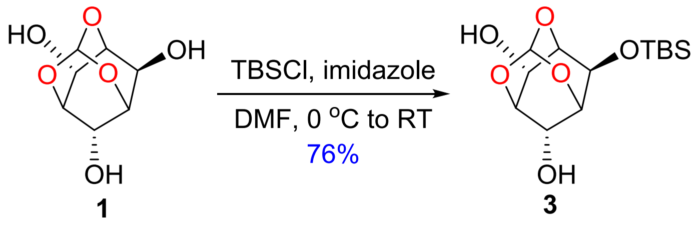

A flame dried round bottom flask equipped with a stirring bar was charged with DMF (20 mL), **1** (2.37 g, 12.5 mmol) and imidazole (1.79 g, 26.3 mmol) at 0 °C, then TBSCl (1.90 g, 12.6 mmol) was added. The resulting mixture was stirred for 2 days at room temperature and the reaction was terminated by addition of a saturated $NaHCO_3$ solution and extracted with EtOAc. The organic phases were combined and washed with water, then dried over anhydrous Na_2SO_4, filtered and concentrated under reduced pressure. The residue was then purified by flash column chromatography on silica gel (1:4 ethyl acetate/petroleum) to afford the product **3** (2.89 g, 76%) as a white solid.
^{1}H NMR (500 MHz, Methanol-d_4): δ 5.40 (d, J = 1.37 Hz, 1H), 4.41 (t, J = 3.99 Hz, 2H), 4.34 (d, J = 1.81 Hz, 1H), 4.14 (tt, J = 3.68, 1.64 Hz, 1H), 4.03 (dt, J = 4.72, 1.82 Hz, 2H), 0.96 (s, 9H), 0.16

(s, 6H).

^{13}C NMR (126 MHz, Acetone-d_6): δ 103.18, 75.75, 69.91, 69.89, 69.13, 69.11, 61.75, 18.79, -4.55.

IR (thin film, υ cm^{-1}): 3437, 2958, 2924, 2853, 1161, 1054, 1015, 977, 961, 834, 821, 774.

EA: Anal. Calcd for $C_{13}H_{24}O_6Si$: C, 51.29; H, 7.95. Found: C, 51.47; H, 7.97.

HRMS (ESI): *m/z* calcd for $C_{13}H_{24}O_6SiH^+$ $[M+H]^+$ 305.1420, found 305.1418.

(1*R*,3*S*,5*S*,6*R*,7*R*,8*R*,9*S*)-9-(*tert*-butyldimethylsiloxy)-2,4,10-trioxaadamantane-6,8-diol (4)

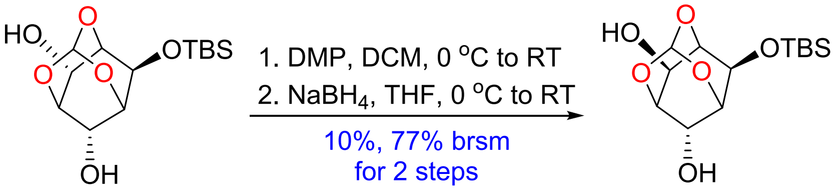

3 **4**

To a solution of **3** (3.04 g, 0.01 mol) in DCM (40.0 mL) was added Dess–Martin periodinane (4.24 g, 0.01 mol) in one portion at 0 °C. The reaction mixture was stirred for 15 min at 0 °C before it was warmed to room temperature and stirred for 8 h. The reaction mixture was quenched with saturated aqueous $NaHCO_3$ (15.0 mL) and saturated aqueous $Na_2S_2O_3$ (15.0 mL) and extracted with DCM (3 × 15 mL). The combined organic layer was washed with brine (20 mL), dried over anhydrous Na_2SO_4, filtered and concentrated under reduced pressure. The crude product ketone was used in the next step without further purification.

To a solution of crude ketone obtained above in THF (30.0 mL) at 0 °C was added $NaBH_4$ (378 mg, 0.01 mol) in portions. The reaction mixture was stirred for 12 h at room temperature and quenched with H_2O (5 mL). The mixture was extracted with EtOAc (3 × 10 mL), the combined organic layers were washed with brine (2 × 15 mL), dried over anhydrous Na_2SO_4, filtered and concentrated under reduced pressure. The residue was purified by flash column chromatography (1:3 ethyl acetate/petroleum ether) on silica gel to yield **4** (304 mg, 10% (77% brsm) over two steps) as a white solid.

^{1}H NMR (500 MHz, Acetone-d_6) δ 5.33 (s, 1H), 4.43 (dt, *J* = 5.73, 3.19 Hz, 1H), 4.08 (d, *J* = 1.68 Hz, 1H), 3.97 (dt, *J* = 3.24, 1.12 Hz, 2H), 3.93 – 3.89 (m, 1H), 3.82 (d, *J* = 1.84 Hz, 1H), 0.94 (s, 9H), 0.13 (d, *J* = 1.94 Hz, 6H).

^{13}C NMR (126 MHz, Chloroform-*d*) δ 103.56, 76.47, 73.91, 73.88, 65.37, 64.18, 64.05, 26.02, 18.53, -4.54, -4.57.

IR (thin film, υ cm^{-1}): 3489, 2954, 2929, 2885, 2855, 1461, 1388, 1247, 1165, 1151, 1118, 1081, 1065, 1000, 976, 937, 88, 848, 832, 826, 798, 777, 750, 729, 692, 665, 606.

EA: Anal. Calcd for $C_{13}H_{24}O_6Si$: C, 51.29; H, 7.95. Found: C, 51.43; H, 7.97.

(1*R*,3*r*,5*S*,6*S*,7*s*,8*R*,9*r*)-2,4,10-trioxaadamantane-6,8,9-triyl trinitrate (5)

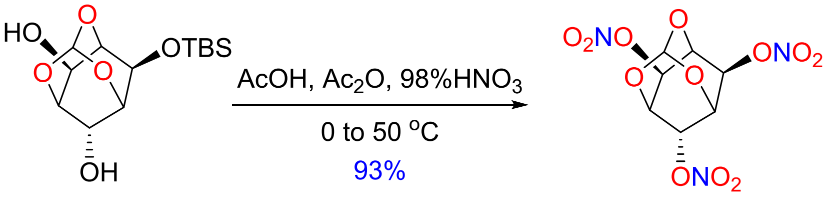

4 **5**

In a reaction flask, acetic acid (0.9 mL) and acetic anhydride (0.3 mL) were combined, and then HNO_3 (0.36 mL, 98%) was added while maintaining the reaction temperature below 5 °C (0 °C in this case). The reaction mixture was stirred for 20 minutes. Then, compound **4** (67 mg, 0.22 mol) was added slowly in portions. After stirring for 4 h at 50 °C, the reaction mixture was poured into a mixture of ice and water (10 mL). Ethyl acetate (10 mL) was added, and the mixture was extracted with EtOAc (10 mL × 3). The organic layer was washed with brine, dried with Na_2SO_4, and filtered. The solvent was then removed using a high-vacuum rotary evaporator, and the remaining residue was subjected to purification by flash column chromatography on silica gel (1:15 ethyl acetate/petroleum ether). This process yielded product **5** (67 mg, 93%) as a white solid.

^{1}H NMR (500 MHz, Acetone-*d6*) δ 6.01 (t, *J* = 4.20 Hz, 1H), 5.77 – 5.70 (m, 1H), 5.53 (q, *J* = 1.59 Hz, 2H), 4.92 (dt, *J* = 4.08, 1.86 Hz, 2H), 4.87 (t, *J* = 1.89 Hz, 1H).

^{13}C NMR (126 MHz, Acetone-d_6) δ 103.96, 73.44, 73.30, 68.67, 67.87.

IR (thin film, υ cm^{-1}): 2971, 2918, 1684, 1642, 1323, 1311, 1286, 1163, 1057, 1011, 985, 965, 928, 907, 835, 740, 701, 690, 665, 635, 612.

EA: Anal. Calcd for $C_7H_7N_3O_{12}$: C, 25.86; H, 2.17; N, 12.92. Found: C, 25.95; H, 2.18; N, 12.89.

(1*s*,3*r*,5*R*,6*s*,7*S*,8*S*,9*R*)-8,9-bis(*tert*-butyldimethylsiloxy)-2,4,10-trioxaadamantan-6-ol (6)

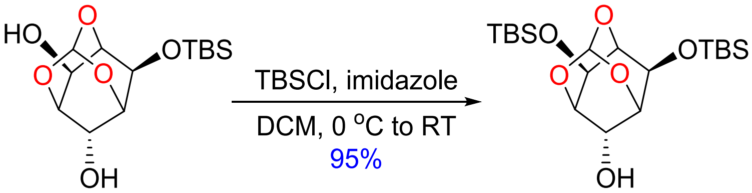

4 **6**

A flame dried round bottom flask equipped with a stirring bar was charged with CH_2Cl_2 (20 mL), **4** (548 mg, 1.8 mmol) and imidazole (184 mg, 2.7 mmol) at 0 °C, then TBSCl (407 mg, 2.7 mmol) was added. The resulting mixture was stirred for 2 days at room temperature and the reaction was terminated by addition of a saturated $NaHCO_3$ solution and extracted with EtOAc. The organic phases were combined and washed with water, then dried over anhydrous Na_2SO_4, filtered and concentrated under reduced pressure. The residue was then purified by flash column chromatography on silica gel (1:8 ethyl acetate/petroleum) to afford the products **6** (715 mg, 95%) as a white solid.

^{1}H NMR (500 MHz, Chloroform-*d*) δ 5.51 (d, *J* = 1.28 Hz, 1H), 4.54 (q, *J* = 4.03 Hz, 1H), 4.02 (dt, *J* = 4.01, 1.92 Hz, 2H), 3.94 (t, *J* = 1.62 Hz, 2H), 3.91 (p, *J* = 1.90 Hz, 1H), 2.49 (d, *J* = 4.89 Hz, 1H), 0.93 (s, 18H), 0.13 (d, *J* = 2.84 Hz, 12H).

^{13}C NMR (126 MHz, Chloroform-*d*) δ 103.24, 76.44, 74.19, 65.61, 64.29, 25.94, 18.47, -4.65, -4.73.

IR (thin film, υ cm^{-1}): 3459, 2951, 2929, 2900, 2856, 1386, 1251, 1169, 1162, 1078, 1034, 997, 962, 938, 882, 859, 832, 814, 776, 745, 689, 667, 556.

EA: Anal. Calcd for $C_{19}H_{38}O_6Si_2$: C, 54.51; H, 9.15. Found: C, 54.62; H, 9.17.

HRMS (ESI): *m/z* calcd for $C_{19}H_{38}O_6Si_2Na^+$ $[M+Na]^+$ 441.2105, found 441.2101.

(1*s*,3*r*,5*R*,6*r*,7*S*,8*S*,9*R*)-8,9-bis(*tert*-butyldimethylsiloxy)-2,4,10-trioxaadamantan-6-ol (7)

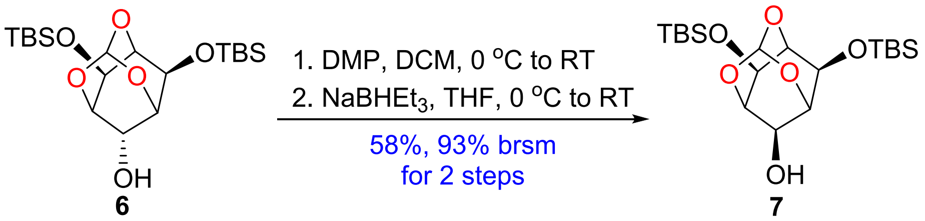

To a solution of **6** (326 mg, 0.78 mmol) in DCM (20.0 mL) was added Dess–Martin periodinane (992 mg, 2.34 mmol) in one portion at 0 °C. The reaction mixture was stirred for 15 min at 0 °C before it was warmed to room temperature and stirred for 8 h. The reaction mixture was quenched with saturated aqueous $NaHCO_3$ (5.0 mL) and saturated aqueous $Na_2S_2O_3$ (5.0 mL) and extracted with DCM (3 × 10 mL). The combined organic layer was washed with brine (10 mL), dried over anhydrous Na_2SO_4, filtered and concentrated under reduced pressure. The crude product ketone was used in the next step without further purification.

To a solution of crude ketone obtained above in THF (15.0 mL) at 0 °C was added $NaBHEt_3$ (1.56 mL, 1.0 M. in THF, 1.56 mmol) in portions. The reaction mixture was stirred for 12 h at room temperature and quenched with H_2O (5 mL). The mixture was extracted with EtOAc (3 × 10 mL), the combined organic layers were washed with brine (2 × 15 mL), dried over anhydrous Na_2SO_4, filtered and concentrated under reduced pressure. The residue was purified by flash column chromatography (1:7 ethyl acetate/petroleum ether) on silica gel to yield **7** (189 mg, 58% (93% brsm) over two steps) as a white solid.

^{1}H NMR (500 MHz, Chloroform-*d*) δ 5.52 (s, 1H), 3.96 (s, 2H), 3.87 (s, 1H), 3.54 (d, *J* = 25.81 Hz, 4H), 0.91 (s, 18H), 0.11 (s, 12H).

^{13}C NMR (126 MHz, Chloroform-*d*) δ 104.28, 76.06, 75.79, 66.10, 65.66, 25.89, 18.45, -4.58, -4.61.

IR (thin film, υ cm^{-1}): 3446, 2951, 2928, 2900, 2856, 1383, 1250, 1166, 1100, 1077, 1005, 979, 964, 905, 890, 833, 811, 775, 665, 569.

EA: Anal. Calcd for $C_{19}H_{38}O_6Si_2$: C, 54.51; H, 9.15. Found: C, 54.68; H, 9.18.

(1*s*,5*s*,6*s*,7*s*,8*s*,9*s*)-2,4,10-trioxaadamantane-6,8,9-triyl trinitrate (8)

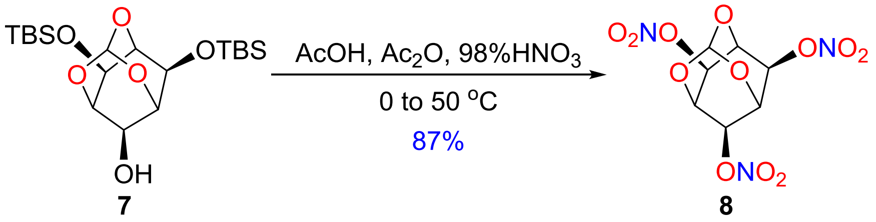

In a reaction flask, acetic acid (0.9 mL) and acetic anhydride (0.3 mL) were combined, and then HNO_3 (0.36 mL, 98%) was added while maintaining the reaction temperature below 5 °C (0 °C in this case). The reaction mixture was stirred for 20 minutes. Then, compound **7** (92 mg, 0.22 mol) was added slowly in portions. After stirring for 6 h at 50 °C, the reaction mixture was poured into a mixture of ice and water (10 mL). Ethyl acetate (10 mL) was added, and the mixture was extracted with EtOAc (10 mL × 3). The organic layer was washed with brine, dried with Na_2SO_4, and filtered. The solvent was then removed using a high-vacuum rotary evaporator, and the remaining residue was subjected to purification by flash column chromatography on silica gel (1:15 ethyl acetate/petroleum ether). This process yielded product **8** (61 mg, 87%) as a white solid.

^{1}H NMR (500 MHz, Acetone-d_6) δ 5.75 (p, J = 1.27 Hz, 3H), 5.67 (d, J = 1.21 Hz, 1H), 4.88 (q, J = 1.27 Hz, 3H).
^{13}C NMR (126 MHz, Acetone-d_6) δ 104.53, 73.46, 69.11.
IR (thin film, υ cm^{-1}): 2974, 2918, 1689, 1641, 1292, 1273, 1162, 993, 981, 895, 854, 840, 829, 800, 758, 747, 731, 675, 638, 629, 615.
EA: Anal. Calcd for $C_7H_7N_3O_{12}$: C, 25.86; H, 2.17; N, 12.92. Found: C, 25.92; H, 2.17; N, 12.90.

Compounds 9 and 10

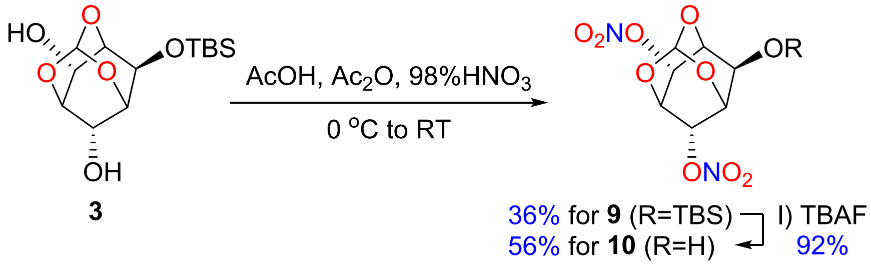

In a reaction flask, acetic acid (4.6 mL) and acetic anhydride (1.25 mL) were combined, and then HNO_3 (1.6 mL, 98%) was added while maintaining the reaction temperature below 5 °C (0 °C in this case). The reaction mixture was stirred for 20 minutes. Then, compound **3** (1.52 g, 5.0 mmol) was added slowly in portions. After stirring for 4 h at room temperature, the reaction mixture was poured into a mixture of ice and water (200 mL). Ethyl acetate (EtOAc, 10 mL) was added, and the mixture was extracted with EtOAc (10 mL × 3). The organic layer was washed with brine (10 mL), dried with Na_2SO_4, and filtered. The solvent was removed under vacuum, and the resulting residue was purified by flash column chromatography on silica gel (1:30 ethyl acetate/petroleum). This process yielded product **10** (0.71 g, 56%) as a white solid, along with the formation of product **9** in yield of 36%. Furthermore, compound **9** could be further reacted with 1N TBAF to yield product **10** with a yield of 92%.

(1*R*,3*s*,5*S*,6*R*,7*s*,8*S*,9*s*)-9-(*tert*-butyldimethylsiloxy)-2,4,10-trioxaadamantane-6,8-diyl dinitrate (9)

^{1}H NMR (500 MHz, Chloroform-d): δ 5.72 – 5.64 (m, 2H), 5.64 – 5.57 (m, 1H), 4.69 (d, J = 7.2 Hz, 1H), 4.30 – 4.20 (m, 2H), 3.98 – 3.90 (m, 1H), 0.92 (dt, J = 6.4, 3.4 Hz, 9H), 0.13 (dt, J = 6.2, 3.3 Hz, 6H).
^{13}C NMR (126 MHz, Chloroform-d): δ 103.04, 74.64, 70.73, 65.56, 61.18, 25.78, 18.33, -4.72.
IR (thin film, υ cm^{-1}): 2933, 2856, 1664, 1280, 1161, 1133, 995, 962, 826, 782.
EA: Anal. Calcd for $C_{13}H_{22}N_2O_{10}Si$: C, 39.59; H, 5.62; N, 7.10. Found: C, 39.65; H, 5.63; N, 7.09.
HRMS (ESI): m/z calcd for $C_{13}H_{22}N_2O_{10}SiNa^+$ $[M+Na]^+$ 417.0941, found 417.0953.

(1*R*,3*r*,5*S*,6*R*,7*r*,8*S*,9*r*)-9-hydroxy-2,4,10-trioxaadamantane-6,8-diyl dinitrate (10)

^{1}H NMR (500 MHz, Methanol-d_4): δ 5.76 (t, J = 3.75 Hz, 2H), 5.63 (d, J = 1.41 Hz, 1H), 4.81 – 4.76 (m, 1H), 4.35 (dt, J = 4.48, 2.11 Hz, 2H), 3.87 – 3.79 (m, 1H).
^{13}C NMR (126 MHz, Methanol-d_4): δ 104.43, 75.97, 71.59, 66.72, 61.29.
IR (thin film, υ cm^{-1}): 3527, 1663, 1635, 1274, 1153, 1005, 988, 951, 935, 833.
EA: Anal. Calcd for $C_7H_8N_2O_{10}$: C, 30.01; H, 2.88; N, 10.00. Found: C, 30.08; H, 2.89; N, 9.98.
HRMS (ESI): m/z calcd for $C_7H_8N_2O_{10}H^+$ $[M+H]^+$ 281.0257, found 281.0250.

(1*R*,3*r*,5*S*,6*R*,7*r*,8*S*,9*s*)-9-hydroxy-2,4,10-trioxaadamantane-6,8-diyl dinitrate (11)

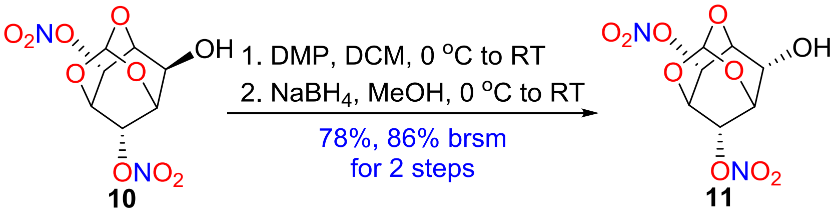

To a solution of **10** (280 mg, 1.0 mmol) in DCM (10.0 mL) was added Dess–Martin periodinane (1.27 g, 3.0 mmol) in one portion at 0 °C. The reaction mixture was stirred for 15 min at 0 °C before it was warmed to room temperature and stirred for 8 h. The reaction mixture was quenched with saturated aqueous $NaHCO_3$ (5.0 mL) and saturated aqueous $Na_2S_2O_3$ (5.0 mL) and extracted with DCM (3 × 10 mL). The combined organic layer was washed with brine (10 mL), dried over anhydrous Na_2SO_4, filtered and concentrated under reduced pressure. The crude product ketone was used in the next step without further purification.

To a solution of crude ketone obtained above in MeOH (10.0 mL) at 0 °C was added $NaBH_4$ (57 mg, 1.5 mmol) in portions. The reaction mixture was stirred for 12 h at room temperature and quenched with H_2O (5 mL). The mixture was extracted with EtOAc (3 × 10 mL), the combined organic layers were washed with brine (2 × 15 mL), dried over anhydrous Na_2SO_4, filtered and concentrated under reduced pressure. The residue was purified by flash column chromatography (1:5 ethyl acetate/petroleum ether) on silica gel to yield **11** (218 mg, 78% (86% brsm) over two steps) as a white solid.

^{1}H NMR (500 MHz, Acetone-*d*$_6$) δ 5.75 – 5.71 (m, 3H), 4.83 (tt, *J* = 3.49, 1.93 Hz, 1H), 4.62 (td, *J* = 3.70, 1.84 Hz, 2H), 4.57 – 4.53 (m, 1H).

^{13}C NMR (126 MHz, Acetone-*d*$_6$) δ 103.33, 74.52, 69.17, 69.15, 66.72, 65.06, 64.95.

IR (thin film, υ cm^{-1}): 3524, 3249, 3005, 2903, 1645, 1271, 1155, 996, 974, 944, 831, 766, 745, 726, 683, 623, 594, 575.

EA: Anal. Calcd for $C_7H_8N_2O_{10}$: C, 30.01; H, 2.88; N, 10.00. Found: C, 30.05; H, 2.88; N, 9.97.

(1*r*,5*r*,6*r*,7*r*,8*r*,9*r*)-2,4,10-trioxaadamantane-6,8,9-triyl trinitrate (12)

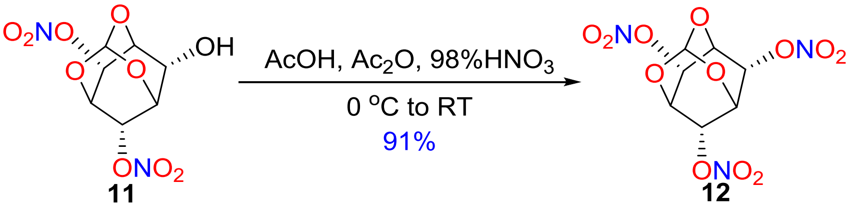

In a reaction flask, acetic acid (0.9 mL) and acetic anhydride (0.3 mL) were combined, and then HNO_3 (0.36 mL, 98%) was added while maintaining the reaction temperature below 5 °C (0 °C in this case). The reaction mixture was stirred for 20 minutes. Then, compound **11** (140 mg, 0.5 mmol) was added slowly in portions. After stirring for 4 h at room temperature, the reaction mixture was poured into a mixture of ice and water (10 mL). Ethyl acetate (10 mL) was added, and the mixture was extracted with EtOAc (10 mL × 3). The organic layer was washed with brine, dried with Na_2SO_4, and filtered. The solvent was then removed using a high-vacuum rotary evaporator, and the remaining residue was subjected to purification by flash column chromatography on silica gel (1:12 ethyl acetate/petroleum ether). This process yielded product **12** (147 mg, 91%) as a white

solid.

^{1}H NMR (500 MHz, Acetone-*d*$_6$) δ 5.92 (s, 1H), 5.86 (dd, *J* = 4.69, 2.70 Hz, 3H), 4.98 (td, *J* = 2.89, 1.55 Hz, 3H).

^{13}C NMR (126 MHz, Acetone-*d*$_6$) δ 103.43, 73.01, 66.35.

IR (thin film, υ cm^{-1}): 3008, 2909, 1657, 1643, 1304, 1272, 1156, 1035, 1000, 979, 951, 914, 859, 818, 768, 750, 736, 723, 678, 646, 606, 586.

EA: Anal. Calcd for $C_7H_7N_3O_{12}$: C, 25.86; H, 2.17; N, 12.92. Found: C, 25.94; H, 2.18; N, 12.89.

(1*R*,3*r*,5*S*,6*S*,7*s*,8*R*)-6,8-bis(*tert*-butyldimethylsiloxyl)-9,9-dinitro-2,4,10-trioxaadamantane (13)

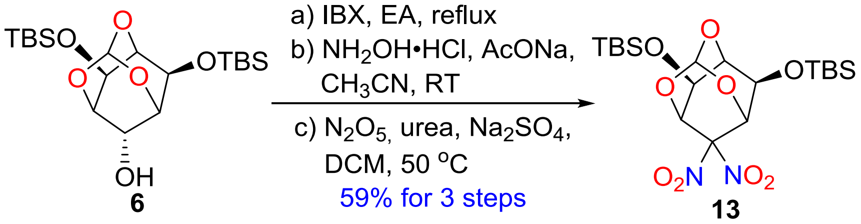

In a reaction flask, 2-iodoxybenzoic acid (1.42 g, 5.1 mmol) was added to a solution of alcohol **6** (710 mg, 1.7 mmol) in ethyl acetate (30 mL), and the mixture was stirred magnetically at 80 °C. After 8 h of reaction, the resulting suspension was filtered through celite and washed with ethyl acetate (20 mL). The filtrate was then concentrated under vacuum and the crude product ketone was used in the next step without further purification.

The ketone was dissolved in acetonitrile (15 mL) and treated with hydroxylamine hydrochloride (236 mg, 3.4 mmol) and sodium acetate (417 mg, 5.1 mmol). The resulting mixture was stirred for 12 h. Brine (20 mL) was added, and the mixture was extracted with ethyl acetate (10 mL × 3). The combined organic layer was dried over anhydrous Na_2SO_4, filtered and concentrated under reduced pressure.

A mixture of the crude product, urea (305 mg, 5.1 mmol), Na_2SO_4 (5 g), and CH_2Cl_2 (20 mL) was stirred and heated to 50 °C. A solution of N_2O_5 (550 mg, 5.1 mmol) in CH_2Cl_2 (10 mL) was added dropwise over 5 min, during which time a green color initially appeared and then faded as more N_2O_5 was added. The reaction mixture was stirred for an additional 2 hours and then poured into an ice-cold saturated solution of $NaHCO_3$ (10 mL). The organic layer was washed with brine, dried with Na_2SO_4, and filtered. The solvent was removed under vacuum, and the resulting residue was purified by flash column chromatography (1:8 ethyl acetate/petroleum ether) on silica gel to yield **13** (378 mg, 59% over three steps) as a white solid.

^{1}H NMR (500 MHz, Acetone-*d*$_6$) δ 5.49 – 5.43 (m, 1H), 4.96 (t, *J* = 1.67 Hz, 2H), 4.05 (q, *J* = 1.58 Hz, 2H), 3.82 (p, *J* = 1.82 Hz, 1H), 0.76 (s, 18H), -0.02 (d, *J* = 15.45 Hz, 12H).

^{13}C NMR (126 MHz, Acetone-*d*$_6$) δ 110.63, 103.66, 74.84, 72.92, 63.92, 26.00, 18.60, -4.75, -4.86.

IR (thin film, υ cm^{-1}): 2953, 2929, 2896, 2857, 1593, 1561, 1464, 1387, 1260, 1251, 1163, 1123, 1011, 976, 926, 900, 821, 775, 741, 681, 669, 567.

EA: Anal. Calcd for $C_{19}H_{36}N_2O_9Si_2$: C, 46.32; H, 7.37; N, 5.69. Found: C, 46.44; H, 7.38; N, 5.68.

(1*R*,3*r*,5*S*,6*S*,7*s*,8*R*)-9,9-dinitro-2,4,10-trioxaadamantane-6,8-diyl dinitrate (14)

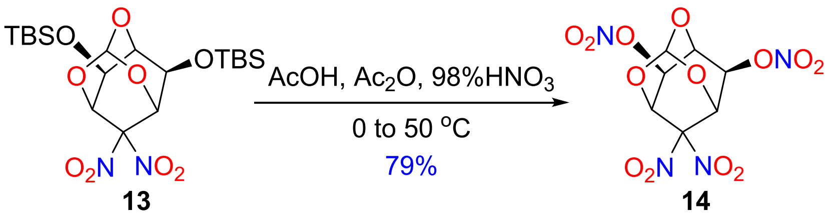

In a reaction flask, acetic acid (1.2 mL) and acetic anhydride (0.4 mL) were combined, and then HNO_3 (0.48mL, 98%) was added while maintaining the reaction temperature below 5 °C (0 °C in this case). The reaction mixture was stirred for 20 minutes. Then, compound **13** (172 mg, 0.35 mol) was added slowly in portions. After stirring for 7 h at 50 °C, the reaction mixture was poured into a mixture of ice and water (10 mL). Ethyl acetate (10 mL) was added, and the mixture was extracted with EtOAc (10 mL × 3). The organic layer was washed with brine, dried with Na_2SO_4, and filtered. The solvent was then removed using a high-vacuum rotary evaporator, and the remaining residue was subjected to purification by flash column chromatography on silica gel (1:10 ethyl acetate/petroleum ether). This process yielded product **14** (98 mg, 79%) as a white solid.

^{1}H NMR (500 MHz, Acetone-d_6) δ 5.94 (dt, J = 3.28, 1.40 Hz, 3H), 5.90 (q, J = 1.61 Hz, 2H), 5.01 (p, J = 1.82 Hz, 1H).

^{13}C NMR (126 MHz, Acetone-d_6) δ 109.74, 103.56, 71.10, 69.25, 67.77.

IR (thin film, υ cm^{-1}): 1674, 1637, 1589, 1568, 1284, 1164, 1073, 1001, 987, 929, 905, 882, 863, 825, 793, 768, 741, 710, 678, 644.

EA: Anal. Calcd for $C_7H_6N_4O_{13}$: C, 23.74; H, 1.71; N, 15.82. Found: C, 23.78; H, 1.72; N, 15.79.

(1*R*,3*r*,5*S*,6*R*,7*r*,8*S*)-9,9-dinitro-2,4,10-trioxaadamantane-6,8-diyl dinitrate (15)

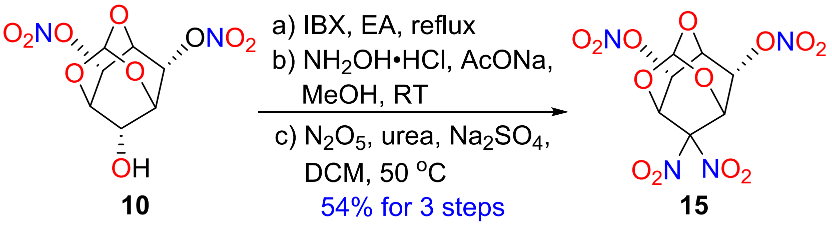

In a reaction flask, 2-iodoxybenzoic acid (0.84 g, 3.0 mmol) was added to a solution of alcohol **10** (280 mg, 1.0 mmol) in ethyl acetate (20 mL), and the mixture was stirred magnetically at 80 °C. After 8 h of reaction, the resulting suspension was filtered through celite and washed with ethyl acetate (20 mL). The filtrate was then concentrated under vacuum and the crude product ketone was used in the next step without further purification.

The ketone was dissolved in methanol (15 mL) and treated with hydroxylamine hydrochloride (139 mg, 2.0 mmol) and sodium acetate (246 mg, 3.0 mmol). The resulting mixture was stirred for 12 h. Brine (20 mL) was added, and the mixture was extracted with ethyl acetate (30 mL × 3). The combined organic layer was dried over anhydrous Na_2SO_4, filtered and concentrated under reduced pressure.

A mixture of the crude product, urea (180 mg, 3.0 mmol), Na_2SO_4 (5 g), and CH_2Cl_2 (20 mL) was stirred and heated to 50 °C. A solution of N_2O_5 (325 mg, 3.0 mmol) in CH_2Cl_2 (10 mL) was added dropwise over 5 min, during which time a green color initially appeared and then faded as more N_2O_5 was added. The reaction mixture was stirred for an additional 30 min and then poured into an ice-cold saturated solution of $NaHCO_3$ (30 mL). The organic layer was washed with brine (10 mL),

dried with Na_2SO_4, and filtered. The solvent was removed under vacuum, and the resulting residue was purified by flash column chromatography (1:8 ethyl acetate/petroleum ether) on silica gel to yield **15** (192 mg, 54% over three steps) as a white solid.

^{1}H NMR (500 MHz, Acetone-*d*$_6$): δ: 6.22 (s, 2H), 6.10 (s, 1H), 6.03 (s, 2H), 5.05 (s, 1H).

^{13}C NMR (126 MHz, Acetone-*d*$_6$): δ: 108.53, 102.72, 73.27, 67.80, 64.87.

IR (thin film, υ cm^{-1}): 1660, 1591, 1279, 1156, 995, 951, 825, 805, 745, 676.

EA: Anal. Calcd for $C_7H_6N_4O_{13}$: C, 23.74; H, 1.71; N, 15.82. Found: C, 23.74; H, 1.71; N, 15.78.

(1*S*,3*R*,5*R*,6*S*,7*S*,8*R*,9*R*)-8,9-bis(*tert*-butyldimethylsiloxy)-2,4,10-trioxaadamantan-6-ol (S1)

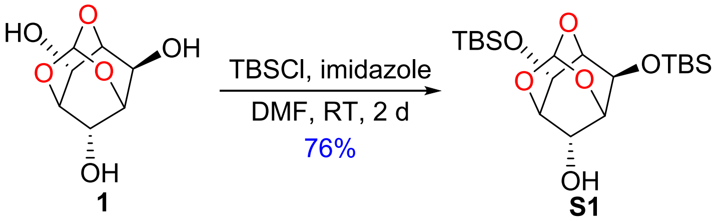

A flame dried round bottom flask equipped with a stirring bar was charged with DMF (20 mL), **1** (1.9 g, 10.0 mmol) and imidazole (1.5 g, 22.0 mmol) at 0 °C, then TBSCl (4.18 g, 22.0 mmol) was added. The resulting mixture was stirred for 2 days at room temperature and the reaction was terminated by addition of a saturated $NaHCO_3$ solution and extracted with EtOAc. The organic phases were combined and washed with water, then dried over anhydrous Na_2SO_4, filtered and concentrated under reduced pressure. The residue was then purified by flash column chromatography on silica gel (1:25 ethyl acetate/petroleum) to afford the products **S1** (3.18 g, 76%) as a white solid.

^{1}H NMR (500 MHz, Chloroform-*d*) δ 5.48 (d, J = 1.31 Hz, 1H), 4.57 (dt, J = 4.40, 2.24 Hz, 1H), 4.42 (td, J = 5.57, 2.67 Hz, 1H), 4.25 (q, J = 1.72 Hz, 1H), 4.14 (td, J = 3.30, 1.56 Hz, 2H), 4.02 (dq, J = 3.96, 1.90 Hz, 1H), 3.81 (d, J = 10.07 Hz, 1H), 0.94 (s, 9H), 0.90 (s, 9H), 0.17 (s, 3H), 0.15 (s, 3H), 0.14 (s, 6H).

^{13}C NMR (126 MHz, Chloroform-*d*) δ 102.49, 75.28, 74.68, 69.63, 68.95, 68.70, 60.76, 26.06, 25.67, 18.53, 17.86, -4.49, -4.57, -4.94, -5.19.

(1*R*,3*S*,5*R*,7*S*,8*R*,9*R*)-8,9-bis(*tert*-butyldimethylsiloxy)-2,4,10-trioxaadamantan-6-one (16)

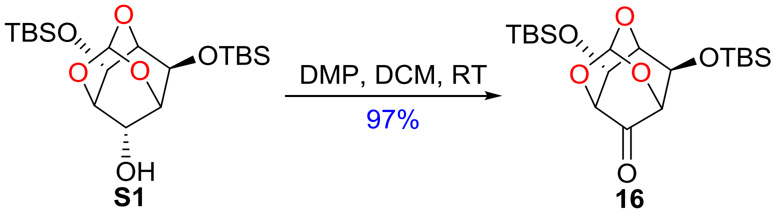

To a solution of **S1** (2.09 g, 5.0 mmol) in DCM (40.0 mL) was added Dess–Martin periodinane (6.36 g, 15.0 mmol) in one portion at 0 °C. The reaction mixture was stirred for 15 min at 0 °C before it was warmed to room temperature and stirred for 8 h. The reaction mixture was quenched with saturated aqueous $NaHCO_3$ (15.0 mL) and saturated aqueous $Na_2S_2O_3$ (15.0 mL) and extracted with DCM (3 × 15 mL). The combined organic layer was washed with brine (15 mL), dried over

anhydrous Na_2SO_4, filtered and concentrated under reduced pressure. The resulting residue was purified by flash column chromatography (1:20 ethyl acetate/petroleum ether) on silica gel to yield **16** (2.01 g, 97%) as a white solid.

^{1}H NMR (500 MHz, Chloroform-*d*) δ 5.62 (d, *J* = 1.42 Hz, 1H), 4.50 (t, *J* = 4.40 Hz, 1H), 4.21 (q, *J* = 1.97 Hz, 1H), 4.16 (dt, *J* = 4.46, 1.58 Hz, 1H), 4.09 (dd, *J* = 4.21, 1.96 Hz, 1H), 4.00 (q, *J* = 1.77 Hz, 1H), 0.91 (s, 10H), 0.82 (s, 9H), 0.10 (s, 9H), 0.08 (d, *J* = 8.13 Hz, 10H).

^{13}C NMR (126 MHz, Chloroform-*d*) δ 199.46, 102.80, 82.27, 78.91, 74.55, 65.77, 65.56, 25.88, 25.49, 18.39, 17.80, -4.66, -4.80, -4.86, -5.00.

IR (thin film, υ cm^{-1}): 2953, 2928, 2894, 2856, 1756, 1471, 1251, 1160, 1122, 1097, 999, 972, 946, 835, 773, 673.

EA: Anal. Calcd for $C_{19}H_{36}O_6Si_2$: C, 54.77; H, 8.71. Found: C, 54.86; H, 8.72.

HRMS (ESI): *m/z* calcd for $C_{19}H_{36}O_6Si_2H^+$ $[M+H]^+$ 417.2129, found 417.2125.

(1*R*,3*S*,5*S*,6*S*,7*R*,8*S*)-6,8-bis(*tert*-butyldimethylsiloxy)-9,9-dinitro-2,4,10-trioxaadamantane (17)

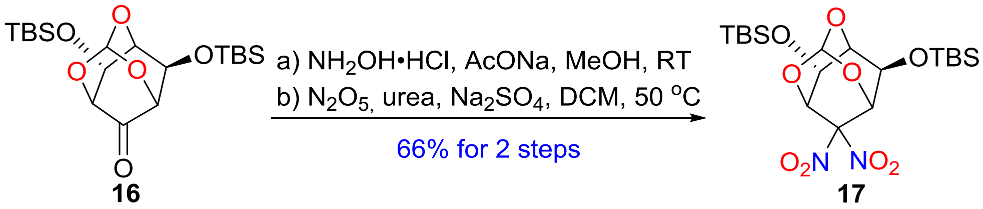

The ketone **16** (2.08 g, 5.0 mmol) was dissolved in methanol (30 mL) and treated with hydroxylamine hydrochloride (695 mg, 10.0 mmol) and sodium acetate (1.23 g, 15.0 mmol). The resulting mixture was stirred for 12 h. Brine (20 mL) was added, and the mixture was extracted with ethyl acetate (30 mL × 3). The combined organic layer was dried over anhydrous Na_2SO_4, filtered and concentrated under reduced pressure.

A mixture of the crude product, urea (901 mg, 15.0 mmol), Na_2SO_4 (5 g), and CH_2Cl_2 (30 mL) was stirred and heated to 50 °C. A solution of N_2O_5 (1.62 g, 15.0 mmol) in CH_2Cl_2 (15 mL) was added dropwise over 5 min, during which time a green color initially appeared and then faded as more N_2O_5 was added. The reaction mixture was stirred for an additional 30 min and then poured into an ice-cold saturated solution of $NaHCO_3$ (30 mL). The organic layer was washed with brine (10 mL), dried with Na_2SO_4, and filtered. The solvent was removed under vacuum, and the resulting residue was purified by flash column chromatography (1:8 ethyl acetate/petroleum ether) on silica gel to yield **15** (1.63 g, 66% over two steps) as a white solid.

^{1}H NMR (500 MHz, Chloroform-*d*) δ 5.52 (d, *J* = 1.33 Hz, 1H), 5.25 (ddd, *J* = 4.12, 2.68, 1.27 Hz, 1H), 5.04 (q, *J* = 2.08 Hz, 1H), 4.62 (t, *J* = 4.27 Hz, 1H), 4.41 (q, *J* = 1.74 Hz, 1H), 3.97 (dd, *J* = 4.32, 1.95 Hz, 1H), 0.96 (s, 9H), 0.83 (s, 9H), 0.20 (d, *J* = 15.74 Hz, 6H), 0.10 (s, 6H).

^{13}C NMR (126 MHz, Chloroform-*d*) δ 109.73, 102.25, 72.83, 71.81, 70.90, 65.88, 62.27, 25.81, 25.43, 18.25, 17.75, -4.65, -4.88, -5.34, -5.38.

IR (thin film, υ cm^{-1}): 2949, 2931, 2887, 2859, 1591, 1569, 1254, 1165, 1146, 1128, 1106, 1021, 1008, 946, 911, 876, 836, 797, 776, 739, 708, 672.

EA: Anal. Calcd for $C_{19}H_{36}N_2O_9Si_2$: C, 46.32; H, 7.37; N, 5.69. Found: C, 46.40; H, 7.38; N, 5.68.

HRMS (ESI): *m/z* calcd for $C_{19}H_{36}N_2O_9Si_2H^+$ $[M+H]^+$ 493.2038, found 493.2046.

(1*R*,3*S*,5*S*,6*S*,7*R*,8*S*)-9,9-dinitro-2,4,10-trioxaadamantane-6,8-diyl dinitrate (18)

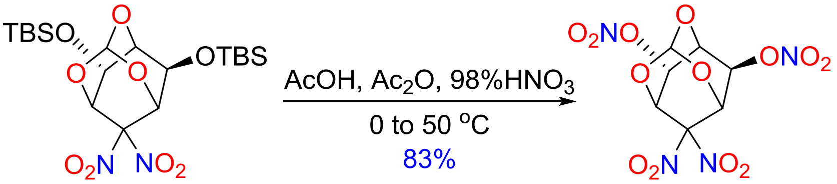

In a reaction flask, acetic acid (1.2 mL) and acetic anhydride (0.4 mL) were combined, and then HNO_3 (0.48mL, 98%) was added while maintaining the reaction temperature below 5 °C (0 °C in this case). The reaction mixture was stirred for 20 minutes. Then, compound **13** (241 mg, 0.49 mol) was added slowly in portions. After stirring for 7 h at 50 °C, the reaction mixture was poured into a mixture of ice and water (10 mL). Ethyl acetate (10 mL) was added, and the mixture was extracted with EtOAc (10 mL × 3). The organic layer was washed with brine, dried with Na_2SO_4, and filtered. The solvent was then removed using a high-vacuum rotary evaporator, and the remaining residue was subjected to purification by flash column chromatography on silica gel (1:10 ethyl acetate/petroleum ether). This process yielded product **14** (144 mg, 83%) as a white solid.

^{1}H NMR (500 MHz, Acetone-d_6) δ 6.27 (t, J = 4.07 Hz, 2H), 6.00 (d, J = 1.33 Hz, 2H), 5.95 (ddd, J = 3.97, 2.60, 1.23 Hz, 2H), 5.93 – 5.89 (m, 2H), 5.70 (q, J = 1.75 Hz, 2H), 5.03 (ddd, J = 3.97, 3.19, 1.79 Hz, 2H), 2.06 (p, J = 2.20 Hz, 1H).

^{13}C NMR (126 MHz, Acetone-d_6) δ 103.06, 72.33, 71.10, 68.53, 68.52, 66.69.

IR (thin film, υ cm^{-1}): 2992, 2967, 2899, 1686, 1643, 1323, 1312, 1286, 1266, 1163, 1057, 1011, 986, 966, 929, 834, 741, 701, 689, 667, 635, 613.

EA: Anal. Calcd for $C_7H_6N_4O_{13}$: C, 23.74; H, 1.71; N, 15.82. Found: C, 23.79; H, 1.72; N, 15.78.

HRMS (ESI): *m/z* calcd for $C_7H_6N_4O_{13}Na^+$ $[M+Na]^+$ 376.9829, found 376.9842.

3 X-ray Crystallographic Analysis

X-ray Crystal Structure and Data of Compound 2

CCDC 2425333

Single crystals of compound **2** suitable for X-ray diffraction analysis were obtained by slow recrystallization from a mixture of acetone and ethanol at room temperature. The result of X-ray diffraction indicates that compound **2** crystallizes in the monoclinic space group P 1 21/c 1 and four moieties per unit cell (Z = 4). The crystal density was determined to be 1.816 g cm^{-3} at 296.15 K.

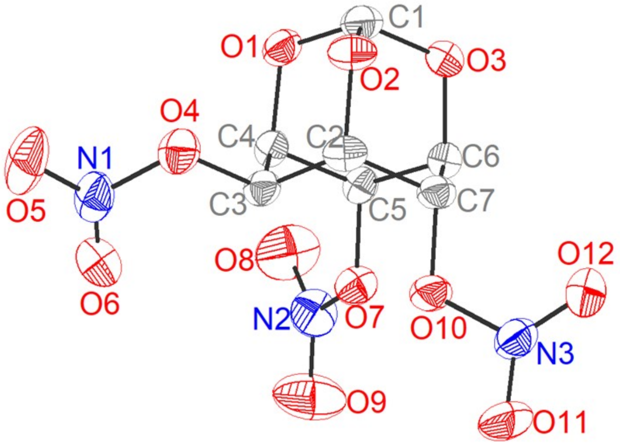

Figure S1. ORTEP diagram of compound **2**. Color code: Carbon (grey), Oxygen (red), Nitrogen (blue).

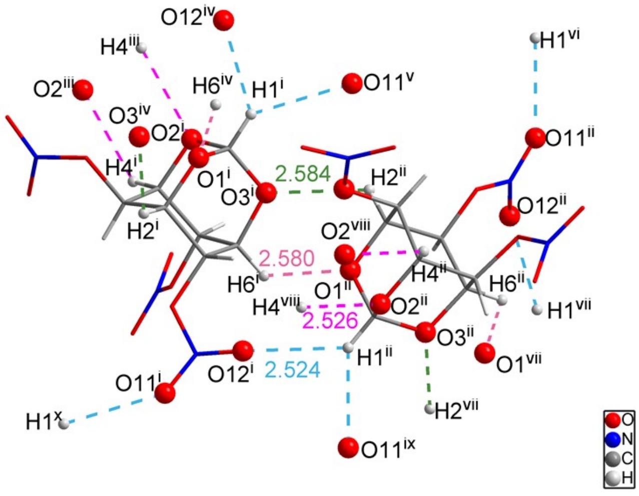

Figure S2. Hydrogen-bond network of compound **2**.

Table S1. Crystal data and structure refinement for compound **2**.

Empirical formula	$C_7H_7N_3O_{12}$
Formula weight	325.16
Temperature	296.15 K

Wavelength	0.71073 Å	
Crystal system	Monoclinic	
Space group	P 1 21/c 1	
Unit cell dimensions	a = 8.730(7) Å	a= 90°.
	b = 8.696(7) Å	b= 101.351(9)°.
	c = 15.978(13) Å	g = 90°.
Volume	1189.4(16) Å^3	
Z	4	
Density (calculated)	1.816 Mg/m^3	
Absorption coefficient	0.179 mm^{-1}	
F(000)	664	
Crystal size	0.14 x 0.12 x 0.12 mm^3	
Theta range for data collection	2.379 to 27.221°.	
Index ranges	-11<=h<=11, -11<=k<=10, -20<=l<=20	
Reflections collected	8955	
Independent reflections	2451 [R(int) = 0.0450]	
Completeness to theta = 25.242°	99.9 %	
Absorption correction	None	
Refinement method	Full-matrix least-squares on F^2	
Data / restraints / parameters	2451 / 0 / 200	
Goodness-of-fit on F^2	1.050	
Final R indices [I>2sigma(I)]	R1 = 0.0352, wR2 = 0.0903	
R indices (all data)	R1 = 0.0411, wR2 = 0.0946	
Extinction coefficient	0.022(2)	
Largest diff. peak and hole	0.284 and -0.219 e.Å^{-3}	

Table S2. Atomic coordinates (x 10^4) and equivalent isotropic displacement parameters (Å^{2}x 10^3) for compound **2**. U(eq) is defined as one third of the trace of the orthogonalized U^{ij} tensor.

atom	x	y	z	U(eq)
O(7)	3399(1)	1687(1)	4848(1)	33(1)
O(2)	-323(1)	3737(1)	4255(1)	33(1)
O(3)	-308(1)	1650(1)	3344(1)	34(1)
O(10)	3749(1)	2398(1)	3154(1)	38(1)
O(1)	-82(1)	4140(1)	2857(1)	37(1)
O(4)	1732(1)	6214(1)	4020(1)	41(1)
O(6)	3522(2)	6343(2)	5242(1)	56(1)
O(5)	2072(2)	8313(1)	4768(1)	66(1)
N(3)	4437(2)	1393(2)	2644(1)	41(1)

O(11)	5822(1)	1493(2)	2787(1)	64(1)
O(8)	2705(2)	1391(2)	6120(1)	67(1)
O(12)	3610(2)	582(2)	2152(1)	72(1)
N(1)	2533(2)	7014(2)	4751(1)	45(1)
O(9)	5120(2)	1233(2)	6003(1)	73(1)
N(2)	3757(2)	1412(2)	5740(1)	44(1)
C(4)	1332(2)	3640(2)	4568(1)	27(1)
C(5)	1759(2)	1939(2)	4518(1)	27(1)
C(3)	2164(2)	4609(2)	3996(1)	29(1)
C(7)	2065(2)	2433(2)	2968(1)	30(1)
C(6)	1357(2)	1434(2)	3583(1)	29(1)
C(2)	1594(2)	4102(2)	3072(1)	32(1)
C(1)	-734(2)	3199(2)	3409(1)	34(1)

Table S3. Bond lengths [Å] and angles [°] for compound **2**.

O(7)-N(2)	1.4185(19)
O(7)-C(5)	1.4418(19)
O(2)-C(4)	1.4353(19)
O(2)-C(1)	1.408(2)
O(3)-C(6)	1.4414(19)
O(3)-C(1)	1.407(2)
O(10)-N(3)	1.4074(17)
O(10)-C(7)	1.4417(19)
O(1)-C(2)	1.435(2)
O(1)-C(1)	1.4031(18)
O(4)-N(1)	1.420(2)
O(4)-C(3)	1.4477(19)
O(6)-N(1)	1.197(2)
O(5)-N(1)	1.201(2)
N(3)-O(11)	1.189(2)
N(3)-O(12)	1.1885(19)
O(8)-N(2)	1.196(2)
O(9)-N(2)	1.192(2)
C(4)-H(4)	0.9800
C(4)-C(5)	1.532(2)
C(4)-C(3)	1.5279(19)
C(5)-H(5)	0.9800
C(5)-C(6)	1.529(2)

C(3)-H(3)	0.9800
C(3)-C(2)	1.527(2)
C(7)-H(7)	0.9800
C(7)-C(6)	1.531(2)
C(7)-C(2)	1.527(2)
C(6)-H(6)	0.9800
C(2)-H(2)	0.9800
C(1)-H(1)	0.9800

N(2)-O(7)-C(5)	113.56(10)
C(1)-O(2)-C(4)	111.32(10)
C(1)-O(3)-C(6)	111.84(10)
N(3)-O(10)-C(7)	115.26(11)
C(1)-O(1)-C(2)	110.91(10)
N(1)-O(4)-C(3)	113.95(11)
O(11)-N(3)-O(10)	112.12(13)
O(12)-N(3)-O(10)	118.59(13)
O(12)-N(3)-O(11)	129.29(14)
O(6)-N(1)-O(4)	118.32(13)
O(6)-N(1)-O(5)	130.14(16)
O(5)-N(1)-O(4)	111.54(16)
O(8)-N(2)-O(7)	118.25(14)
O(9)-N(2)-O(7)	112.51(13)
O(9)-N(2)-O(8)	129.24(15)
O(2)-C(4)-H(4)	110.4
O(2)-C(4)-C(5)	106.10(10)
O(2)-C(4)-C(3)	108.90(11)
C(5)-C(4)-H(4)	110.4
C(3)-C(4)-H(4)	110.4
C(3)-C(4)-C(5)	110.61(11)
O(7)-C(5)-C(4)	111.23(10)
O(7)-C(5)-H(5)	109.2
O(7)-C(5)-C(6)	109.53(10)
C(4)-C(5)-H(5)	109.2
C(6)-C(5)-C(4)	108.45(11)
C(6)-C(5)-H(5)	109.2
O(4)-C(3)-C(4)	111.00(11)
O(4)-C(3)-H(3)	110.6

O(4)-C(3)-C(2)	105.50(11)
C(4)-C(3)-H(3)	110.6
C(2)-C(3)-C(4)	108.43(12)
C(2)-C(3)-H(3)	110.6
O(10)-C(7)-H(7)	109.8
O(10)-C(7)-C(6)	112.31(11)
O(10)-C(7)-C(2)	106.45(11)
C(6)-C(7)-H(7)	109.8
C(2)-C(7)-H(7)	109.8
C(2)-C(7)-C(6)	108.52(11)
O(3)-C(6)-C(5)	104.35(10)
O(3)-C(6)-C(7)	105.63(11)
O(3)-C(6)-H(6)	110.6
C(5)-C(6)-C(7)	114.87(12)
C(5)-C(6)-H(6)	110.6
C(7)-C(6)-H(6)	110.6
O(1)-C(2)-C(3)	110.32(11)
O(1)-C(2)-C(7)	106.25(11)
O(1)-C(2)-H(2)	110.2
C(3)-C(2)-C(7)	109.64(11)
C(3)-C(2)-H(2)	110.2
C(7)-C(2)-H(2)	110.2
O(2)-C(1)-H(1)	107.7
O(3)-C(1)-O(2)	111.60(11)
O(3)-C(1)-H(1)	107.7
O(1)-C(1)-O(2)	110.57(12)
O(1)-C(1)-O(3)	111.47(12)
O(1)-C(1)-H(1)	107.7

Symmetry transformations used to generate equivalent atoms:

Table S4. Anisotropic displacement parameters (Å2x 103) for compound **2**. The anisotropic displacement factor exponent takes the form: -2p2[h2 a*2U11 + ... + 2 h k a* b* U12]

	U^{11}	U^{22}	U^{33}	U^{23}	U^{13}	U^{12}
O(7)	34(1)	37(1)	28(1)	5(1)	6(1)	6(1)
O(2)	28(1)	32(1)	41(1)	-2(1)	13(1)	2(1)
O(3)	27(1)	34(1)	42(1)	-6(1)	6(1)	-4(1)
O(10)	27(1)	54(1)	35(1)	-10(1)	8(1)	2(1)

O(1)	28(1)	44(1)	38(1)	11(1)	3(1)	5(1)
O(4)	44(1)	24(1)	54(1)	4(1)	9(1)	2(1)
O(6)	48(1)	51(1)	65(1)	-14(1)	5(1)	-10(1)
O(5)	94(1)	24(1)	94(1)	-6(1)	51(1)	-6(1)
N(3)	35(1)	39(1)	52(1)	-3(1)	16(1)	4(1)
O(11)	31(1)	68(1)	97(1)	-14(1)	23(1)	3(1)
O(8)	66(1)	105(1)	32(1)	10(1)	18(1)	14(1)
O(12)	50(1)	70(1)	96(1)	-46(1)	14(1)	0(1)
N(1)	51(1)	28(1)	63(1)	-7(1)	28(1)	-12(1)
O(9)	48(1)	111(1)	52(1)	24(1)	-7(1)	7(1)
N(2)	50(1)	49(1)	30(1)	5(1)	3(1)	5(1)
C(4)	29(1)	25(1)	29(1)	-2(1)	8(1)	1(1)
C(5)	29(1)	25(1)	28(1)	1(1)	8(1)	1(1)
C(3)	27(1)	22(1)	38(1)	2(1)	7(1)	1(1)
C(7)	24(1)	40(1)	26(1)	-3(1)	5(1)	1(1)
C(6)	28(1)	27(1)	31(1)	-4(1)	5(1)	1(1)
C(2)	27(1)	37(1)	32(1)	9(1)	7(1)	1(1)
C(1)	25(1)	36(1)	42(1)	0(1)	6(1)	2(1)

Table S5. Hydrogen coordinates (x 104) and isotropic displacement parameters (Å2x 10 3) for compound **2**.

	x	y	z	U(eq)
H(4)	1598	4004	5159	33
H(5)	1146	1330	4850	32
H(3)	3299	4487	4163	35
H(7)	1676	2095	2379	36
H(6)	1630	351	3528	35
H(2)	2036	4768	2685	38
H(1)	-1873	3266	3236	41

_

Table S6. Torsion angles [°] for compound **2**.

O(7)-C(5)-C(6)-O(3)	176.59(9)
O(7)-C(5)-C(6)-C(7)	-68.25(14)
O(2)-C(4)-C(5)-O(7)	-177.84(9)
O(2)-C(4)-C(5)-C(6)	61.66(13)
O(2)-C(4)-C(3)-O(4)	62.15(14)
O(2)-C(4)-C(3)-C(2)	-53.29(13)
O(10)-C(7)-C(6)-O(3)	177.17(10)

O(10)-C(7)-C(6)-C(5)	62.76(15)
O(10)-C(7)-C(2)-O(1)	178.08(10)
O(10)-C(7)-C(2)-C(3)	-62.71(13)
O(4)-C(3)-C(2)-O(1)	-66.05(13)
O(4)-C(3)-C(2)-C(7)	177.28(10)
N(3)-O(10)-C(7)-C(6)	98.94(14)
N(3)-O(10)-C(7)-C(2)	-142.42(11)
N(1)-O(4)-C(3)-C(4)	79.74(15)
N(1)-O(4)-C(3)-C(2)	-163.01(10)
N(2)-O(7)-C(5)-C(4)	88.24(13)
N(2)-O(7)-C(5)-C(6)	-151.90(12)
C(4)-O(2)-C(1)-O(3)	60.51(13)
C(4)-O(2)-C(1)-O(1)	-64.17(14)
C(4)-C(5)-C(6)-O(3)	-61.85(12)
C(4)-C(5)-C(6)-C(7)	53.30(15)
C(4)-C(3)-C(2)-O(1)	52.92(14)
C(4)-C(3)-C(2)-C(7)	-63.75(14)
C(5)-O(7)-N(2)-O(8)	1.07(19)
C(5)-O(7)-N(2)-O(9)	-178.84(13)
C(5)-C(4)-C(3)-O(4)	178.38(11)
C(5)-C(4)-C(3)-C(2)	62.94(14)
C(3)-O(4)-N(1)-O(6)	3.93(18)
C(3)-O(4)-N(1)-O(5)	-175.92(12)
C(3)-C(4)-C(5)-O(7)	64.21(14)
C(3)-C(4)-C(5)-C(6)	-56.30(14)
C(7)-O(10)-N(3)-O(11)	175.15(13)
C(7)-O(10)-N(3)-O(12)	-5.3(2)
C(6)-O(3)-C(1)-O(2)	-62.24(14)
C(6)-O(3)-C(1)-O(1)	61.94(15)
C(6)-C(7)-C(2)-O(1)	-60.82(14)
C(6)-C(7)-C(2)-C(3)	58.39(14)
C(2)-O(1)-C(1)-O(2)	62.63(14)
C(2)-O(1)-C(1)-O(3)	-62.13(15)
C(2)-C(7)-C(6)-O(3)	59.76(13)
C(2)-C(7)-C(6)-C(5)	-54.65(15)
C(1)-O(2)-C(4)-C(5)	-59.62(13)
C(1)-O(2)-C(4)-C(3)	59.48(14)
C(1)-O(3)-C(6)-C(5)	61.70(13)

C(1)-O(3)-C(6)-C(7)	-59.79(14)
C(1)-O(1)-C(2)-C(3)	-57.73(15)
C(1)-O(1)-C(2)-C(7)	61.03(14)

Symmetry transformations used to generate equivalent atoms:

X-ray Crystal Structure and Data of Compound 5

CCDC 2425240

Single crystals of compound **5** suitable for X-ray diffraction analysis were obtained by slow recrystallization from a mixture of acetone and ethanol at room temperature. The result of X-ray diffraction indicates that compound **5** crystallizes in the monoclinic space group $P2_1/c$ and four moieties per unit cell (Z = 4). The crystal density was determined to be 1.865 g cm^{-3} at 293.15 K.

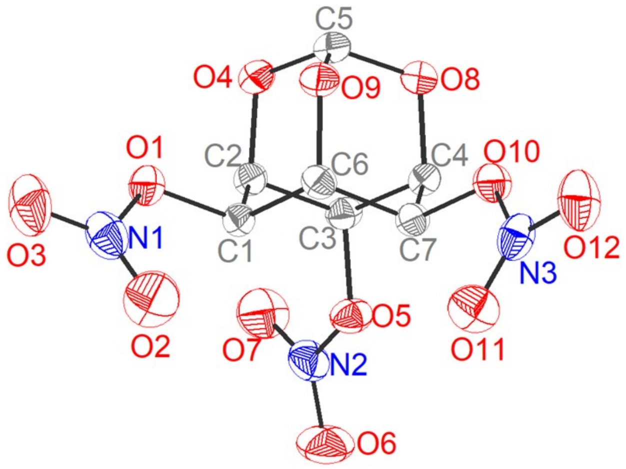

Figure S3. ORTEP diagram of Compound **5**. Color code: Carbon (grey), Oxygen (red), Nitrogen (blue).

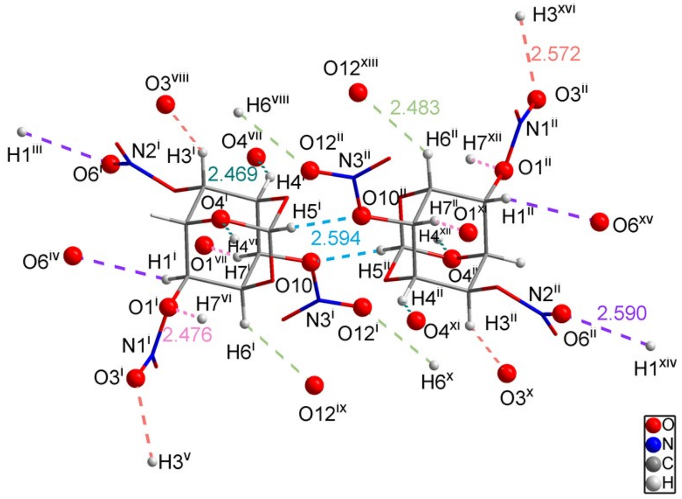

Figure S4. Hydrogen-bond network of compound **5**.

Table S7. Crystal data and structure refinement for Compound **5.**

Empirical formula	$C_7H_7N_3O_{12}$
Formula weight	325.16
Temperature/K	293.15
Crystal system	monoclinic
Space group	$P2_1/c$
a/Å	15.062(5)
b/Å	5.9269(16)
c/Å	14.178(4)
α/°	90
β/°	113.806(10)
γ/°	90
Volume/Å^3	1158.0(6)
Z	4
ρ_{calc} g/cm^3	1.865
μ/mm^{-1}	0.184
F(000)	664.0
Crystal size/mm^3	0.16 × 0.15 × 0.13
Radiation	MoKα (λ = 0.71073)
2Θ range for data collection/°	5.762 to 63.464
Index ranges	$-22 \leq h \leq 15$, $-8 \leq k \leq 8$, $-20 \leq l \leq 20$
Reflections collected	13726
Independent reflections	3903 [R_{int} = 0.0477, R_{sigma} = 0.0483]
Data/restraints/parameters	3903/0/199
Goodness-of-fit on F^2	1.103
Final R indexes [I>=2σ (I)]	R_1 = 0.0594, wR_2 = 0.1104
Final R indexes [all data]	R_1 = 0.1008, wR_2 = 0.1294
Largest diff. peak/hole / e Å^{-3}	0.35/-0.29

Table S8. Fractional Atomic Coordinates (×10^4) and Equivalent Isotropic Displacement Parameters (Å^2×10^3) for Compound **5**. U_{eq} is defined as 1/3 of the trace of the orthogonalised U_{IJ} tensor.

**Atom**	***x***	***y***	***z***	**U(eq)**
O(1)	1949.7(11)	-2175(2)	2264.9(11)	32.9(3)
N(1)	1702.3(15)	-2277(4)	1197.4(15)	41.8(5)
C(1)	2144.0(14)	74(3)	2700.8(14)	25.1(4)
N(2)	632.0(13)	3883(3)	3647.0(15)	36.9(4)
C(2)	2054.4(14)	-163(3)	3727.0(14)	25.4(4)
O(2)	1691.5(17)	-555(4)	756.9(14)	63.5(6)
N(3)	4432.1(14)	5112(3)	2859.4(15)	34.8(4)

Atom	x	y	z	U(eq)
O(3)	1528.9(15)	-4164(3)	875.1(15)	59.2(5)
C(3)	2208.3(14)	2146(3)	4254.1(15)	25.7(4)
O(4)	2809.8(10)	-1608(2)	4402.8(11)	31.0(3)
C(4)	3224.8(14)	2977(3)	4429.7(15)	25.8(4)
O(5)	1549.7(10)	3843(2)	3609.9(11)	31.2(3)
C(5)	3736.4(15)	-777(3)	4566.4(16)	31.4(4)
O(6)	153.8(13)	5457(3)	3182.9(16)	57.4(5)
C(6)	3173.8(13)	844(3)	2920.9(15)	26.9(4)
O(7)	432.2(13)	2423(3)	4104.7(15)	52.3(5)
C(7)	3314.4(14)	3199(3)	3407.0(15)	26.7(4)
O(8)	3901.9(10)	1330(2)	5056.7(11)	31.7(3)
O(9)	3854.7(10)	-686(2)	3638.2(11)	32.1(3)
O(10)	4293.7(10)	3991(3)	3672.7(11)	33.9(3)
O(11)	3744.8(13)	5406(3)	2068.8(13)	46.3(4)
O(12)	5262.8(13)	5620(3)	3107.6(15)	52.5(5)

Table S9. Anisotropic Displacement Parameters (Å^2×10^3) for Compound **5**. The Anisotropic displacement factor exponent takes the form: $-2\pi^2[h^2a^{*2}U_{11}+2hka^*b^*U_{12}+\ldots]$.

Atom	U_{11}	U_{22}	U_{33}	U_{23}	U_{13}	U_{12}
O(1)	36.8(8)	27.2(7)	31.5(7)	-5.2(6)	10.4(6)	-1.2(6)
N(1)	37.7(10)	50.0(12)	35.7(10)	-11.2(9)	12.7(8)	-1.9(9)
C(1)	22.4(9)	21.6(9)	29.3(9)	-1.7(7)	8.4(7)	0.4(7)
N(2)	24.9(9)	40.5(10)	43.2(10)	-2.3(8)	11.4(8)	1.8(8)
C(2)	21.8(9)	22.5(9)	30.6(9)	1.8(7)	9.4(8)	0.0(7)
O(2)	90.0(17)	63.0(13)	35.9(9)	3.5(9)	23.7(10)	-5.0(11)
N(3)	37.2(10)	29.0(9)	47.3(11)	-5.2(8)	26.4(9)	-5.6(8)
O(3)	58.5(12)	56.6(12)	61.0(12)	-33.2(10)	22.6(10)	-8.7(9)
C(3)	24.5(9)	24.1(9)	29.0(9)	3.1(7)	11.2(8)	3.2(7)
O(4)	29.3(7)	23.4(7)	35.9(7)	6.8(6)	8.5(6)	-0.6(6)
C(4)	23.9(9)	19.8(8)	30.7(9)	1.1(7)	7.8(8)	0.1(7)
O(5)	25.6(7)	30.8(7)	39.4(8)	6.6(6)	15.5(6)	5.7(6)
C(5)	27.1(10)	24.1(9)	36.6(11)	6.1(8)	6.3(9)	4.2(8)
O(6)	33.6(9)	56.4(12)	76.3(13)	16.2(10)	16.1(9)	19.3(8)
C(6)	20.8(9)	28.0(9)	31.0(10)	1.1(7)	9.4(8)	1.2(7)
O(7)	35.3(9)	59.7(12)	67.9(12)	11.5(9)	27.1(9)	-2.4(8)
C(7)	19.8(8)	24.9(9)	34.4(10)	3.3(7)	10.0(8)	-0.8(7)
O(8)	28.8(7)	26.2(7)	31.3(7)	3.2(6)	2.9(6)	1.0(6)
O(9)	24.6(7)	28.7(7)	40.5(8)	1.1(6)	10.6(6)	6.6(6)
O(10)	23.8(7)	34.4(8)	42.0(8)	6.9(6)	11.9(6)	-4.3(6)

Atom	U_{11}	U_{22}	U_{33}	U_{23}	U_{13}	U_{12}
O(11)	48.1(10)	56.0(11)	38.2(9)	7.0(8)	21.1(8)	-0.3(8)
O(12)	40.8(10)	58.7(11)	68.9(12)	-12.7(9)	33.5(9)	-19.6(9)

Table S10. Bond Lengths for Compound **5**.

Atom	Atom	Length/Å	Atom	Atom	Length/Å
O(1)	N(1)	1.406(2)	N(3)	O(11)	1.192(2)
O(1)	C(1)	1.449(2)	N(3)	O(12)	1.194(2)
N(1)	O(2)	1.193(3)	C(3)	C(4)	1.530(3)
N(1)	O(3)	1.196(3)	C(3)	O(5)	1.449(2)
C(1)	C(2)	1.521(3)	O(4)	C(5)	1.408(3)
C(1)	C(6)	1.523(3)	C(4)	C(7)	1.515(3)
N(2)	O(5)	1.405(2)	C(4)	O(8)	1.432(2)
N(2)	O(6)	1.200(2)	C(5)	O(8)	1.401(2)
N(2)	O(7)	1.191(3)	C(5)	O(9)	1.398(3)
C(2)	C(3)	1.531(3)	C(6)	C(7)	1.533(3)
C(2)	O(4)	1.437(2)	C(6)	O(9)	1.438(2)
N(3)	O(10)	1.417(2)	C(7)	O(10)	1.446(2)

Table S11. Bond Angles for Compound **5**.

Atom	Atom	Atom	Angle/°	Atom	Atom	Atom	Angle/°
N(1)	O(1)	C(1)	114.77(15)	O(5)	C(3)	C(4)	105.34(15)
O(2)	N(1)	O(1)	117.90(19)	C(5)	O(4)	C(2)	111.49(14)
O(2)	N(1)	O(3)	130.2(2)	C(7)	C(4)	C(3)	110.00(15)
O(3)	N(1)	O(1)	111.9(2)	O(8)	C(4)	C(3)	107.25(15)
O(1)	C(1)	C(2)	104.03(15)	O(8)	C(4)	C(7)	109.68(16)
O(1)	C(1)	C(6)	112.14(16)	N(2)	O(5)	C(3)	114.96(15)
C(2)	C(1)	C(6)	107.93(15)	O(8)	C(5)	O(4)	111.07(17)
O(6)	N(2)	O(5)	112.00(19)	O(9)	C(5)	O(4)	110.65(16)
O(7)	N(2)	O(5)	118.49(18)	O(9)	C(5)	O(8)	111.58(16)
O(7)	N(2)	O(6)	129.5(2)	C(1)	C(6)	C(7)	108.03(15)
C(1)	C(2)	C(3)	109.26(15)	O(9)	C(6)	C(1)	109.42(16)
O(4)	C(2)	C(1)	109.92(16)	O(9)	C(6)	C(7)	109.06(15)
O(4)	C(2)	C(3)	106.50(15)	C(4)	C(7)	C(6)	107.57(15)
O(11)	N(3)	O(10)	118.46(18)	O(10)	C(7)	C(4)	104.74(15)
O(11)	N(3)	O(12)	130.5(2)	O(10)	C(7)	C(6)	110.75(16)
O(12)	N(3)	O(10)	111.07(19)	C(5)	O(8)	C(4)	111.09(14)
C(4)	C(3)	C(2)	108.16(16)	C(5)	O(9)	C(6)	111.38(15)
O(5)	C(3)	C(2)	112.49(15)	N(3)	O(10)	C(7)	114.20(15)

Table S12. Torsion Angles for Compound **5**.

A	B	C	D	Angle/°	A	B	C	D	Angle/°
O(1)	C(1)	C(2)	C(3)	-177.91(14)	C(3)	C(4)	O(8)	C(5)	60.4(2)
O(1)	C(1)	C(2)	O(4)	65.56(18)	O(4)	C(2)	C(3)	C(4)	58.51(19)
O(1)	C(1)	C(6)	C(7)	-178.34(15)	O(4)	C(2)	C(3)	O(5)	174.44(15)
O(1)	C(1)	C(6)	O(9)	-59.7(2)	O(4)	C(5)	O(8)	C(4)	-62.4(2)
N(1)	O(1)	C(1)	C(2)	161.74(15)	O(4)	C(5)	O(9)	C(6)	62.6(2)
N(1)	O(1)	C(1)	C(6)	-81.88(19)	C(4)	C(3)	O(5)	N(2)	-158.25(15)
C(1)	O(1)	N(1)	O(2)	1.3(3)	C(4)	C(7)	O(10)	N(3)	-157.35(15)
C(1)	O(1)	N(1)	O(3)	-178.46(19)	O(5)	C(3)	C(4)	C(7)	-60.14(19)
C(1)	C(2)	C(3)	C(4)	-60.17(19)	O(5)	C(3)	C(4)	O(8)	-179.37(14)
C(1)	C(2)	C(3)	O(5)	55.8(2)	O(6)	N(2)	O(5)	C(3)	173.00(18)
C(1)	C(2)	O(4)	C(5)	57.9(2)	C(6)	C(1)	C(2)	C(3)	62.81(19)
C(1)	C(6)	C(7)	C(4)	63.82(19)	C(6)	C(1)	C(2)	O(4)	-53.72(19)
C(1)	C(6)	C(7)	O(10)	177.74(15)	C(6)	C(7)	O(10)	N(3)	86.96(19)
C(1)	C(6)	O(9)	C(5)	-59.5(2)	O(7)	N(2)	O(5)	C(3)	-7.4(3)
C(2)	C(1)	C(6)	C(7)	-64.34(19)	C(7)	C(4)	O(8)	C(5)	-59.0(2)
C(2)	C(1)	C(6)	O(9)	54.3(2)	C(7)	C(6)	O(9)	C(5)	58.5(2)
C(2)	C(3)	C(4)	C(7)	60.35(19)	O(8)	C(4)	C(7)	C(6)	55.48(19)
C(2)	C(3)	C(4)	O(8)	-58.88(19)	O(8)	C(4)	C(7)	O(10)	-62.39(18)
C(2)	C(3)	O(5)	N(2)	84.15(19)	O(8)	C(5)	O(9)	C(6)	-61.6(2)
C(2)	O(4)	C(5)	O(8)	62.8(2)	O(9)	C(5)	O(8)	C(4)	61.5(2)
C(2)	O(4)	C(5)	O(9)	-61.7(2)	O(9)	C(6)	C(7)	C(4)	-55.0(2)
C(3)	C(2)	O(4)	C(5)	-60.3(2)	O(9)	C(6)	C(7)	O(10)	58.9(2)
C(3)	C(4)	C(7)	C(6)	-62.24(19)	O(11)	N(3)	O(10)	C(7)	3.8(3)
C(3)	C(4)	C(7)	O(10)	179.88(15)	O(12)	N(3)	O(10)	C(7)	-176.37(17)

Table S13. Hydrogen Atom Coordinates (Å×10^4) and Isotropic Displacement Parameters (Å^2×10^3) for Compound **5**.

Atom	*x*	*y*	*z*	U(eq)
H(1)	1667.55	1154.37	2252.81	30
H(2)	1415.59	-768.4	3620.99	30
H(3)	2143.96	2024.4	4913.01	31
H(4)	3350.68	4436.63	4784.6	31
H(5)	4218.53	-1834.03	5023.8	38
H(6)	3273.22	896.12	2279.51	32
H(7)	2837.34	4268.2	2951.79	32

X-ray Crystal Structure and Data of Compound 8

CCDC 2425243

Single crystals of compound **8** suitable for X-ray diffraction analysis were obtained by slow recrystallization from a mixture of acetone and ethanol at room temperature. The result of X-ray diffraction indicates that compound **8** crystallizes in the triclinic space group P-1 and two moieties per unit cell (Z = 2). The crystal density was determined to be 1.894 g cm^{-3} at 293 K.

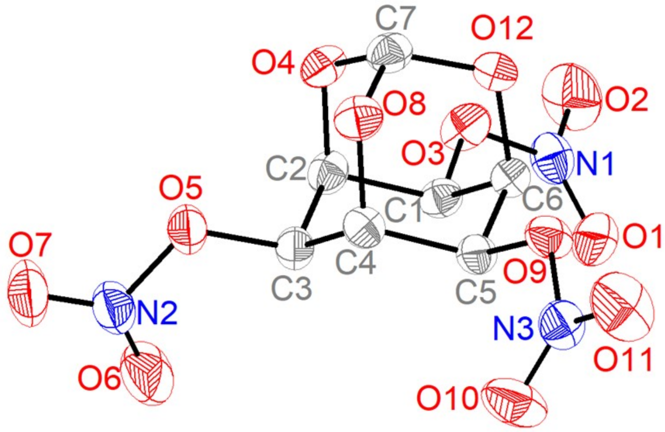

Figure S5. ORTEP diagram of Compound **8**. Color code: Carbon (grey), Oxygen (red), Nitrogen (blue).

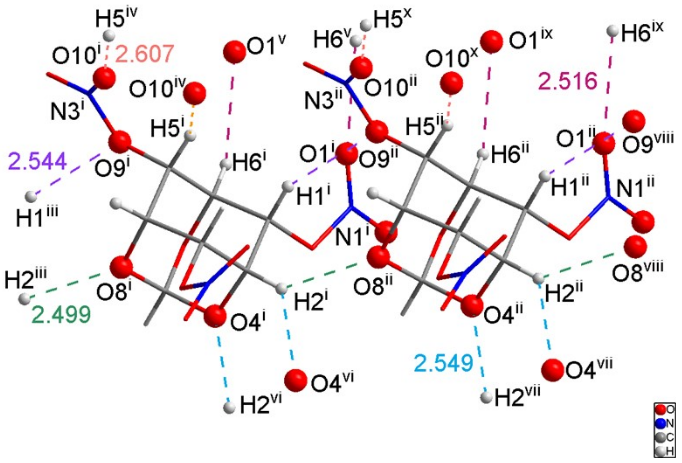

Figure S6. Hydrogen-bond network of compound **8**.

Table S14. Crystal data and structure refinement for Compound **8**.

Empirical formula	$C_7H_7N_3O_{12}$
Formula weight	325.16
Temperature/K	293.00
Crystal system	triclinic
Space group	P-1
a/Å	6.0238(3)
b/Å	8.8265(4)

c/Å	12.0580(7)
α/°	69.746(2)
β/°	89.688(2)
γ/°	72.481(2)
Volume/Å^3	570.07(5)
Z	2
ρ_{calc}g/cm^3	1.894
μ/mm^{-1}	0.187
F(000)	332.0
Crystal size/mm^3	0.13 × 0.12 × 0.11
Radiation	MoKα (λ = 0.71073)
2Θ range for data collection/°	5.146 to 54.938
Index ranges	-7 ≤ h ≤ 7, -11 ≤ k ≤ 11, -15 ≤ l ≤ 15
Reflections collected	15971
Independent reflections	2588 [R_{int} = 0.0347, R_{sigma} = 0.0278]
Data/restraints/parameters	2588/1/200
Goodness-of-fit on F^2	1.034
Final R indexes [I>=2σ (I)]	R_1 = 0.0345, wR_2 = 0.0900
Final R indexes [all data]	R_1 = 0.0374, wR_2 = 0.0927
Largest diff. peak/hole / e Å^{-3}	0.35/-0.25

Table S15. Fractional Atomic Coordinates (×10^4) and Equivalent Isotropic Displacement Parameters (Å^2×10^3) for Compound **8**. U_{eq} is defined as 1/3 of the trace of the orthogonalised U_{IJ} tensor.

**Atom**	***x***	***y***	***z***	**U(eq)**
O(1)	12312(2)	3651.3(14)	5964.0(9)	46.9(3)
N(1)	12670.8(18)	2665.3(14)	6970.1(10)	36.6(3)
C(1)	9775.5(19)	4885.3(14)	7361.4(10)	28.5(2)
O(2)	13993(2)	1243.3(14)	7377.7(12)	61.3(3)
N(2)	8288(2)	9022.2(15)	9011.0(11)	43.0(3)
C(2)	9270(2)	5418.5(15)	8436.0(10)	29.6(3)
O(3)	11428.6(16)	3187.0(12)	7828.1(8)	37.9(2)
N(3)	2452.7(19)	8272.9(14)	4949.6(10)	37.6(3)
C(3)	7607(2)	7244.9(15)	7997.6(10)	30.4(3)
O(4)	8156.0(15)	4323.4(11)	9243.9(7)	33.2(2)
C(4)	5300(2)	7252.7(15)	7473.5(10)	30.3(3)
O(5)	7123.0(17)	7825.1(12)	8974.2(8)	38.2(2)
C(5)	5835(2)	6732.7(14)	6389.3(10)	28.3(2)
O(6)	9661(3)	9284.5(19)	8313.0(12)	69.5(4)
C(6)	7469(2)	4896.6(14)	6828.3(10)	28.2(2)

Atom	x	y	z	U(eq)
O(7)	7678(2)	9570.5(16)	9770.5(11)	60.5(3)
C(7)	6071(2)	4382.2(16)	8695.6(11)	32.9(3)
O(8)	4428.3(15)	6034.4(11)	8327.5(8)	34.7(2)
O(9)	3743.2(16)	6707.5(11)	5820.0(8)	35.8(2)
O(10)	3120(2)	9455.3(13)	4754.9(11)	58.7(3)
O(11)	750(2)	8186.8(17)	4507.5(12)	67.1(4)
O(12)	6472.6(15)	3830.4(11)	7728.9(8)	33.0(2)

Table S16. Anisotropic Displacement Parameters ($Å^2 \times 10^3$) for Compound **8**. The Anisotropic displacement factor exponent takes the form: $-2\pi^2[h^2a^{*2}U_{11}+2hka^*b^*U_{12}+\ldots]$.

Atom	U_{11}	U_{22}	U_{33}	U_{23}	U_{13}	U_{12}
O(1)	58.1(6)	49.4(6)	38.1(5)	-22.2(5)	15.1(4)	-16.5(5)
N(1)	32.7(5)	38.4(6)	43.9(6)	-22.2(5)	6.1(4)	-10.0(4)
C(1)	27.2(5)	29.3(5)	27.5(5)	-9.1(4)	6.0(4)	-8.2(4)
O(2)	55.4(7)	43.5(6)	74.2(8)	-27.8(6)	-1.1(6)	7.3(5)
N(2)	49.4(7)	40.9(6)	44.2(6)	-20.2(5)	-3.1(5)	-16.4(5)
C(2)	27.3(5)	37.3(6)	26.8(5)	-12.5(5)	4.4(4)	-12.7(5)
O(3)	35.7(5)	36.5(5)	32.1(5)	-8.9(4)	5.5(4)	-2.2(4)
N(3)	35.8(6)	35.1(6)	38.3(6)	-12.5(4)	-4.7(4)	-7.0(4)
C(3)	34.4(6)	34.7(6)	28.4(5)	-15.0(5)	6.4(4)	-15.6(5)
O(4)	33.6(4)	39.2(5)	24.5(4)	-7.2(3)	4.5(3)	-13.4(4)
C(4)	29.9(6)	29.4(5)	31.8(6)	-11.5(5)	3.3(4)	-9.3(4)
O(5)	45.4(5)	43.7(5)	36.9(5)	-23.4(4)	9.2(4)	-19.7(4)
C(5)	29.0(5)	29.4(5)	27.3(5)	-8.6(4)	-0.4(4)	-12.4(4)
O(6)	83.0(9)	90.0(10)	75.9(8)	-49.1(8)	29.3(7)	-62.2(8)
C(6)	31.8(6)	28.6(5)	25.9(5)	-10.1(4)	4.7(4)	-11.6(4)
O(7)	80.0(8)	60.4(7)	55.4(7)	-39.6(6)	2.2(6)	-20.5(6)
C(7)	32.7(6)	36.0(6)	30.6(6)	-8.8(5)	7.7(5)	-15.6(5)
O(8)	28.9(4)	39.4(5)	36.7(5)	-13.3(4)	10.1(3)	-12.7(4)
O(9)	35.5(5)	31.8(4)	38.2(5)	-7.1(4)	-6.6(4)	-14.2(4)
O(10)	61.4(7)	33.2(5)	68.5(7)	-1.1(5)	-16.9(6)	-16.8(5)
O(11)	50.8(7)	61.1(7)	80.9(9)	-19.7(6)	-30.9(6)	-12.1(6)
O(12)	38.4(5)	30.5(4)	33.3(4)	-10.1(3)	6.3(3)	-17.2(4)

Table S17. Bond Lengths for Compound **8**.

Atom	Atom	Length/Å	Atom	Atom	Length/Å
O(1)	N(1)	1.1977(15)	N(3)	O(10)	1.1779(15)
N(1)	O(2)	1.1944(15)	N(3)	O(11)	1.1931(15)
N(1)	O(3)	1.3986(14)	C(3)	C(4)	1.5270(16)
C(1)	C(2)	1.5247(15)	C(3)	O(5)	1.4345(14)

Atom	Atom	Length/Å	Atom	Atom	Length/Å
C(1)	O(3)	1.4418(14)	O(4)	C(7)	1.4013(15)
C(1)	C(6)	1.5298(16)	C(4)	C(5)	1.5314(16)
N(2)	O(5)	1.4458(14)	C(4)	O(8)	1.4353(14)
N(2)	O(6)	1.1909(18)	C(5)	C(6)	1.5255(15)
N(2)	O(7)	1.1849(16)	C(5)	O(9)	1.4477(13)
C(2)	C(3)	1.5248(16)	C(6)	O(12)	1.4320(13)
C(2)	O(4)	1.4370(13)	C(7)	O(8)	1.4108(15)
N(3)	O(9)	1.4047(14)	C(7)	O(12)	1.4049(15)

Table S18. Bond Angles for Compound **8**.

Atom	Atom	Atom	Angle/°	Atom	Atom	Atom	Angle/°
O(1)	N(1)	O(3)	118.39(10)	O(5)	C(3)	C(4)	107.74(9)
O(2)	N(1)	O(1)	129.04(12)	C(7)	O(4)	C(2)	111.50(8)
O(2)	N(1)	O(3)	112.57(11)	C(3)	C(4)	C(5)	106.76(9)
C(2)	C(1)	C(6)	107.77(9)	O(8)	C(4)	C(3)	110.07(9)
O(3)	C(1)	C(2)	105.18(9)	O(8)	C(4)	C(5)	109.25(9)
O(3)	C(1)	C(6)	111.34(9)	C(3)	O(5)	N(2)	114.11(9)
O(6)	N(2)	O(5)	117.39(11)	C(6)	C(5)	C(4)	107.92(9)
O(7)	N(2)	O(5)	111.40(12)	O(9)	C(5)	C(4)	111.18(9)
O(7)	N(2)	O(6)	131.21(13)	O(9)	C(5)	C(6)	105.39(9)
C(1)	C(2)	C(3)	108.04(9)	C(5)	C(6)	C(1)	107.16(9)
O(4)	C(2)	C(1)	109.30(9)	O(12)	C(6)	C(1)	109.34(9)
O(4)	C(2)	C(3)	109.13(9)	O(12)	C(6)	C(5)	109.95(9)
N(1)	O(3)	C(1)	114.04(9)	O(4)	C(7)	O(8)	111.05(10)
O(10)	N(3)	O(9)	119.16(10)	O(4)	C(7)	O(12)	111.68(9)
O(10)	N(3)	O(11)	128.99(12)	O(12)	C(7)	O(8)	110.34(9)
O(11)	N(3)	O(9)	111.85(11)	C(7)	O(8)	C(4)	111.28(9)
C(2)	C(3)	C(4)	107.86(9)	N(3)	O(9)	C(5)	114.40(9)
O(5)	C(3)	C(2)	110.25(9)	C(7)	O(12)	C(6)	111.32(8)

Table S19. Torsion Angles for Compound **8**.

A	B	C	D	Angle/°	A	B	C	D	Angle/°
O(1)	N(1)	O(3)	C(1)	1.76(15)	O(4)	C(7)	O(8)	C(4)	61.18(12)
C(1)	C(2)	C(3)	C(4)	64.12(11)	O(4)	C(7)	O(12)	C(6)	-61.36(12)
C(1)	C(2)	C(3)	O(5)	-178.49(9)	C(4)	C(3)	O(5)	N(2)	-136.66(10)
C(1)	C(2)	O(4)	C(7)	-58.39(12)	C(4)	C(5)	C(6)	C(1)	-64.99(11)
C(1)	C(6)	O(12)	C(7)	58.62(12)	C(4)	C(5)	C(6)	O(12)	53.75(11)
O(2)	N(1)	O(3)	C(1)	-178.63(11)	C(4)	C(5)	O(9)	N(3)	88.52(11)
C(2)	C(1)	O(3)	N(1)	-161.02(9)	O(5)	C(3)	C(4)	C(5)	176.69(9)

A	B	C	D	Angle/°	A	B	C	D	Angle/°
C(2)	C(1)	C(6)	C(5)	63.85(11)	O(5)	C(3)	C(4)	O(8)	-64.84(12)
C(2)	C(1)	C(6)	O(12)	-55.27(11)	C(5)	C(4)	O(8)	C(7)	59.27(12)
C(2)	C(3)	C(4)	C(5)	-64.31(11)	C(5)	C(6)	O(12)	C(7)	-58.77(12)
C(2)	C(3)	C(4)	O(8)	54.17(12)	O(6)	N(2)	O(5)	C(3)	-6.47(17)
C(2)	C(3)	O(5)	N(2)	105.88(11)	C(6)	C(1)	C(2)	C(3)	-63.65(11)
C(2)	O(4)	C(7)	O(8)	-62.43(12)	C(6)	C(1)	C(2)	O(4)	54.98(11)
C(2)	O(4)	C(7)	O(12)	61.19(12)	C(6)	C(1)	O(3)	N(1)	82.52(11)
O(3)	C(1)	C(2)	C(3)	177.48(9)	C(6)	C(5)	O(9)	N(3)	-154.80(9)
O(3)	C(1)	C(2)	O(4)	-63.89(11)	O(7)	N(2)	O(5)	C(3)	174.04(11)
O(3)	C(1)	C(6)	C(5)	178.71(9)	O(8)	C(4)	C(5)	C(6)	-53.87(11)
O(3)	C(1)	C(6)	O(12)	59.59(11)	O(8)	C(4)	C(5)	O(9)	61.24(12)
C(3)	C(2)	O(4)	C(7)	59.57(12)	O(8)	C(7)	O(12)	C(6)	62.66(12)
C(3)	C(4)	C(5)	C(6)	65.13(11)	O(9)	C(5)	C(6)	C(1)	176.14(8)
C(3)	C(4)	C(5)	O(9)	-179.75(9)	O(9)	C(5)	C(6)	O(12)	-65.12(11)
C(3)	C(4)	O(8)	C(7)	-57.66(12)	O(10)	N(3)	O(9)	C(5)	-0.52(17)
O(4)	C(2)	C(3)	C(4)	-54.62(11)	O(11)	N(3)	O(9)	C(5)	180.00(12)
O(4)	C(2)	C(3)	O(5)	62.77(11)	O(12)	C(7)	O(8)	C(4)	-63.20(12)

Table S20. Hydrogen Atom Coordinates (Å×10^4) and Isotropic Displacement Parameters (Å^2×10^3) for Compound **8**.

Atom	*x*	*y*	*z*	U(eq)
H(1)	10444.05	5660.44	6772.96	34
H(2)	10729.65	5349.25	8835.21	36
H(3)	8282.62	7995.85	7393.4	36
H(4)	4145.6	8393.66	7233.17	36
H(5)	6557.07	7491.88	5822.82	34
H(6)	7743.33	4488.89	6164.18	34
H(7)	5399.33	3608.69	9283.49	39

X-ray Crystal Structure and Data of Compound 12

CCDC 2425239

Single crystals of compound **12** suitable for X-ray diffraction analysis were obtained by slow recrystallization from a mixture of acetone and ethanol at room temperature. The result of X-ray diffraction indicates that compound **12** crystallizes in the monoclinic space group $P2_1/c$ and four moieties per unit cell (Z = 4). The crystal density was determined to be 1.887 g cm^{-3} at 301 K.

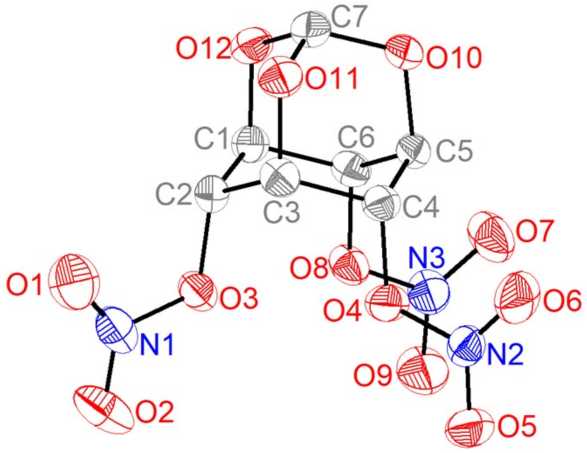

Figure S7. ORTEP diagram of Compound **12**. Color code: Carbon (grey), Oxygen (red), Nitrogen (blue).

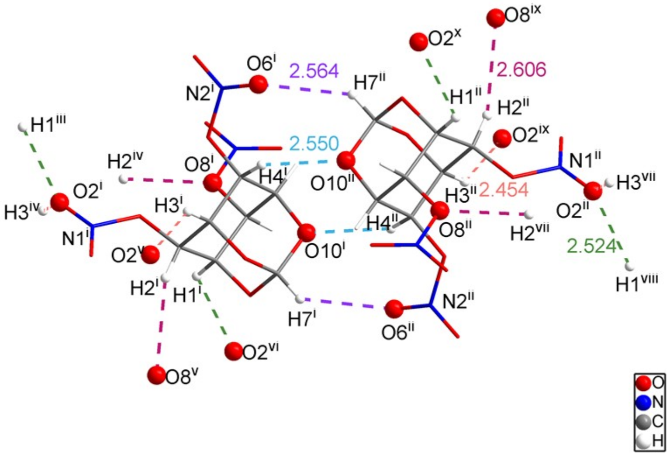

Figure S8. Hydrogen-bond network of compound **12**.

Table S21. Crystal data and structure refinement for Compound **12**.

Empirical formula	$C_7H_7N_3O_{12}$
Formula weight	325.16
Temperature/K	301.00
Crystal system	monoclinic
Space group	$P2_1/c$
a/Å	6.6576(2)
b/Å	18.5752(5)
c/Å	9.4128(2)
α/°	90
β/°	100.5850(10)
γ/°	90
Volume/Å^3	1144.24(5)

Z	4
ρ_{calc}g/cm^3	1.887
μ/mm^{-1}	0.187
F(000)	664.0
Crystal size/mm^3	0.1 × 0 × 0
Radiation	MoKα (λ = 0.71073)
2Θ range for data collection/°	4.386 to 54.988
Index ranges	-8 ≤ h ≤ 8, -24 ≤ k ≤ 22, -12 ≤ l ≤ 12
Reflections collected	30702
Independent reflections	2636 [R_{int} = 0.0576, R_{sigma} = 0.0237]
Data/restraints/parameters	2636/0/200
Goodness-of-fit on F^2	1.069
Final R indexes [I>=2σ (I)]	R_1 = 0.0335, wR_2 = 0.0848
Final R indexes [all data]	R_1 = 0.0449, wR_2 = 0.0976
Largest diff. peak/hole / e Å^{-3}	0.31/-0.20

Table S22. Fractional Atomic Coordinates (×10^4) and Equivalent Isotropic Displacement Parameters (Å^2×10^3) for Compound **12**. U_{eq} is defined as 1/3 of the trace of the orthogonalised U_{IJ} tensor.

**Atom**	***x***	***y***	***z***	**U(eq)**
O(1)	9897(2)	2021.2(9)	9585.6(17)	69.1(4)
O(2)	10636.6(19)	2030.7(7)	7441.5(16)	58.0(4)
O(3)	8102.9(16)	2696.1(6)	7810.7(11)	36.6(3)
O(4)	8500.6(17)	4239.8(6)	7764.3(13)	40.6(3)
O(5)	10504.5(19)	4876.4(7)	6692.9(14)	52.2(3)
O(6)	9176(2)	5395.9(6)	8357.9(15)	55.6(4)
O(7)	3354(2)	4427.6(7)	4824.1(14)	56.7(4)
O(8)	5277.1(17)	3527.7(6)	5915.7(10)	34.9(3)
O(9)	5435(3)	3851.5(8)	3728.0(14)	63.2(4)
O(10)	3712.1(16)	4292.7(5)	9165.2(11)	33.7(2)
O(11)	6104.6(18)	3616.0(6)	10707.5(11)	39.8(3)
O(12)	3440.4(17)	3050.1(6)	9172.0(12)	39.3(3)
N(1)	9682(2)	2196.1(7)	8349.4(17)	41.5(3)
N(2)	9478.3(18)	4906.0(7)	7601.6(14)	34.8(3)
N(3)	4611(2)	3980.6(7)	4722.7(14)	40.1(3)
C(1)	4620(2)	2959.8(7)	8043.8(16)	31.9(3)
C(2)	6822(2)	2870.0(7)	8838.7(15)	31.5(3)
C(3)	7458(2)	3556.5(8)	9678.7(15)	32.5(3)
C(4)	7143(2)	4246.6(7)	8790.9(15)	30.2(3)
C(5)	4915(2)	4292.0(7)	8036.1(15)	28.9(3)

Atom	*x*	*y*	*z*	U(eq)
C(6)	4203(2)	3632.6(7)	7097.8(15)	30.2(3)
C(7)	4066(3)	3665.5(8)	10010.1(17)	37.1(3)

Table S23. Anisotropic Displacement Parameters (Å^2×10^3) for Compound **12**. The Anisotropic displacement factor exponent takes the form: $-2\pi^2[h^2a^{*2}U_{11}+2hka^*b^*U_{12}+\ldots]$.

Atom	U_{11}	U_{22}	U_{33}	U_{23}	U_{13}	U_{12}
O(1)	58.2(9)	76.5(10)	72.1(10)	29.5(8)	10.5(7)	25.6(7)
O(2)	37.5(6)	62.0(8)	74.8(9)	-33.8(7)	10.8(6)	4.4(6)
O(3)	40.2(6)	34.3(5)	36.9(6)	0.1(4)	11.1(5)	10.0(4)
O(4)	44.2(6)	32.3(5)	51.3(7)	-10.2(5)	24.1(5)	-8.7(5)
O(5)	40.7(6)	60.5(8)	60.7(8)	17.6(6)	23.7(6)	6.7(6)
O(6)	74.0(9)	33.2(6)	63.5(8)	-7.6(6)	23.0(7)	-13.2(6)
O(7)	65.2(8)	61.3(8)	43.5(7)	11.5(6)	9.8(6)	31.9(7)
O(8)	42.2(6)	37.9(5)	24.6(5)	-0.8(4)	6.5(4)	11.4(4)
O(9)	93.7(11)	65.8(9)	37.2(7)	6.0(6)	30.2(7)	23.7(8)
O(10)	39.4(6)	31.8(5)	33.2(5)	-1.0(4)	15.5(4)	5.7(4)
O(11)	50.2(7)	44.6(6)	25.9(5)	0.3(4)	10.4(5)	5.6(5)
O(12)	41.3(6)	34.1(5)	47.6(6)	-1.3(5)	21.5(5)	-6.0(5)
N(1)	30.6(7)	32.4(6)	59.9(9)	-8.7(6)	4.3(6)	1.5(5)
N(2)	28.3(6)	35.0(7)	40.2(7)	7.9(6)	3.8(5)	0.9(5)
N(3)	50.5(8)	40.0(7)	29.6(7)	0.0(5)	7.0(6)	7.1(6)
C(1)	34.2(7)	26.0(7)	37.6(7)	-4.4(6)	11.7(6)	-3.9(5)
C(2)	37.3(8)	26.0(6)	32.8(7)	3.5(5)	10.8(6)	3.8(6)
C(3)	33.8(7)	36.1(7)	26.8(7)	-1.3(6)	3.3(6)	3.1(6)
C(4)	34.3(7)	26.9(6)	31.3(7)	-6.0(5)	10.9(6)	-3.6(5)
C(5)	35.0(7)	25.6(6)	28.0(7)	0.8(5)	10.6(6)	4.1(5)
C(6)	29.4(7)	33.7(7)	27.6(7)	-3.1(6)	5.5(5)	3.1(6)
C(7)	44.9(9)	35.5(8)	34.9(8)	0.7(6)	18.1(7)	1.5(6)

Table S24 Bond Lengths for Compound **12**.

Atom	Atom	Length/Å	Atom	Atom	Length/Å
O(1)	N(1)	1.191(2)	O(10)	C(5)	1.4436(16)
O(2)	N(1)	1.1950(19)	O(10)	C(7)	1.4058(18)
O(3)	N(1)	1.4246(17)	O(11)	C(3)	1.4431(17)
O(3)	C(2)	1.4393(17)	O(11)	C(7)	1.398(2)
O(4)	N(2)	1.4196(16)	O(12)	C(1)	1.4419(17)
O(4)	C(4)	1.4395(16)	O(12)	C(7)	1.4078(19)
O(5)	N(2)	1.1899(17)	C(1)	C(2)	1.527(2)
O(6)	N(2)	1.1950(18)	C(1)	C(6)	1.530(2)
O(7)	N(3)	1.1949(18)	C(2)	C(3)	1.519(2)

Atom	Atom	Length/Å	Atom	Atom	Length/Å
O(8)	N(3)	1.4079(16)	C(3)	C(4)	1.524(2)
O(8)	C(6)	1.4420(16)	C(4)	C(5)	1.524(2)
O(9)	N(3)	1.1930(17)	C(5)	C(6)	1.5335(19)

Table S25. Bond Angles for Compound **12**.

Atom	Atom	Atom	Angle/°	Atom	Atom	Atom	Angle/°
N(1)	O(3)	C(2)	113.38(11)	O(3)	C(2)	C(1)	109.21(11)
N(2)	O(4)	C(4)	114.94(10)	O(3)	C(2)	C(3)	113.27(12)
N(3)	O(8)	C(6)	113.99(10)	C(3)	C(2)	C(1)	108.31(11)
C(7)	O(10)	C(5)	110.96(10)	O(11)	C(3)	C(2)	105.35(12)
C(7)	O(11)	C(3)	111.22(11)	O(11)	C(3)	C(4)	105.28(11)
C(7)	O(12)	C(1)	111.23(11)	C(2)	C(3)	C(4)	115.07(12)
O(1)	N(1)	O(2)	130.10(15)	O(4)	C(4)	C(3)	108.70(11)
O(1)	N(1)	O(3)	118.29(13)	O(4)	C(4)	C(5)	111.39(12)
O(2)	N(1)	O(3)	111.58(14)	C(3)	C(4)	C(5)	109.05(11)
O(5)	N(2)	O(4)	111.87(12)	O(10)	C(5)	C(4)	106.31(11)
O(5)	N(2)	O(6)	130.18(14)	O(10)	C(5)	C(6)	105.67(11)
O(6)	N(2)	O(4)	117.95(12)	C(4)	C(5)	C(6)	113.30(11)
O(7)	N(3)	O(8)	118.30(12)	O(8)	C(6)	C(1)	106.03(11)
O(9)	N(3)	O(7)	129.18(14)	O(8)	C(6)	C(5)	114.06(11)
O(9)	N(3)	O(8)	112.53(13)	C(1)	C(6)	C(5)	108.26(11)
O(12)	C(1)	C(2)	104.80(12)	O(10)	C(7)	O(12)	110.75(12)
O(12)	C(1)	C(6)	105.58(11)	O(11)	C(7)	O(10)	111.57(12)
C(2)	C(1)	C(6)	115.43(12)	O(11)	C(7)	O(12)	111.81(12)

Table S26. Torsion Angles for Compound **12**.

A	B	C	D	Angle/°	A	B	C	D	Angle/°
O(3)	C(2)	C(3)	O(11)	-176.28(11)	C(2)	C(1)	C(6)	O(8)	68.18(15)
O(3)	C(2)	C(3)	C(4)	68.25(16)	C(2)	C(1)	C(6)	C(5)	-54.60(15)
O(4)	C(4)	C(5)	O(10)	179.83(10)	C(2)	C(3)	C(4)	O(4)	-66.43(16)
O(4)	C(4)	C(5)	C(6)	64.20(15)	C(2)	C(3)	C(4)	C(5)	55.18(16)
O(10)	C(5)	C(6)	O(8)	-178.53(10)	C(3)	O(11)	C(7)	O(10)	-63.36(15)
O(10)	C(5)	C(6)	C(1)	-60.77(14)	C(3)	O(11)	C(7)	O(12)	61.29(15)
O(11)	C(3)	C(4)	O(4)	178.06(11)	C(3)	C(4)	C(5)	O(10)	59.86(13)
O(11)	C(3)	C(4)	C(5)	-60.33(13)	C(3)	C(4)	C(5)	C(6)	-55.76(15)
O(12)	C(1)	C(2)	O(3)	173.70(10)	C(4)	O(4)	N(2)	O(5)	-176.74(12)
O(12)	C(1)	C(2)	C(3)	-62.52(13)	C(4)	O(4)	N(2)	O(6)	3.30(19)
O(12)	C(1)	C(6)	O(8)	-176.59(10)	C(4)	C(5)	C(6)	O(8)	-62.53(15)
O(12)	C(1)	C(6)	C(5)	60.62(14)	C(4)	C(5)	C(6)	C(1)	55.23(15)

A	B	C	D	Angle/°	A	B	C	D	Angle/°
N(1)	O(3)	C(2)	C(1)	-143.20(11)	C(5)	O(10)	C(7)	O(11)	61.98(15)
N(1)	O(3)	C(2)	C(3)	96.00(13)	C(5)	O(10)	C(7)	O(12)	-63.26(15)
N(2)	O(4)	C(4)	C(3)	-137.95(12)	C(6)	O(8)	N(3)	O(7)	4.61(19)
N(2)	O(4)	C(4)	C(5)	101.88(13)	C(6)	O(8)	N(3)	O(9)	-175.58(14)
N(3)	O(8)	C(6)	C(1)	162.36(11)	C(6)	C(1)	C(2)	O(3)	-70.63(15)
N(3)	O(8)	C(6)	C(5)	-78.60(15)	C(6)	C(1)	C(2)	C(3)	53.15(15)
C(1)	O(12)	C(7)	O(10)	63.34(16)	C(7)	O(10)	C(5)	C(4)	-59.30(14)
C(1)	O(12)	C(7)	O(11)	-61.76(15)	C(7)	O(10)	C(5)	C(6)	61.37(14)
C(1)	C(2)	C(3)	O(11)	62.41(14)	C(7)	O(11)	C(3)	C(2)	-60.77(14)
C(1)	C(2)	C(3)	C(4)	-53.06(16)	C(7)	O(11)	C(3)	C(4)	61.26(14)
C(2)	O(3)	N(1)	O(1)	-5.95(19)	C(7)	O(12)	C(1)	C(2)	61.01(14)
C(2)	O(3)	N(1)	O(2)	175.76(12)	C(7)	O(12)	C(1)	C(6)	-61.32(15)

Table S27. Hydrogen Atom Coordinates (Å×10^4) and Isotropic Displacement Parameters (Å^2×10^3) for Compound **12**.

Atom	*x*	*y*	*z*	U(eq)
H(1)	4173	2527.99	7474.71	38
H(2)	6873.12	2470.64	9524.79	38
H(3)	8879.64	3520.37	10182.86	39
H(4)	7465.97	4661.39	9435.83	36
H(5)	4675.48	4733.98	7461.41	35
H(6)	2734.11	3671.06	6719.97	36
H(7)	3223.56	3696.01	10759.27	44

X-ray Crystal Structure and Data of Compound 14

CCDC 2425247

Single crystals of compound **14** suitable for X-ray diffraction analysis were obtained by slow recrystallization from a mixture of acetone and ethanol at room temperature. The result of X-ray diffraction indicates that compound **14** crystallizes in the monoclinic space group $P2_1/n$ and four moieties per unit cell (Z = 4). The crystal density was determined to be 1.899 g cm^{-3} at 294.8 K.

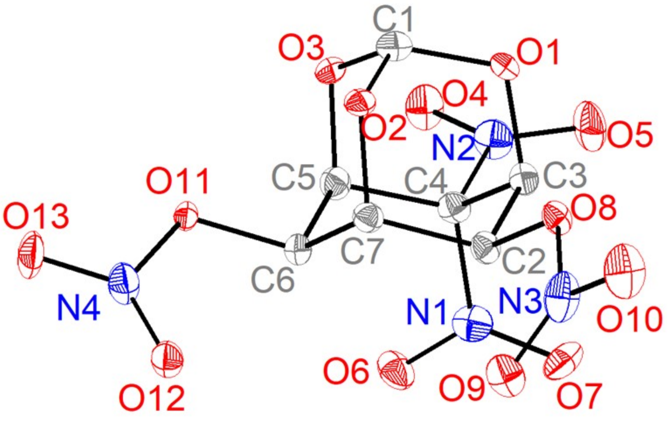

Figure S9. ORTEP diagram of Compound **14**. Color code: Carbon (grey), Oxygen (red), Nitrogen (blue).

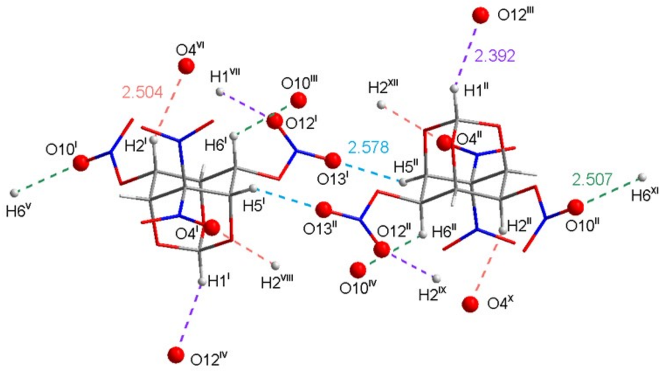

Figure S10. Hydrogen-bond network of compound **14**.

Table S28. Crystal data and structure refinement for Compound **14**.

Empirical formula	$C_7H_6N_4O_{13}$
Formula weight	354.145
Temperature/K	294.8
Crystal system	monoclinic
Space group	$P2_1/n$
a/Å	7.1632(9)
b/Å	12.3721(13)
c/Å	13.9868(18)
α/°	90
β/°	92.234(5)
γ/°	90
Volume/Å^3	1238.6(3)
Z	4
ρ_{calc}g/cm^3	1.899

μ/mm^{-1}	0.189
F(000)	720.7
Crystal size/mm^3	0.13 × 0.12 × 0.11
Radiation	Mo Kα (λ = 0.71073)
2Θ range for data collection/°	6.3 to 55.08
Index ranges	-9 ≤ h ≤ 9, -16 ≤ k ≤ 16, -18 ≤ l ≤ 11
Reflections collected	11484
Independent reflections	2824 [R_{int} = 0.0360, R_{sigma} = 0.0303]
Data/restraints/parameters	2824/0/217
Goodness-of-fit on F^2	1.054
Final R indexes [I>=2σ (I)]	R_1 = 0.0381, wR_2 = 0.0906
Final R indexes [all data]	R_1 = 0.0491, wR_2 = 0.0994
Largest diff. peak/hole / e Å^{-3}	0.37/-0.21

Table S29. Fractional Atomic Coordinates (×10^4) and Equivalent Isotropic Displacement Parameters (Å^2×10^3) for Compound **14**. U_{eq} is defined as 1/3 of of the trace of the orthogonalised U_{IJ} tensor.

**Atom**	***x***	***y***	***z***	**U(eq)**
O1	809(2)	4481.9(13)	864.1(11)	60.7(4)
N1	1389(2)	4515.2(11)	1682.6(11)	35.4(3)
C1	2645(2)	5481.5(12)	1967.4(11)	24.9(3)
O2	1032(2)	3880.5(11)	2307.3(11)	54.4(4)
N2	1244.8(18)	6387.4(11)	2123.4(11)	32.9(3)
C2	3831(2)	5219.4(12)	2875.5(11)	25.8(3)
O3	672.3(19)	6878.3(12)	1420.4(11)	53.2(4)
N3	7054(2)	6597.2(12)	4520.9(10)	34.7(3)
C3	5095(2)	6180.9(11)	3141.4(10)	22.9(3)
O4	783.9(19)	6538.9(13)	2936.0(10)	53.5(4)
N4	7004(2)	7951.8(13)	580.3(11)	39.8(4)
C4	6375(2)	6382.5(12)	2312.4(10)	24.0(3)
O5	7836(2)	6243.8(13)	5228.2(10)	60.9(4)
C5	5156(2)	6700.2(12)	1432.9(10)	23.2(3)
O6	6985(2)	7506.2(10)	4233.5(9)	45.0(3)
C6	3937(2)	5730.0(12)	1147.1(10)	25.3(3)
O7	6120.8(16)	5788.1(9)	3986.0(8)	30.6(3)
C7	6140(2)	4564.3(13)	1881.2(12)	33.1(4)
O8	4967.7(16)	4319.1(9)	2633.5(8)	32.7(3)
O9	5064.5(17)	4790.8(9)	1038.2(8)	33.0(3)
O10	7369.4(15)	5406.9(9)	2115.6(8)	31.5(3)
O11	6258.8(17)	6887.8(10)	611.7(8)	34.6(3)

Atom	*x*	*y*	*z*	U(eq)
O12	7861(2)	8093.0(15)	-124.2(11)	69.9(5)
O13	6700(2)	8547.2(11)	1225.9(10)	51.7(4)

Table S30. Anisotropic Displacement Parameters (Å^2×10^3) for Compound **14**. The Anisotropic displacement factor exponent takes the form: $-2^2[h^2a^{*2}U_{11}+2hka^*b^*U_{12}+\ldots]$.

Atom	U_{11}	U_{22}	U_{33}	U_{12}	U_{13}	U_{23}
O1	73.0(10)	64.4(10)	43.2(8)	-34.0(8)	-18.3(7)	-0.5(7)
N1	34.3(7)	32.4(7)	39.5(8)	-9.4(6)	-0.3(6)	-1.8(6)
C1	24.3(7)	23.4(7)	26.9(7)	-3.3(5)	0.8(6)	-0.2(6)
O2	63.5(9)	46.7(8)	52.8(9)	-28.8(7)	-1.6(7)	12.8(7)
N2	21.0(6)	35.4(7)	42.4(8)	-2.4(5)	2.5(6)	-3.9(6)
C2	29.3(7)	25.4(7)	22.9(7)	-1.7(6)	2.4(6)	2.4(6)
O3	44.0(8)	54.0(8)	61.9(9)	16.4(6)	4.4(7)	21.3(7)
N3	37.0(8)	40.9(8)	25.7(7)	-5.8(6)	-4.8(6)	-0.9(6)
C3	24.8(7)	24.9(7)	18.8(7)	2.3(5)	-0.6(5)	0.9(5)
O4	35.6(7)	80.4(10)	44.6(8)	13.4(7)	2.2(6)	-20.1(7)
N4	32.6(8)	53.2(9)	33.7(8)	-12.9(7)	1.5(6)	14.1(7)
C4	22.3(7)	26.1(7)	23.8(7)	-0.2(5)	3.5(6)	-1.1(6)
O5	76.2(11)	65.3(10)	38.7(8)	-12.0(8)	-31.5(8)	10.6(7)
C5	23.8(7)	27.8(7)	18.2(7)	-1.4(6)	5.0(5)	0.9(5)
O6	61.5(9)	34.8(7)	38.0(7)	-12.5(6)	-6.5(6)	2.7(5)
C6	27.6(7)	27.9(7)	20.3(7)	0.2(6)	0.4(6)	-0.5(6)
O7	39.5(6)	28.5(6)	23.1(5)	0.7(5)	-6.9(5)	2.1(4)
C7	35.9(9)	29.1(8)	34.1(9)	9.4(6)	0.2(7)	-4.6(6)
O8	40.4(6)	23.7(5)	33.8(6)	3.4(5)	-1.2(5)	2.4(4)
O9	40.4(6)	32.2(6)	26.5(6)	5.3(5)	0.9(5)	-8.4(5)
O10	24.9(5)	35.0(6)	34.6(6)	7.5(4)	2.3(5)	-4.1(5)
O11	39.9(6)	40.6(6)	24.2(6)	-6.9(5)	12.0(5)	1.3(5)
O12	72.2(11)	91.0(12)	48.3(9)	-21.8(9)	25.5(8)	24.2(8)
O13	58.7(9)	46.7(8)	49.9(8)	-24.2(7)	4.2(7)	-1.9(7)

Table S31. Bond Lengths for Compound **14**.

Atom	Atom	Length/Å	Atom	Atom	Length/Å
O1	N1	1.203(2)	C3	C4	1.526(2)
N1	C1	1.5392(19)	C3	O7	1.4506(17)
N1	O2	1.2096(19)	N4	O11	1.4218(19)
C1	N2	1.525(2)	N4	O12	1.194(2)

Atom	Atom	Length/Å	Atom	Atom	Length/Å
C1	C2	1.535(2)	N4	O13	1.192(2)
C1	C6	1.533(2)	C4	C5	1.532(2)
N2	O3	1.213(2)	C4	O10	1.4336(17)
N2	O4	1.2103(19)	C5	C6	1.528(2)
C2	C3	1.532(2)	C5	O11	1.4382(17)
C2	O8	1.4280(19)	C6	O9	1.4268(18)
N3	O5	1.2000(18)	C7	O8	1.405(2)
N3	O6	1.1947(19)	C7	O9	1.411(2)
N3	O7	1.4036(17)	C7	O10	1.396(2)

Table S32. Bond Angles for Compound **14**.

Atom	Atom	Atom	Angle/°	Atom	Atom	Atom	Angle/°
C1	N1	O1	116.71(14)	O12	N4	O11	111.51(16)
O2	N1	O1	126.17(15)	O13	N4	O11	117.97(13)
O2	N1	C1	117.09(14)	O13	N4	O12	130.52(18)
N2	C1	N1	103.12(11)	C5	C4	C3	108.14(11)
C2	C1	N1	110.43(12)	O10	C4	C3	109.03(12)
C2	C1	N2	112.50(12)	O10	C4	C5	109.51(12)
C6	C1	N1	109.02(12)	C6	C5	C4	108.09(12)
C6	C1	N2	112.28(12)	O11	C5	C4	111.65(12)
C6	C1	C2	109.30(12)	O11	C5	C6	104.12(11)
O3	N2	C1	117.07(14)	C5	C6	C1	108.49(11)
O4	N2	C1	116.95(14)	O9	C6	C1	106.11(12)
O4	N2	O3	125.97(15)	O9	C6	C5	110.34(12)
C3	C2	C1	109.86(12)	C3	O7	N3	114.26(11)
O8	C2	C1	105.72(12)	O9	C7	O8	110.25(13)
O8	C2	C3	109.05(12)	O10	C7	O8	112.06(13)
O6	N3	O5	129.22(15)	O10	C7	O9	111.52(13)
O7	N3	O5	112.14(14)	C7	O8	C2	111.84(11)
O7	N3	O6	118.64(13)	C7	O9	C6	111.43(11)
C4	C3	C2	108.01(11)	C7	O10	C4	111.11(11)
O7	C3	C2	102.32(11)	C5	O11	N4	113.12(12)
O7	C3	C4	111.90(12)				

Table S33. Torsion Angles for Compound **14**.

A	B	C	D	Angle/°	A	B	C	D	Angle/°
O1	N1	C1	N2	80.39(17)	C2	C3	C4	O10	-55.60(12)
O1	N1	C1	C2	-159.20(16)	C2	C3	O7	N3	-165.01(11)
O1	N1	C1	C6	-39.10(17)	C2	O8	C7	O9	-63.90(12)

A	B	C	D	Angle/°	A	B	C	D	Angle/°
N1	C1	N2	O3	-83.39(13)	C2	O8	C7	O10	60.96(12)
N1	C1	N2	O4	95.44(13)	N3	O7	C3	C4	79.60(12)
N1	C1	C2	C3	179.13(12)	C3	C4	C5	C6	-65.11(12)
N1	C1	C2	O8	61.60(12)	C3	C4	C5	O11	-179.04(11)
N1	C1	C6	C5	179.21(12)	C3	C4	O10	C7	59.07(13)
N1	C1	C6	O9	-62.24(12)	N4	O11	C5	C4	-84.27(12)
C1	C2	C3	C4	-60.66(13)	N4	O11	C5	C6	159.36(13)
C1	C2	C3	O7	-178.85(12)	C4	C5	C6	O9	-52.82(12)
C1	C2	O8	C7	60.62(13)	C4	O10	C7	O8	-61.45(12)
C1	C6	C5	C4	63.02(12)	C4	O10	C7	O9	62.70(12)
C1	C6	C5	O11	-178.14(12)	C5	C6	O9	C7	56.85(14)
C1	C6	O9	C7	-60.47(13)	C6	O9	C7	O8	63.62(12)
C2	C3	C4	C5	63.39(12)	C6	O9	C7	O10	-61.54(12)

Table S34. Hydrogen Atom Coordinates (Å×10^4) and Isotropic Displacement Parameters (Å^2×10^3) for Compound **14**.

Atom	*x*	*y*	*z*	U(eq)
H2	3033(2)	5035.8(12)	3405.0(11)	31.0(4)
H3	4361(2)	6825.5(11)	3284.5(10)	27.4(4)
H4	7258(2)	6964.5(12)	2477.1(10)	28.8(4)
H5	4385(2)	7332.3(12)	1568.6(10)	27.8(4)
H6	3205(2)	5881.5(12)	555.5(10)	30.4(4)
H7	6891(2)	3920.2(13)	1759.9(12)	39.7(4)

X-ray Crystal Structure and Data of Compound 15

CCDC 2425251

Single crystals of compound **15** suitable for X-ray diffraction analysis were obtained by slow recrystallization from a mixture of acetone and ethanol at room temperature. The result of X-ray diffraction indicates that compound **15** crystallizes in the monoclinic space group $P2_1/c$ and four moieties per unit cell (Z = 4). The crystal density was determined to be 1.884 g cm^{-3} at 296.15 K.

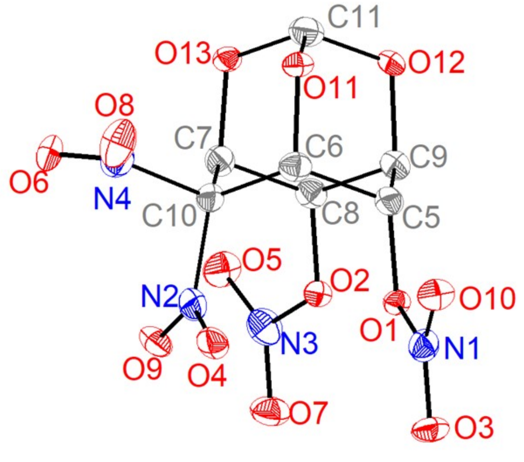

Figure S11. ORTEP diagram of Compound **15**. Color code: Carbon (grey), Oxygen (red), Nitrogen (blue).

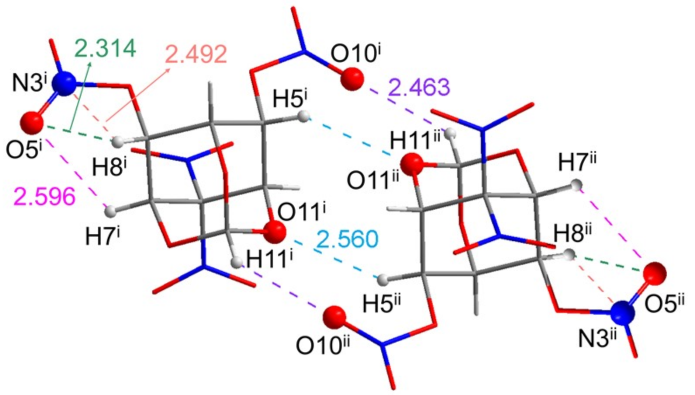

Figure S12. Hydrogen-bond network of compound **15**.

Table S35. Crystal data and structure refinement for Compound **15**.

Empirical formula	$C_7H_6N_4O_{13}$
Formula weight	354.16
Temperature/K	296.15
Crystal system	monoclinic
Space group	$P2_1/c$
a/Å	8.5994(4)
b/Å	7.2097(3)
c/Å	20.1577(9)
α/°	90
β/°	92.1670(10)
γ/°	90
Volume/Å^3	1248.87(10)
Z	4
ρ_{calc}g/cm^3	1.884
μ/mm^{-1}	0.187

F(000)	720.0
Crystal size/mm^3	0.14 × 0.13 × 0.12
Radiation	MoKα (λ = 0.71073)
2Θ range for data collection/°	4.74 to 50.136
Index ranges	-9 ≤ h ≤ 10, -8 ≤ k ≤ 8, -24 ≤ l ≤ 24
Reflections collected	26912
Independent reflections	2212 [R_{int} = 0.0505, R_{sigma} = 0.0282]
Data/restraints/parameters	2212/0/217
Goodness-of-fit on F^2	1.062
Final R indexes [I>=2σ (I)]	R_1 = 0.0408, wR_2 = 0.0879
Final R indexes [all data]	R_1 = 0.0628, wR_2 = 0.0982
Largest diff. peak/hole / e Å^{-3}	0.20/-0.23

Table S36. Fractional Atomic Coordinates (×10^4) and Equivalent Isotropic Displacement Parameters (Å^2×10^3) for Compound **15**. U_{eq} is defined as 1/3 of of the trace of the orthogonalised U_{IJ} tensor.

**Atom**	***x***	***y***	***z***	**U(eq)**
O1	5605.8(15)	3403.3(18)	3316.7(6)	32.7(3)
O2	2932.5(15)	3414.9(19)	2579.8(7)	34.7(4)
O13	957.5(15)	4132(2)	4148.8(7)	37.8(4)
O11	3252.6(16)	4202(2)	4777.9(7)	38.7(4)
O12	2754.2(17)	6479.9(19)	3989.5(7)	40.4(4)
O10	7551.9(17)	3093(2)	4082.0(8)	50.4(4)
O9	2739(2)	-174(2)	3065.7(8)	50.9(4)
O3	7765.3(18)	2023(2)	3088.6(8)	53.0(5)
O4	4810.7(18)	-242(2)	3707.0(9)	55.7(5)
O5	478(2)	3025(3)	2182.9(9)	61.1(5)
O6	806(2)	-79(2)	4357.4(9)	59.0(5)
N1	7121.2(19)	2790(2)	3523.7(10)	35.6(4)
N2	3533(2)	346(2)	3537.3(10)	37.7(5)
O7	2434(2)	1995(3)	1645.5(9)	64.5(5)
N3	1824(2)	2759(3)	2091.7(10)	43.7(5)
N4	2078(2)	533(3)	4508.2(10)	44.9(5)
O8	2837(2)	187(3)	5005.6(10)	76.1(6)
C5	4794(2)	4351(3)	3828.3(10)	31.1(5)
C6	4058(2)	3039(3)	4326.1(10)	32.5(5)
C7	1579(2)	2980(3)	3641.9(10)	32.1(5)
C8	2244(2)	4309(3)	3133.4(10)	31.3(5)
C9	3512(2)	5477(3)	3475.9(10)	33.6(5)
C10	2830(2)	1793(3)	3990.4(10)	30.4(5)
C11	2099(2)	5280(3)	4446.5(11)	38.6(5)

Table S37. Anisotropic Displacement Parameters (Å^2×10^3) for Compound **15**. The Anisotropic displacement factor exponent takes the form: $-2\pi^2[h^2a^{*2}U_{11}+2hka^*b^*U_{12}+\ldots]$.

Atom	U_{11}	U_{22}	U_{33}	U_{23}	U_{13}	U_{12}
O1	24.9(7)	37.6(8)	35.6(8)	-5.3(6)	0.2(6)	1.1(6)
O2	32.2(8)	36.8(8)	34.9(8)	-3.1(6)	-1.5(6)	1.8(6)
O13	28.6(8)	40.2(8)	44.9(9)	-6.4(7)	4.8(7)	3.7(7)
O11	36.7(8)	45.7(9)	33.9(8)	-7.3(7)	2.8(7)	4.7(7)
O12	44.0(9)	26.7(7)	50.9(9)	-7.6(7)	8.0(7)	2.2(7)
O10	38.4(9)	69.1(12)	42.9(10)	-7.7(8)	-7.8(8)	6.4(8)
O9	70.6(12)	35.2(9)	46.6(10)	-8.9(8)	-2.0(9)	-1.0(8)
O3	41.7(9)	64.7(11)	53.5(10)	-13.5(9)	11.3(8)	13.3(8)
O4	39.0(10)	37.5(9)	91.0(14)	-8.4(9)	7.5(9)	8.2(8)
O5	35.4(10)	86.1(13)	60.9(11)	-14.5(10)	-8.5(8)	-3.2(9)
O6	50.2(11)	56.2(11)	71.8(13)	5.6(9)	19.7(9)	-16.5(9)
N1	28.1(10)	34.6(10)	44.3(12)	-0.1(9)	3.5(9)	-1.2(8)
N2	41.0(12)	23.4(9)	49.3(12)	2.3(8)	10.1(10)	-1.5(8)
O7	69.4(12)	72.3(12)	51.8(11)	-25.0(10)	2.6(9)	2.5(10)
N3	45.6(13)	42.9(11)	42.0(12)	-4.3(9)	-7.2(10)	-2.6(9)
N4	46.7(13)	38.6(11)	50.2(13)	7.9(9)	13.5(10)	2.2(10)
O8	76.3(14)	88.6(15)	63.2(13)	44.0(11)	-2.7(11)	-6.5(11)
C5	28.5(11)	29.7(10)	35.1(12)	-6.6(9)	2.1(9)	-3.2(9)
C6	28.4(11)	34.5(11)	34.5(12)	-1.7(9)	0.6(9)	4.5(9)
C7	26.7(11)	30.6(10)	38.8(12)	-1.9(9)	-0.2(9)	0.0(9)
C8	29.0(11)	29.6(10)	35.2(12)	0.3(9)	-0.5(9)	5.3(9)
C9	35.8(12)	25.7(10)	39.6(12)	-1.7(9)	4.6(10)	1.0(9)
C10	30.9(11)	26.4(10)	34.2(11)	1.8(9)	6.5(9)	0.0(8)
C11	36.5(13)	37.6(12)	41.8(13)	-8.3(10)	3.2(10)	4.2(10)

Table S38. Bond Lengths for Compound **15**.

Atom	Atom	Length/Å	Atom	Atom	Length/Å
O1	N1	1.424(2)	O4	N2	1.215(2)
O1	C5	1.439(2)	O5	N3	1.195(2)
O2	N3	1.425(2)	O6	N4	1.208(2)
O2	C8	1.436(2)	N2	C10	1.527(3)
O13	C7	1.435(2)	O7	N3	1.193(2)
O13	C11	1.402(2)	N4	O8	1.202(3)
O11	C6	1.435(2)	N4	C10	1.544(3)
O11	C11	1.409(2)	C5	C6	1.533(3)
O12	C9	1.439(2)	C5	C9	1.523(3)
O12	C11	1.398(3)	C6	C10	1.525(3)

O10	N1	1.192(2)	C7	C8	1.530(3)
O9	N2	1.209(2)	C7	C10	1.525(3)
O3	N1	1.191(2)	C8	C9	1.522(3)

Table S39. Bond Angles for Compound **15**.

**Atom**	**Atom**	**Atom**	**Angle/°**	**Atom**	**Atom**	**Atom**	**Angle/°**
N1	O1	C5	113.62(14)	O11	C6	C10	106.42(15)
N3	O2	C8	113.66(15)	C10	C6	C5	111.65(16)
C11	O13	C7	111.90(15)	O13	C7	C8	105.86(15)
C11	O11	C6	111.69(15)	O13	C7	C10	105.77(16)
C11	O12	C9	111.60(15)	C10	C7	C8	112.65(16)
O10	N1	O1	117.92(17)	O2	C8	C7	114.51(16)
O3	N1	O1	111.99(17)	O2	C8	C9	106.95(15)
O3	N1	O10	130.09(18)	C9	C8	C7	108.78(16)
O9	N2	O4	126.23(19)	O12	C9	C5	105.78(16)
O9	N2	C10	117.27(18)	O12	C9	C8	105.56(15)
O4	N2	C10	116.38(19)	C8	C9	C5	114.24(17)
O5	N3	O2	117.86(19)	N2	C10	N4	100.80(14)
O7	N3	O2	111.86(18)	C6	C10	N2	112.64(16)
O7	N3	O5	130.3(2)	C6	C10	N4	110.32(17)
O6	N4	C10	116.31(19)	C7	C10	N2	113.35(17)
O8	N4	O6	126.3(2)	C7	C10	N4	109.61(16)
O8	N4	C10	117.27(19)	C7	C10	C6	109.78(16)
O1	C5	C6	113.56(15)	O13	C11	O11	110.24(17)
O1	C5	C9	106.31(15)	O12	C11	O13	112.12(17)
C9	C5	C6	108.92(16)	O12	C11	O11	111.08(16)
O11	C6	C5	106.05(15)				

Table S40. Torsion Angles for Compound **15**.

**A**	**B**	**C**	**D**	**Angle/°**	**A**	**B**	**C**	**D**	**Angle/°**
O1	C5	C6	O11	-177.62(14)	C5	O1	N1	O10	0.7(2)
O1	C5	C6	C10	-62.1(2)	C5	O1	N1	O3	-179.57(16)
O1	C5	C9	O12	-177.52(14)	C5	C6	C10	N2	69.9(2)
O1	C5	C9	C8	66.8(2)	C5	C6	C10	N4	-178.31(15)
O2	C8	C9	O12	174.98(14)	C5	C6	C10	C7	-57.4(2)
O2	C8	C9	C5	-69.2(2)	C6	O11	C11	O13	62.9(2)
O13	C7	C8	O2	179.89(14)	C6	O11	C11	O12	-62.0(2)
O13	C7	C8	C9	60.32(19)	C6	C5	C9	O12	59.76(19)
O13	C7	C10	N2	174.95(14)	C6	C5	C9	C8	-55.9(2)
O13	C7	C10	N4	63.20(19)	C7	O13	C11	O11	-63.7(2)

O13	C7	C10	C6	-58.12(19)	C7	O13	C11	O12	60.6(2)
O11	C6	C10	N2	-174.80(15)	C7	C8	C9	O12	-60.84(19)
O11	C6	C10	N4	-63.0(2)	C7	C8	C9	C5	54.9(2)
O11	C6	C10	C7	57.87(19)	C8	O2	N3	O5	0.8(2)
O9	N2	C10	N4	91.6(2)	C8	O2	N3	O7	-178.72(17)
O9	N2	C10	C6	-150.81(17)	C8	C7	C10	N2	-69.9(2)
O9	N2	C10	C7	-25.4(2)	C8	C7	C10	N4	178.39(16)
O4	N2	C10	N4	-84.7(2)	C8	C7	C10	C6	57.1(2)
O4	N2	C10	C6	32.9(2)	C9	O12	C11	O13	-61.2(2)
O4	N2	C10	C7	158.28(17)	C9	O12	C11	O11	62.6(2)
O6	N4	C10	N2	-83.0(2)	C9	C5	C6	O11	-59.37(19)
O6	N4	C10	C6	157.80(18)	C9	C5	C6	C10	56.1(2)
O6	N4	C10	C7	36.8(2)	C10	C7	C8	O2	64.8(2)
N1	O1	C5	C6	-79.90(19)	C10	C7	C8	C9	-54.8(2)
N1	O1	C5	C9	160.36(14)	C11	O13	C7	C8	-59.1(2)
N3	O2	C8	C7	78.3(2)	C11	O13	C7	C10	60.7(2)
N3	O2	C8	C9	-161.10(15)	C11	O11	C6	C5	59.6(2)
O8	N4	C10	N2	93.7(2)	C11	O11	C6	C10	-59.4(2)
O8	N4	C10	C6	-25.5(3)	C11	O12	C9	C5	-61.0(2)
O8	N4	C10	C7	-146.5(2)	C11	O12	C9	C8	60.5(2)

Table S41. Hydrogen Atom Coordinates (Å×10^4) and Isotropic Displacement Parameters (Å^2×10^3) for Compound **15**.

**Atom**	***x***	***y***	***z***	**U(eq)**
H5	5514.3	5193.66	4066.35	37
H6	4857.11	2297.96	4562.78	39
H7	763.23	2199.77	3435.26	39
H8	1411.18	5132.74	2966.5	38
H9	3958.08	6340.49	3159.54	40
H11	1596.49	6041.2	4779.69	46

X-ray Crystal Structure and Data of Compound 18

CCDC 2425245

Single crystals of compound **18** suitable for X-ray diffraction analysis were obtained by slow recrystallization from a mixture of acetone and ethanol at room temperature. The result of X-ray diffraction indicates that compound **18** crystallizes in the orthorhombic space group P 21 21 21 and four moieties per unit cell (Z = 4). The crystal density was determined to be 1.98 g cm^{-3} at 299 K.

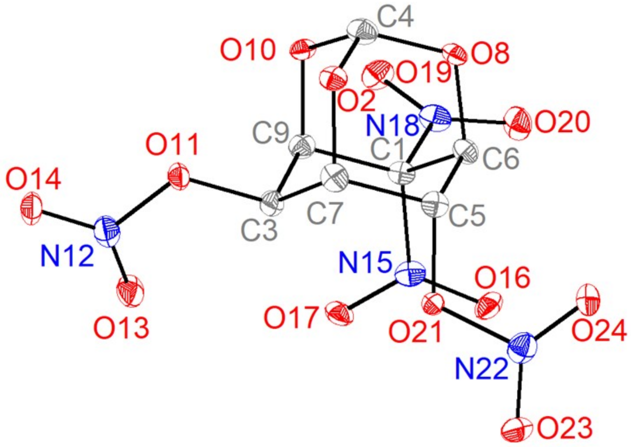

Figure S13. ORTEP diagram of Compound **18**. Color code: Carbon (grey), Oxygen (red), Nitrogen (blue).

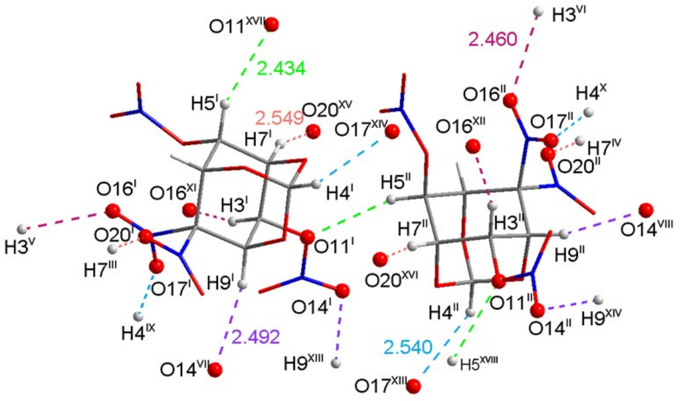

Figure S14. Hydrogen-bond network of compound **18**.

Table S42. Crystal data and structure refinement for Compound **18**.

Empirical formula	$C_7 H_6 N_4 O_{13}$	
Formula weight	354.16	
Temperature	299(2) K	
Wavelength	0.71073 Å	
Crystal system	Orthorhombic	
Space group	P 21 21 21	
Unit cell dimensions	a = 8.3535(4) Å	a= 90°.
	b = 9.1128(5) Å	b= 90°.
	c = 15.6114(8) Å	g = 90°.
Volume	1188.40(11) Å^3	
Z	4	
Density (calculated)	1.980 Mg/m^3	

Absorption coefficient	0.197 mm^{-1}
F(000)	720
Crystal size	0.130 x 0.130 x 0.120 mm^3
Theta range for data collection	2.588 to 29.926°.
Index ranges	-9<=h<=11, -9<=k<=12, -21<=l<=17
Reflections collected	5896
Independent reflections	2562 [R(int) = 0.0193]
Completeness to theta = 25.242°	100.0 %
Max. and min. transmission	0.977 and 0.975
Refinement method	Full-matrix least-squares on F^2
Data / restraints / parameters	2562 / 0 / 217
Goodness-of-fit on F^2	1.059
Final R indices [I>2sigma(I)]	R1 = 0.0267, wR2 = 0.0655
R indices (all data)	R1 = 0.0312, wR2 = 0.0668
Absolute structure parameter	-0.3(5)
Extinction coefficient	n/a
Largest diff. peak and hole	0.255 and -0.158 e.Å^{-3}

Table S43. Atomic coordinates (x 10^4) and equivalent isotropic displacement parameters (Å^{2}x 10^3) for Compound **18**. U(eq) is defined as one third of the trace of the orthogonalized Uij tensor.

	x	y	z	U(eq)
O(10)	6981(2)	3911(1)	7138(1)	29(1)
O(21)	8570(2)	8217(1)	6219(1)	28(1)
O(2)	8950(2)	5321(2)	7785(1)	31(1)
O(8)	6293(2)	6081(2)	7808(1)	29(1)
O(17)	6671(2)	6079(2)	4839(1)	37(1)
O(11)	9980(2)	3910(2)	6286(1)	33(1)
O(16)	5475(2)	7936(2)	5424(1)	40(1)
O(14)	11290(2)	2294(2)	5536(1)	45(1)
O(19)	4002(2)	4134(2)	6298(1)	44(1)
N(15)	6074(2)	6722(2)	5439(1)	25(1)
O(24)	7433(2)	10206(2)	6821(1)	46(1)
O(13)	9762(2)	3810(2)	4847(1)	49(1)
O(20)	3310(2)	6391(2)	6488(1)	47(1)
O(23)	8393(2)	10286(2)	5534(1)	49(1)
N(22)	8078(2)	9722(2)	6202(1)	32(1)
N(18)	4282(2)	5426(2)	6370(1)	28(1)
N(12)	10375(2)	3286(2)	5466(1)	31(1)

C(5)	8195(2)	7461(2)	7003(1)	24(1)
C(9)	7176(2)	4598(2)	6320(1)	23(1)
C(1)	6044(2)	5917(2)	6297(1)	22(1)
C(6)	6444(2)	6938(2)	7048(1)	23(1)
C(7)	9284(2)	6108(2)	7008(1)	26(1)
C(3)	8909(2)	5145(2)	6237(1)	25(1)
C(4)	7345(2)	4875(2)	7814(1)	30(1)

Table S44. Bond lengths [Å] and angles [°] for Compound **18**.

O(10)-C(4)	1.406(2)
O(10)-C(9)	1.432(2)
O(21)-N(22)	1.433(2)
O(21)-C(5)	1.440(2)
O(2)-C(4)	1.402(2)
O(2)-C(7)	1.436(2)
O(8)-C(4)	1.407(2)
O(8)-C(6)	1.425(2)
O(17)-N(15)	1.212(2)
O(11)-N(12)	1.438(2)
O(11)-C(3)	1.440(2)
O(16)-N(15)	1.214(2)
O(14)-N(12)	1.189(2)
O(19)-N(18)	1.206(2)
N(15)-C(1)	1.527(2)
O(24)-N(22)	1.191(2)
O(13)-N(12)	1.193(2)
O(20)-N(18)	1.211(2)
O(23)-N(22)	1.191(2)
N(18)-C(1)	1.542(2)
C(5)-C(7)	1.532(2)
C(5)-C(6)	1.539(2)
C(5)-H(5)	0.9800
C(9)-C(1)	1.530(2)
C(9)-C(3)	1.537(2)
C(9)-H(9)	0.9800
C(1)-C(6)	1.534(2)

C(6)-H(6)	0.9800
C(7)-C(3)	1.523(2)
C(7)-H(7)	0.9800
C(3)-H(3)	0.9800
C(4)-H(4)	0.9800
C(4)-O(10)-C(9)	111.86(13)
N(22)-O(21)-C(5)	114.33(13)
C(4)-O(2)-C(7)	110.99(13)
C(4)-O(8)-C(6)	112.24(13)
N(12)-O(11)-C(3)	113.84(13)
O(17)-N(15)-O(16)	126.52(16)
O(17)-N(15)-C(1)	116.85(15)
O(16)-N(15)-C(1)	116.62(15)
O(23)-N(22)-O(24)	130.59(18)
O(23)-N(22)-O(21)	111.48(16)
O(24)-N(22)-O(21)	117.93(16)
O(19)-N(18)-O(20)	126.35(17)
O(19)-N(18)-C(1)	117.48(16)
O(20)-N(18)-C(1)	116.16(15)
O(14)-N(12)-O(13)	130.85(18)
O(14)-N(12)-O(11)	111.52(16)
O(13)-N(12)-O(11)	117.63(15)
O(21)-C(5)-C(7)	105.07(14)
O(21)-C(5)-C(6)	113.17(14)
C(7)-C(5)-C(6)	108.37(14)
O(21)-C(5)-H(5)	110.0
C(7)-C(5)-H(5)	110.0
C(6)-C(5)-H(5)	110.0
O(10)-C(9)-C(1)	107.11(14)
O(10)-C(9)-C(3)	108.89(13)
C(1)-C(9)-C(3)	108.95(13)
O(10)-C(9)-H(9)	110.6
C(1)-C(9)-H(9)	110.6
C(3)-C(9)-H(9)	110.6
N(15)-C(1)-C(9)	112.83(14)
N(15)-C(1)-C(6)	112.08(14)
C(9)-C(1)-C(6)	108.90(14)

N(15)-C(1)-N(18)	102.71(13)
C(9)-C(1)-N(18)	111.08(13)
C(6)-C(1)-N(18)	109.10(14)
O(8)-C(6)-C(1)	106.54(13)
O(8)-C(6)-C(5)	106.94(14)
C(1)-C(6)-C(5)	111.10(14)
O(8)-C(6)-H(6)	110.7
C(1)-C(6)-H(6)	110.7
C(5)-C(6)-H(6)	110.7
O(2)-C(7)-C(3)	109.87(14)
O(2)-C(7)-C(5)	106.91(14)
C(3)-C(7)-C(5)	109.73(14)
O(2)-C(7)-H(7)	110.1
C(3)-C(7)-H(7)	110.1
C(5)-C(7)-H(7)	110.1
O(11)-C(3)-C(7)	106.34(14)
O(11)-C(3)-C(9)	109.06(14)
C(7)-C(3)-C(9)	108.31(15)
O(11)-C(3)-H(3)	111.0
C(7)-C(3)-H(3)	111.0
C(9)-C(3)-H(3)	111.0
O(2)-C(4)-O(10)	111.32(16)
O(2)-C(4)-O(8)	111.73(15)
O(10)-C(4)-O(8)	110.35(15)
O(2)-C(4)-H(4)	107.8
O(10)-C(4)-H(4)	107.8
O(8)-C(4)-H(4)	107.8

Symmetry transformations used to generate equivalent atoms:

Table S45. Anisotropic Displacement Parameters (Å^2×10^3) for Compound **18**. The Anisotropic displacement factor exponent takes the form: $-2\pi^2[h^2a^{*2}U_{11}+2hka^*b^*U_{12}+\ldots]$.

	U^{11}	U^{22}	U^{33}	U^{23}	U^{13}	U^{12}
O(10)	37(1)	27(1)	23(1)	5(1)	-2(1)	-3(1)
O(21)	31(1)	24(1)	29(1)	-1(1)	4(1)	-1(1)
O(2)	29(1)	41(1)	24(1)	4(1)	-7(1)	1(1)
O(8)	30(1)	38(1)	20(1)	1(1)	4(1)	0(1)
O(17)	41(1)	49(1)	21(1)	2(1)	2(1)	4(1)
O(11)	35(1)	37(1)	27(1)	-2(1)	0(1)	14(1)

O(16)	49(1)	32(1)	38(1)	8(1)	-9(1)	7(1)
O(14)	43(1)	36(1)	57(1)	-7(1)	6(1)	14(1)
O(19)	38(1)	37(1)	57(1)	2(1)	-3(1)	-14(1)
N(15)	24(1)	30(1)	23(1)	4(1)	-5(1)	-4(1)
O(24)	52(1)	37(1)	50(1)	-7(1)	9(1)	10(1)
O(13)	65(1)	52(1)	30(1)	-8(1)	-3(1)	16(1)
O(20)	24(1)	50(1)	68(1)	-1(1)	6(1)	2(1)
O(23)	54(1)	43(1)	51(1)	17(1)	9(1)	1(1)
N(22)	28(1)	28(1)	42(1)	1(1)	0(1)	-1(1)
N(18)	22(1)	35(1)	26(1)	2(1)	-1(1)	-6(1)
N(12)	29(1)	28(1)	34(1)	-6(1)	4(1)	1(1)
C(5)	25(1)	25(1)	22(1)	-3(1)	-3(1)	-3(1)
C(9)	27(1)	24(1)	20(1)	1(1)	-2(1)	0(1)
C(1)	20(1)	26(1)	19(1)	2(1)	-1(1)	-3(1)
C(6)	23(1)	26(1)	18(1)	-3(1)	0(1)	0(1)
C(7)	22(1)	32(1)	26(1)	-2(1)	-3(1)	0(1)
C(3)	25(1)	26(1)	24(1)	2(1)	1(1)	6(1)
C(4)	32(1)	37(1)	22(1)	4(1)	-1(1)	0(1)

Table S46. Hydrogen Atom Coordinates (Å×10^4) and Isotropic Displacement Parameters (Å^2×10^3) for Compound **18**.

	x	y	z	U(eq)
H(5)	8438	8089	7496	29
H(9)	6920	3908	5858	28
H(6)	5711	7777	7062	27
H(7)	10409	6411	6996	32
H(3)	9062	5687	5701	30
H(4)	7181	4343	8353	36

Table S47. Torsion Angles for Compound **18**.

C(5)-O(21)-N(22)-O(23)	-178.85(16)
C(5)-O(21)-N(22)-O(24)	0.6(2)
C(3)-O(11)-N(12)-O(14)	178.29(16)
C(3)-O(11)-N(12)-O(13)	-2.0(2)
N(22)-O(21)-C(5)-C(7)	-161.40(13)
N(22)-O(21)-C(5)-C(6)	80.55(17)
C(4)-O(10)-C(9)-C(1)	-59.57(17)
C(4)-O(10)-C(9)-C(3)	58.11(18)

O(17)-N(15)-C(1)-C(9)	15.7(2)
O(16)-N(15)-C(1)-C(9)	-165.30(15)
O(17)-N(15)-C(1)-C(6)	139.04(16)
O(16)-N(15)-C(1)-C(6)	-41.9(2)
O(17)-N(15)-C(1)-N(18)	-104.00(16)
O(16)-N(15)-C(1)-N(18)	75.02(18)
O(10)-C(9)-C(1)-N(15)	-177.56(12)
C(3)-C(9)-C(1)-N(15)	64.80(18)
O(10)-C(9)-C(1)-C(6)	57.33(16)
C(3)-C(9)-C(1)-C(6)	-60.31(17)
O(10)-C(9)-C(1)-N(18)	-62.85(17)
C(3)-C(9)-C(1)-N(18)	179.52(14)
O(19)-N(18)-C(1)-N(15)	109.49(18)
O(20)-N(18)-C(1)-N(15)	-69.87(19)
O(19)-N(18)-C(1)-C(9)	-11.4(2)
O(20)-N(18)-C(1)-C(9)	169.25(16)
O(19)-N(18)-C(1)-C(6)	-131.44(18)
O(20)-N(18)-C(1)-C(6)	49.2(2)
C(4)-O(8)-C(6)-C(1)	60.29(17)
C(4)-O(8)-C(6)-C(5)	-58.59(17)
N(15)-C(1)-C(6)-O(8)	176.97(13)
C(9)-C(1)-C(6)-O(8)	-57.48(16)
N(18)-C(1)-C(6)-O(8)	63.91(16)
N(15)-C(1)-C(6)-C(5)	-66.90(18)
C(9)-C(1)-C(6)-C(5)	58.65(18)
N(18)-C(1)-C(6)-C(5)	-179.96(14)
O(21)-C(5)-C(6)-O(8)	173.88(13)
C(7)-C(5)-C(6)-O(8)	57.77(17)
O(21)-C(5)-C(6)-C(1)	58.00(19)
C(7)-C(5)-C(6)-C(1)	-58.11(18)
C(4)-O(2)-C(7)-C(3)	-58.42(19)
C(4)-O(2)-C(7)-C(5)	60.60(18)
O(21)-C(5)-C(7)-O(2)	179.85(13)
C(6)-C(5)-C(7)-O(2)	-58.90(17)
O(21)-C(5)-C(7)-C(3)	-61.04(17)
C(6)-C(5)-C(7)-C(3)	60.21(19)
N(12)-O(11)-C(3)-C(7)	-151.38(14)
N(12)-O(11)-C(3)-C(9)	92.03(17)

O(2)-C(7)-C(3)-O(11)	-62.95(17)
C(5)-C(7)-C(3)-O(11)	179.78(14)
O(2)-C(7)-C(3)-C(9)	54.14(17)
C(5)-C(7)-C(3)-C(9)	-63.13(17)
O(10)-C(9)-C(3)-O(11)	61.69(18)
C(1)-C(9)-C(3)-O(11)	178.19(14)
O(10)-C(9)-C(3)-C(7)	-53.64(18)
C(1)-C(9)-C(3)-C(7)	62.86(17)
C(7)-O(2)-C(4)-O(10)	61.89(19)
C(7)-O(2)-C(4)-O(8)	-61.98(19)
C(9)-O(10)-C(4)-O(2)	-62.47(18)
C(9)-O(10)-C(4)-O(8)	62.18(18)
C(6)-O(8)-C(4)-O(2)	61.46(19)
C(6)-O(8)-C(4)-O(10)	-62.97(18)

Symmetry transformations used to generate equivalent atoms:

4 Identification Spectra of Compounds

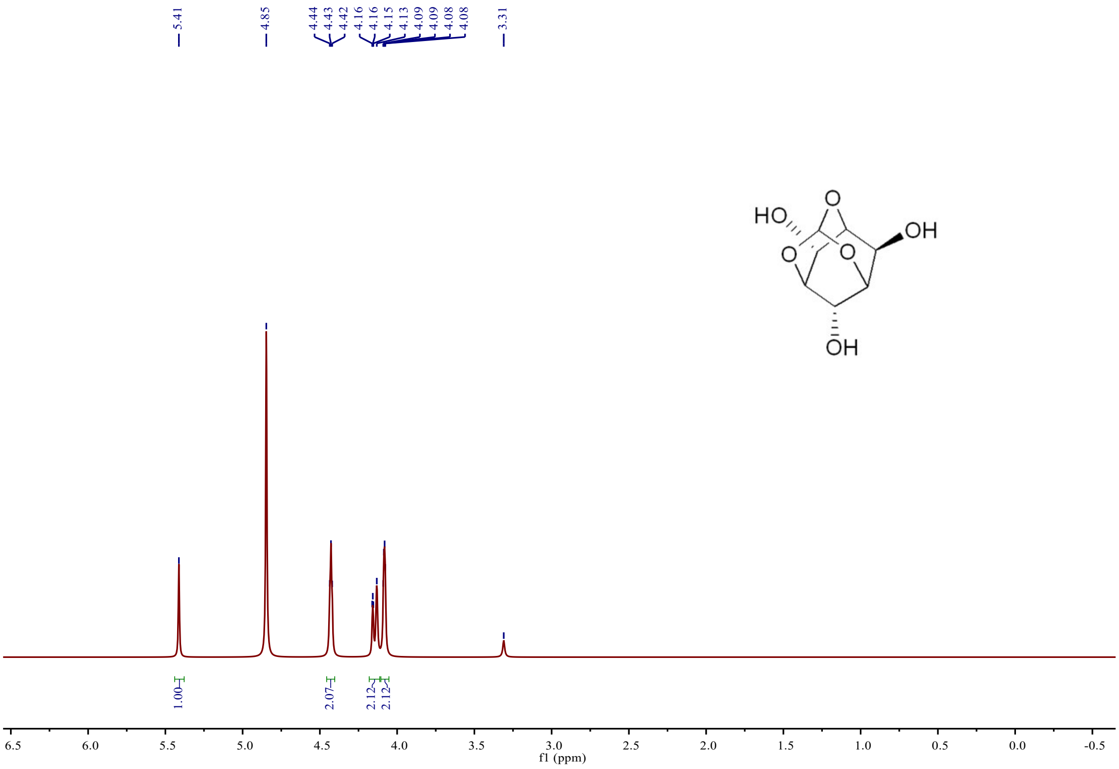

^{1}H NMR spectrum of compound **1** (Methanol-d_4, 500 MHz)

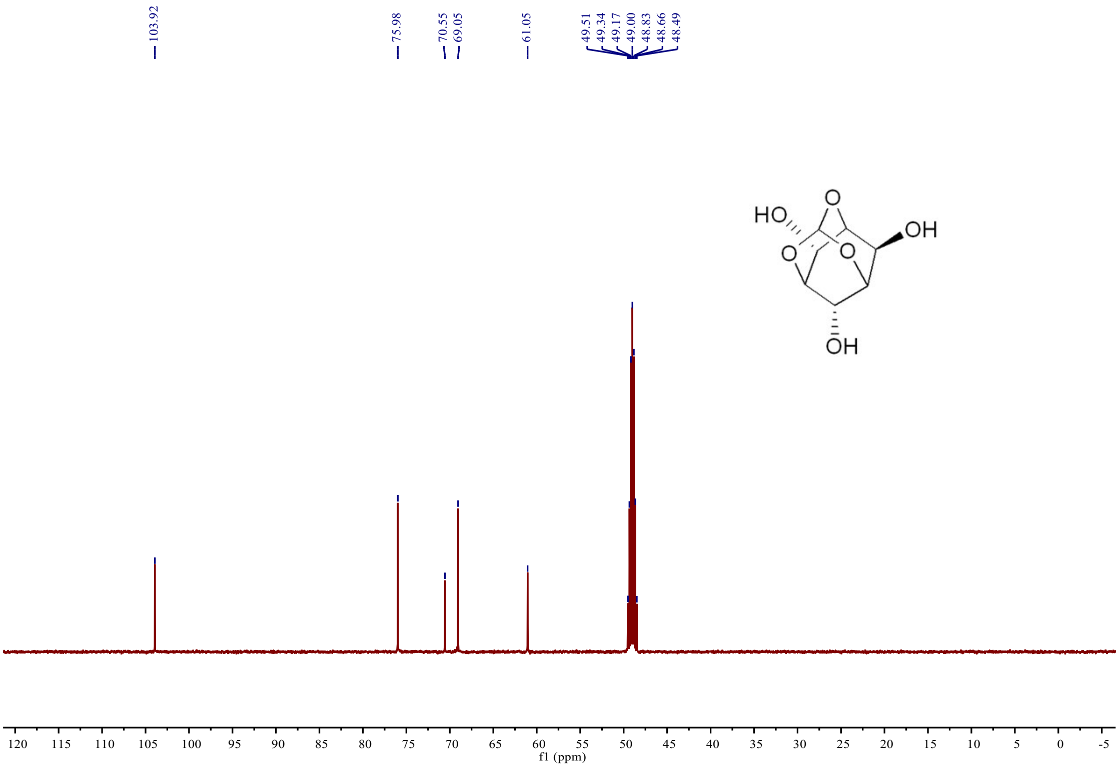

^{13}C NMR spectrum of compound **1** (Methanol-d_4, 126 MHz)

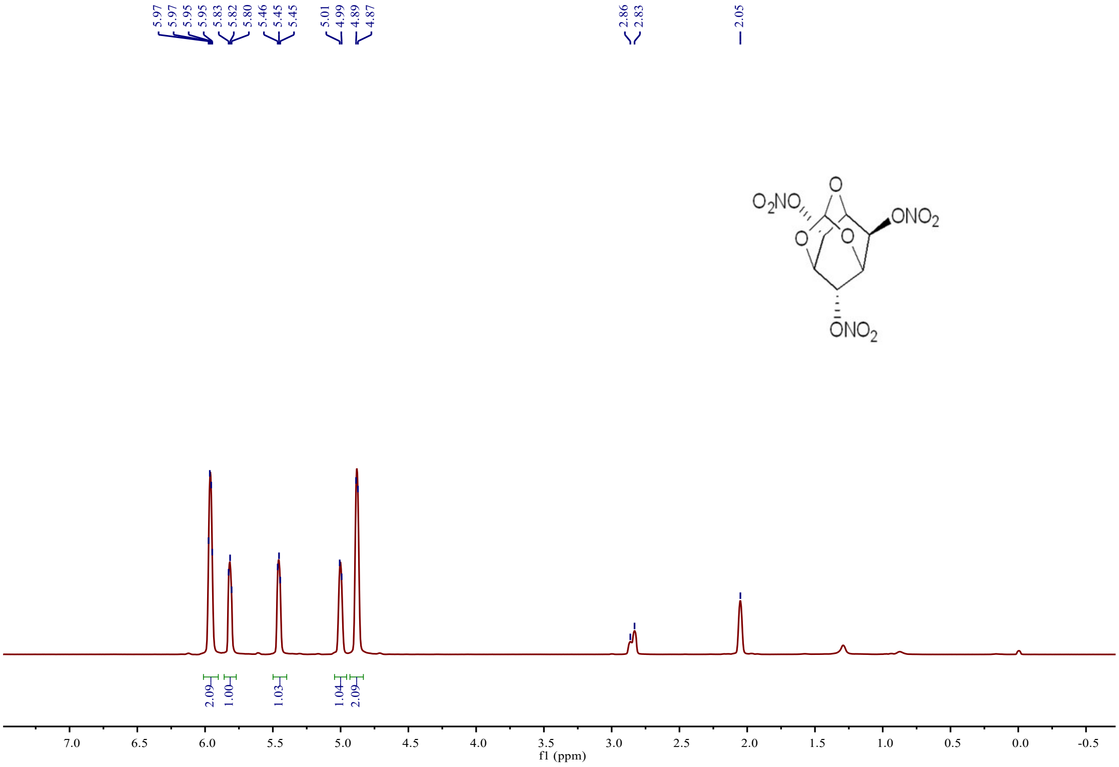

^{1}H NMR spectrum of compound **2** (Acetone-d_6, 500 MHz)

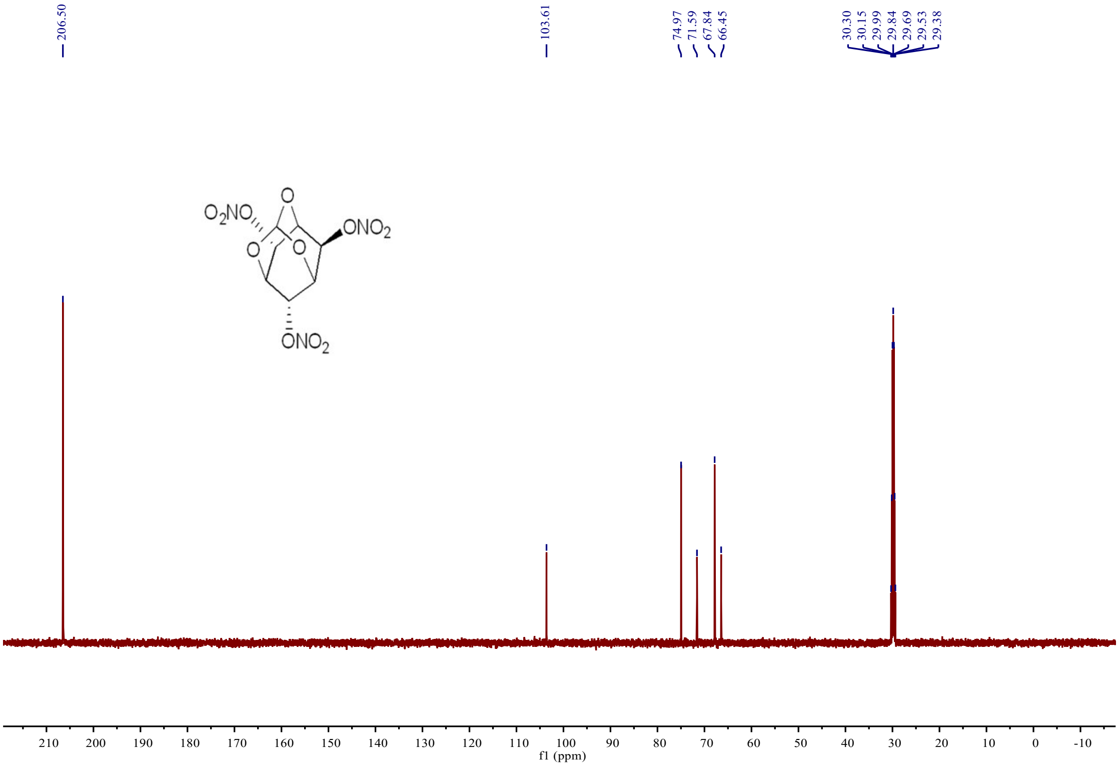

^{13}C NMR spectrum of compound **2** (Acetone-d_6, 126 MHz)

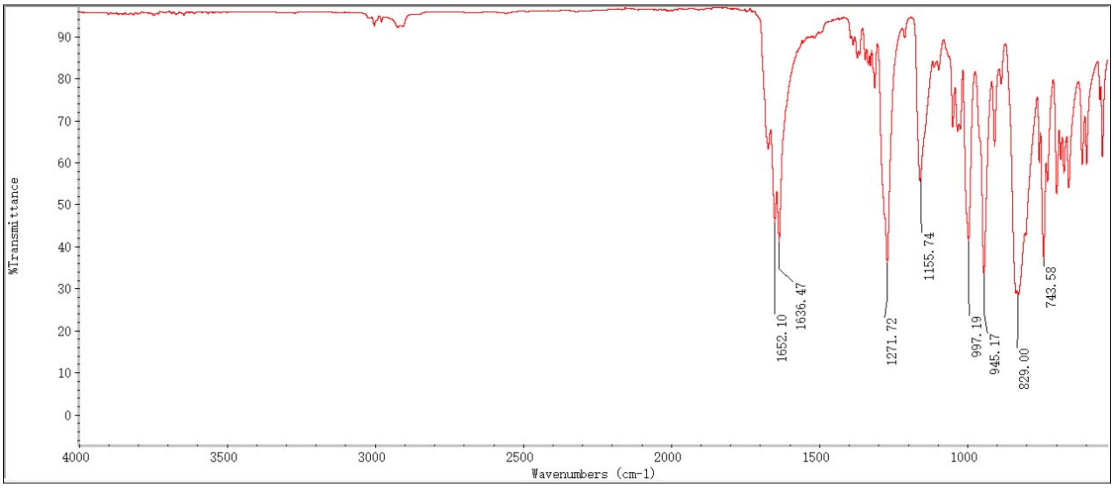

IR spectrum of compound **2**

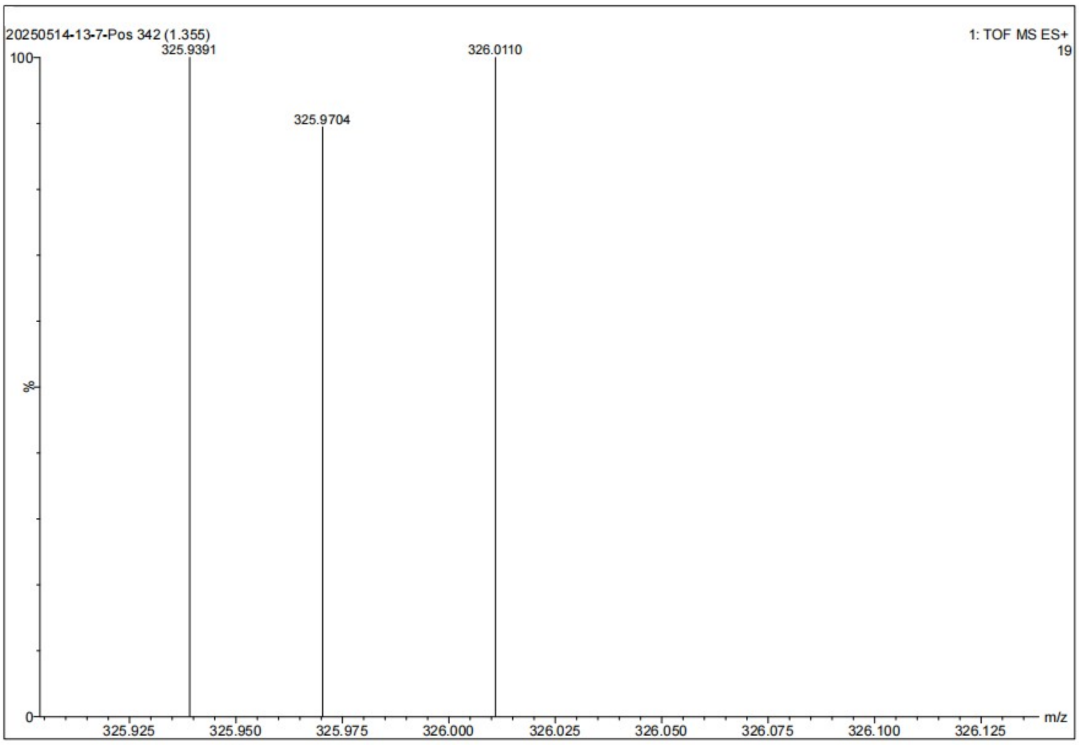

HMRS of compound **2**

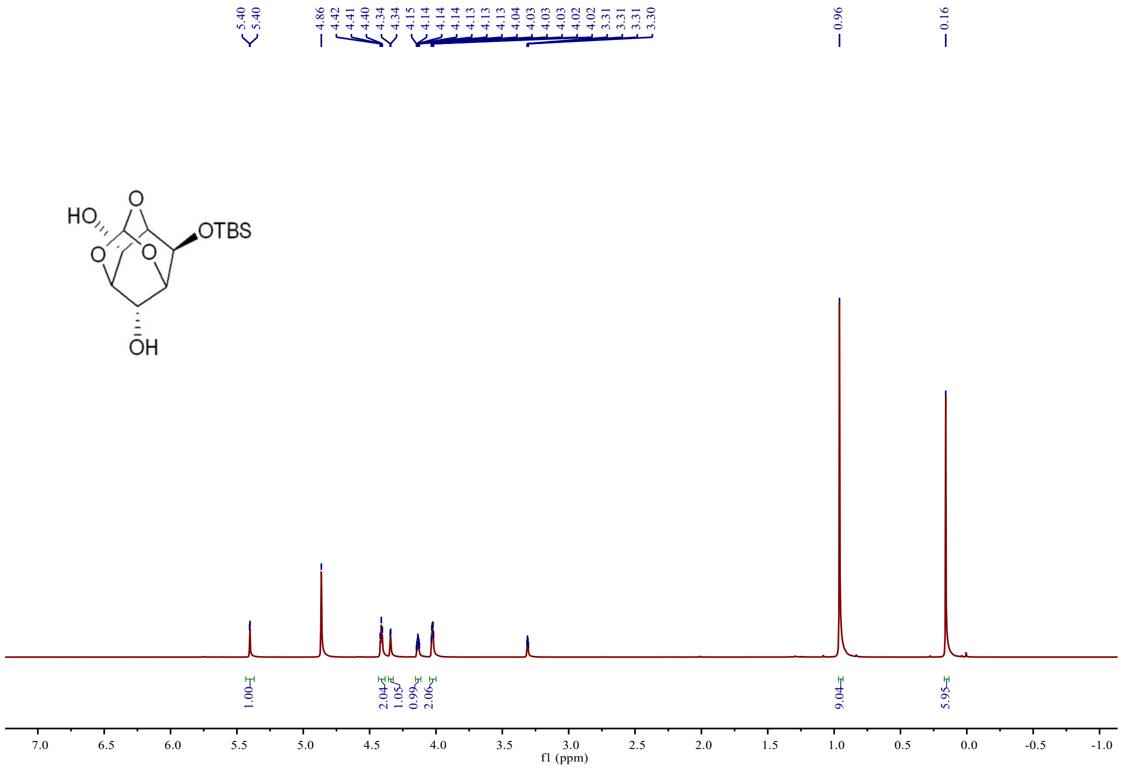

^{1}H NMR spectrum of compound **3** (Methanol-d_4, 500 MHz)

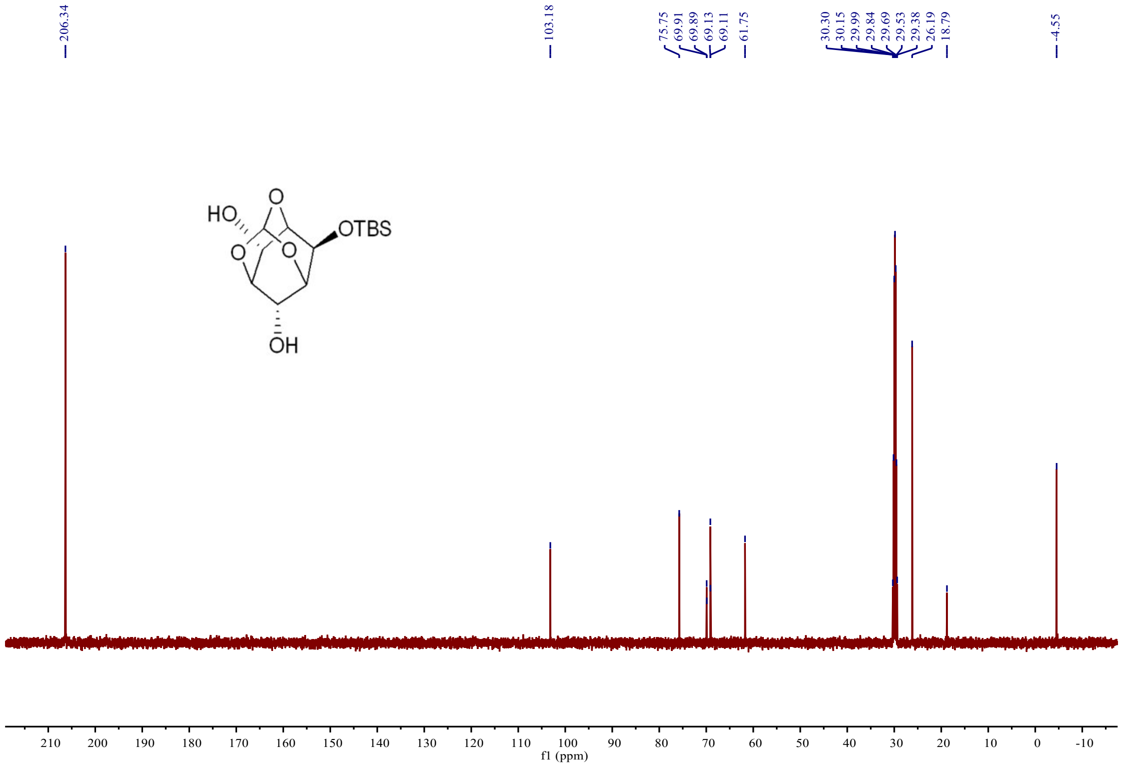

^{13}C NMR spectrum of compound **3** (Acetone-d_6, 126 MHz)

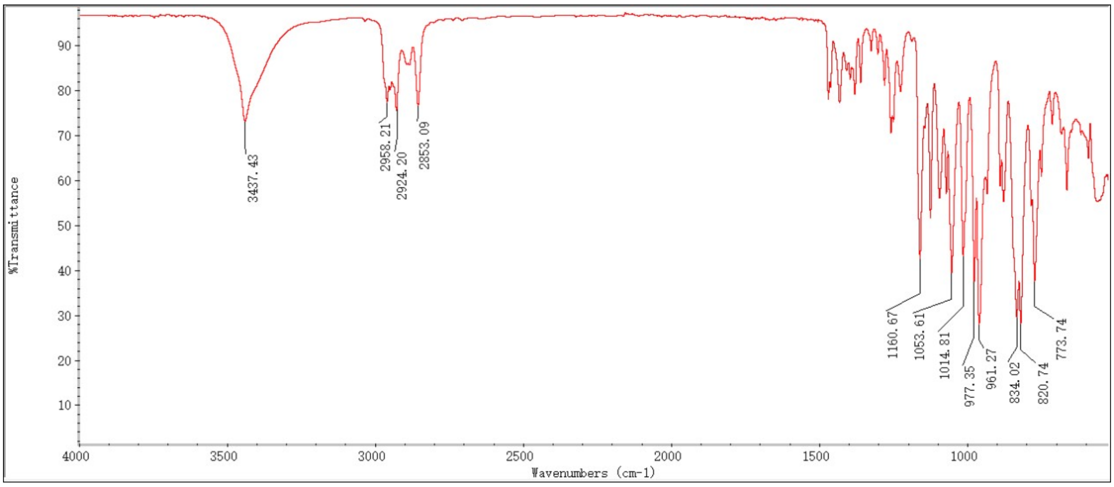

IR spectrum of compound **3**

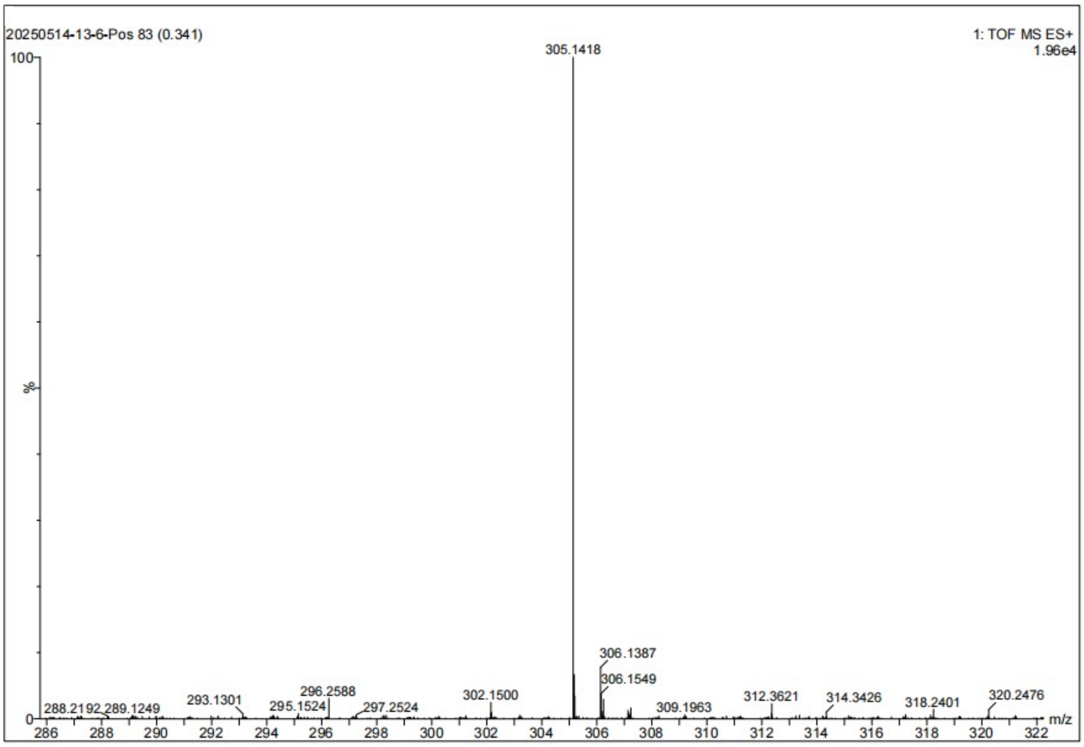

HMRS of compound **3**

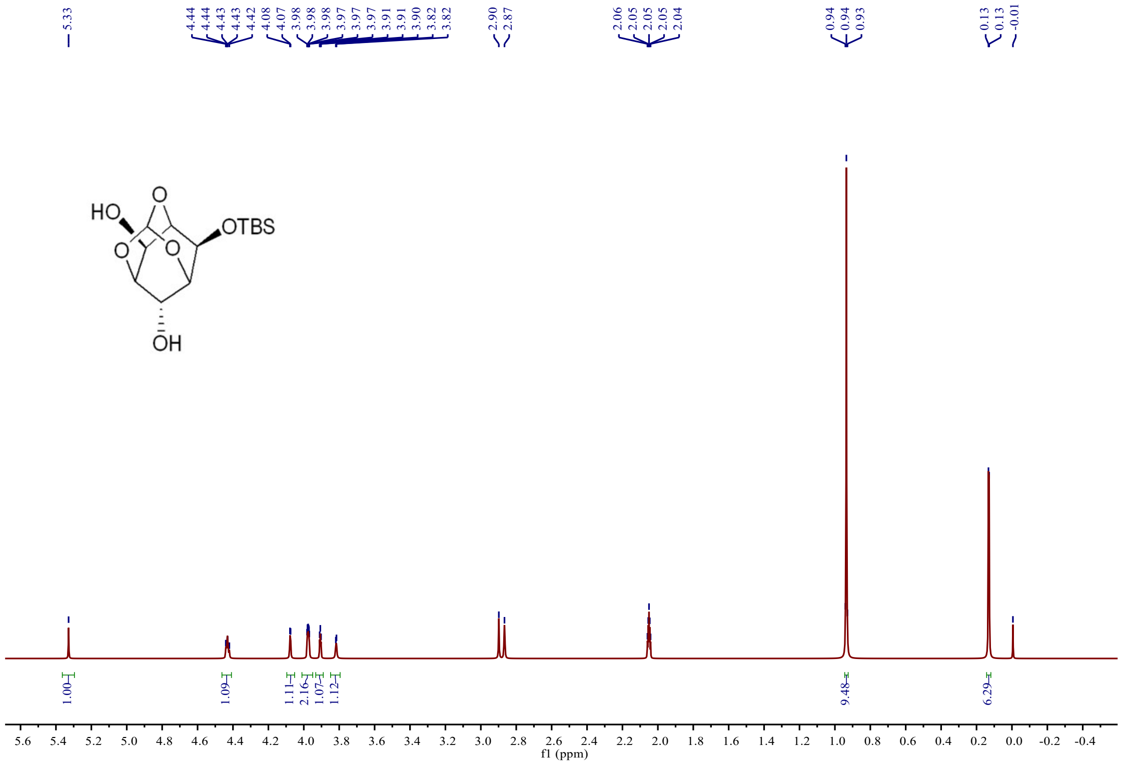

^{1}H NMR spectrum of compound **4** (Acetone-d_6, 500 MHz)

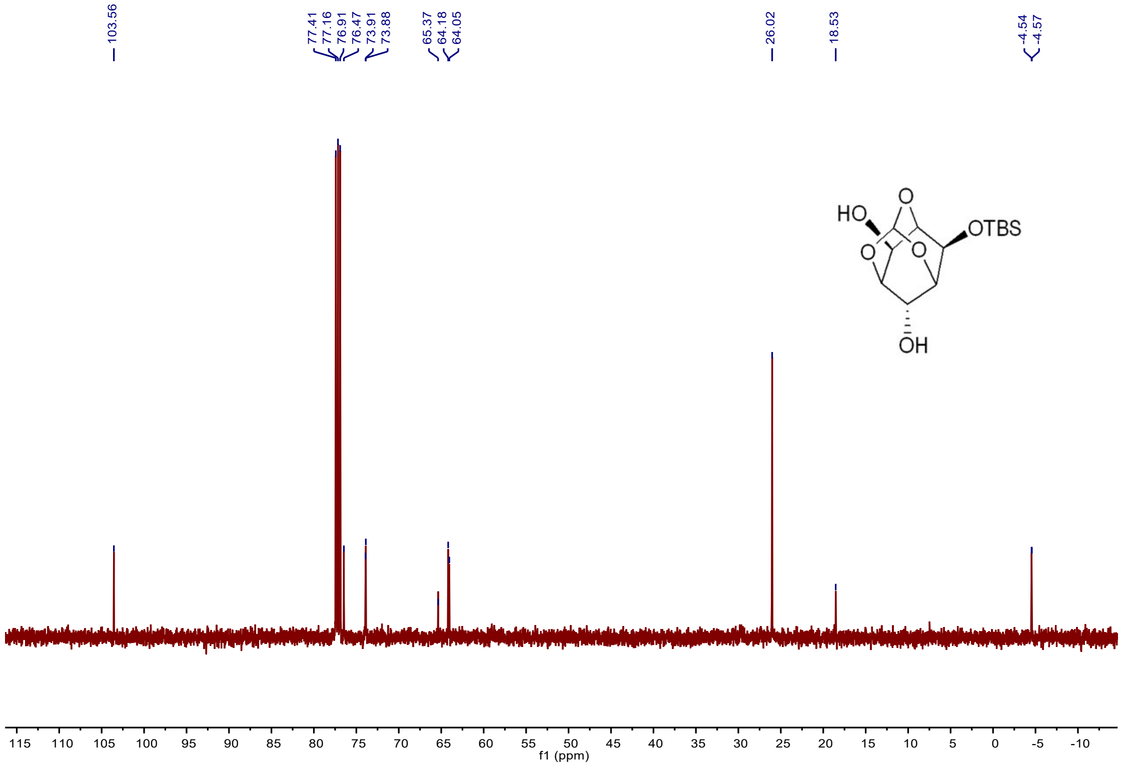

^{13}C NMR spectrum of compound **4** (Chloroform-*d*, 126 MHz)

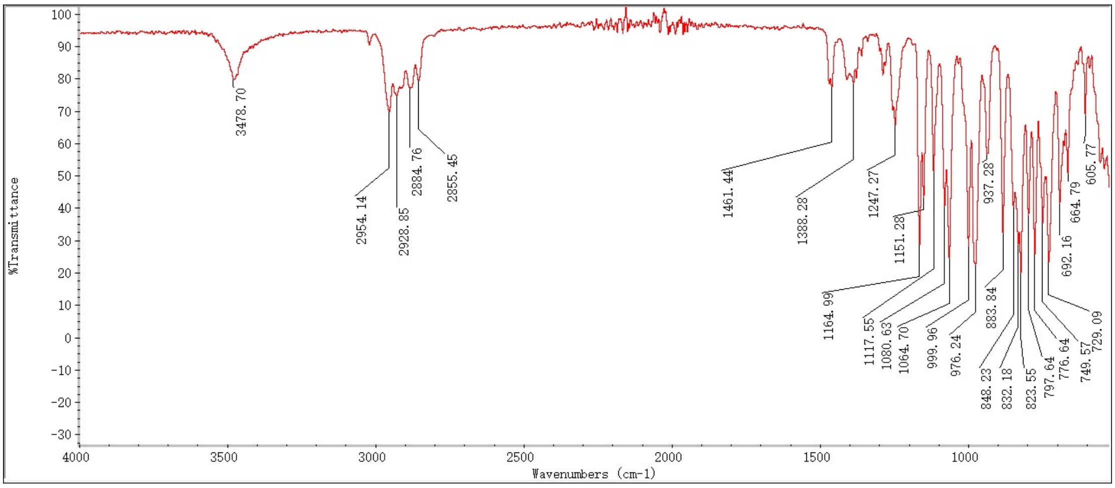

IR spectrum of compound **4**

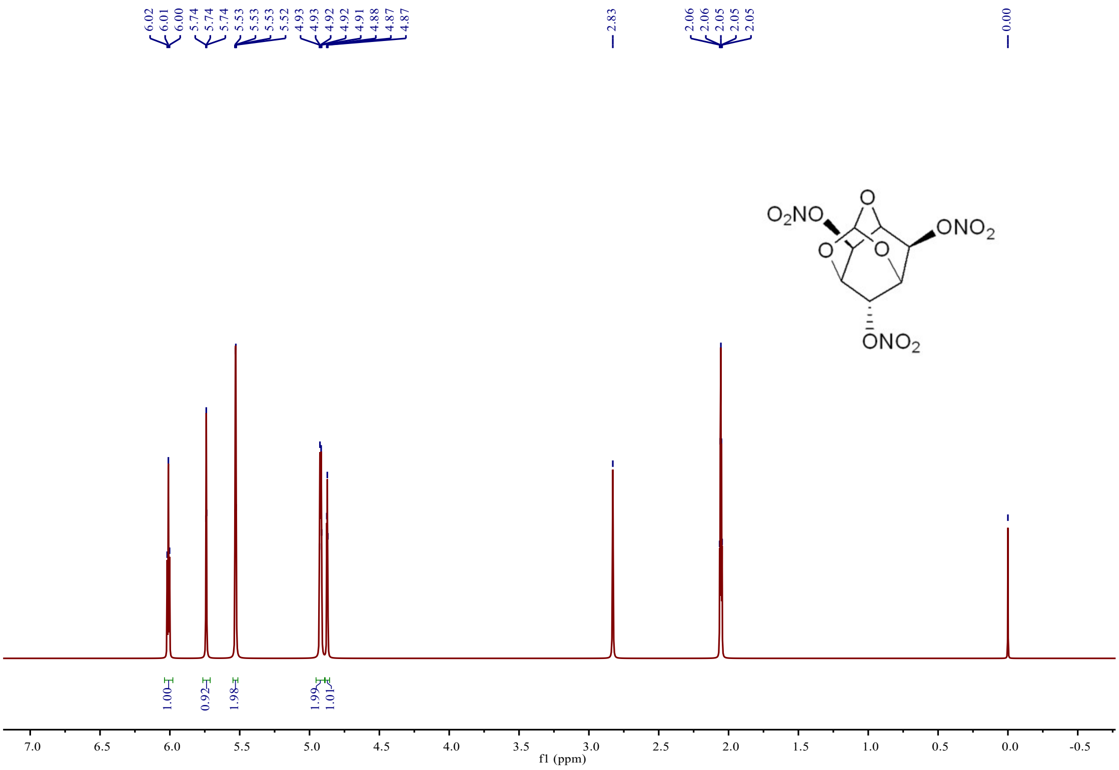

^{1}H NMR spectrum of compound **5** (Acetone-d_6, 500 MHz)

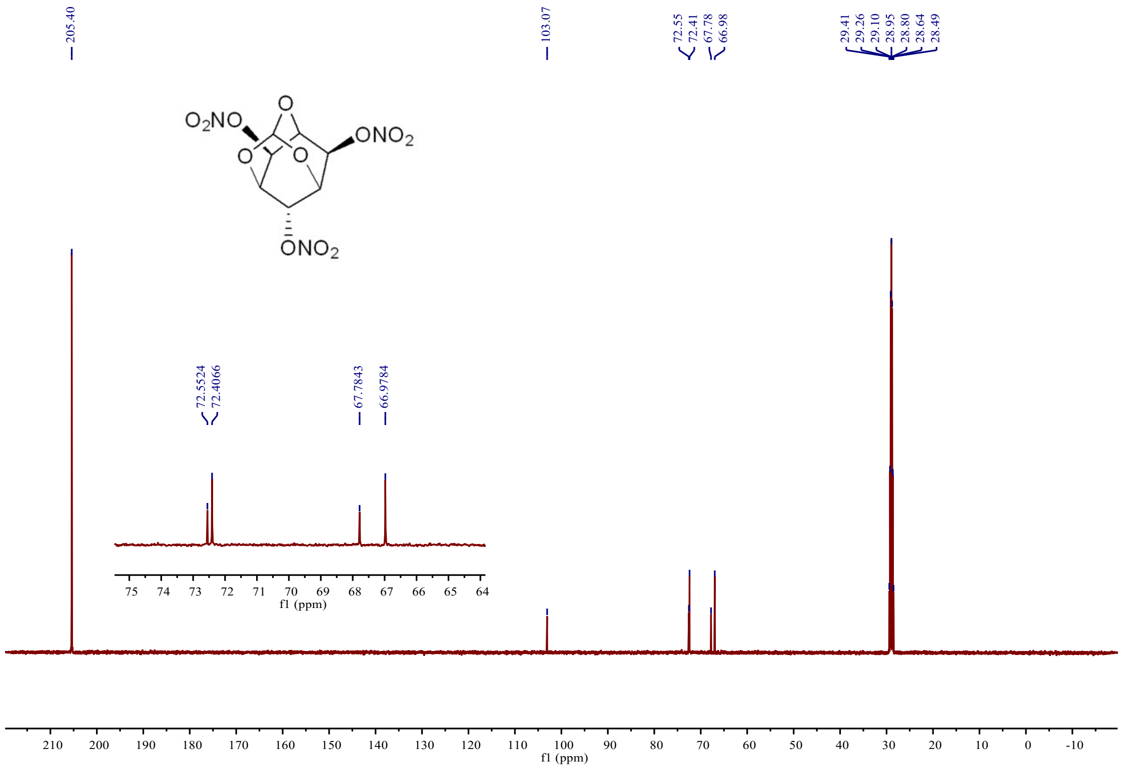

^{13}C NMR spectrum of compound **5** (Acetone-d_6, 126 MHz)

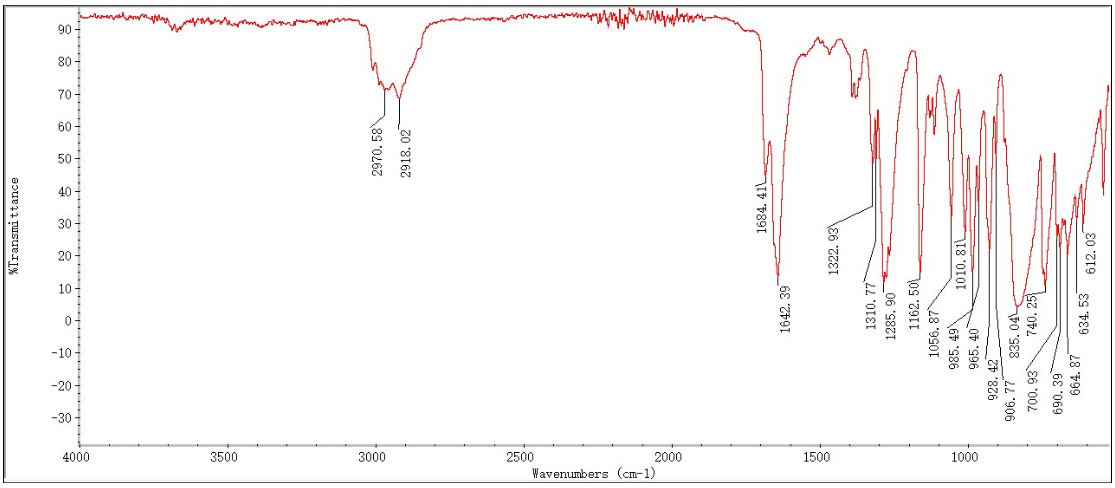

IR spectrum of compound **5**

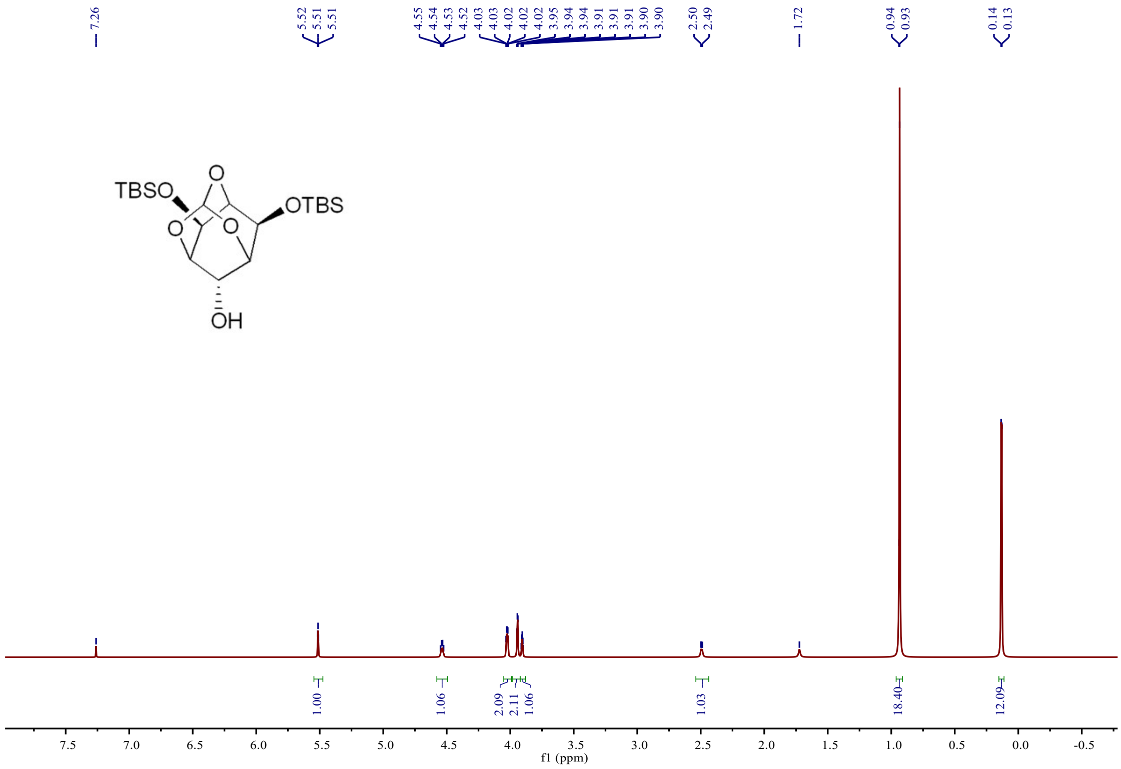

^{1}H NMR spectrum of compound **6** (Chloroform-*d*, 500 MHz)

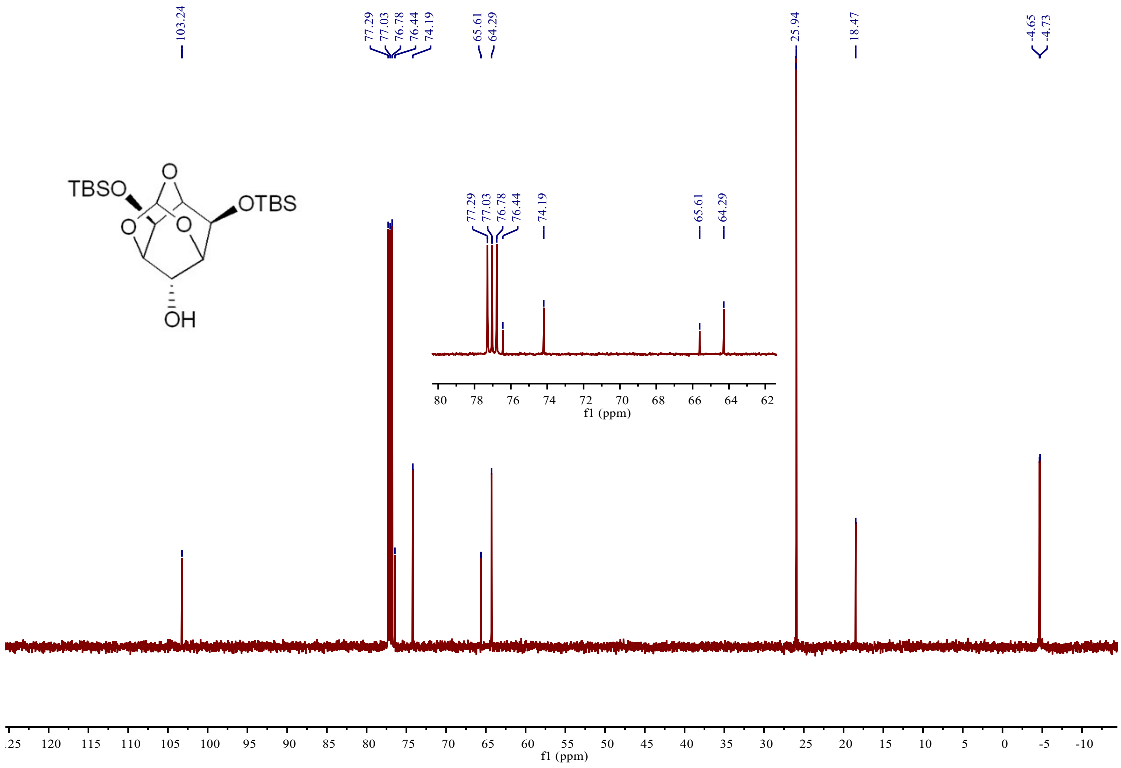

^{13}C NMR spectrum of compound **6** (Chloroform-*d*, 126 MHz)

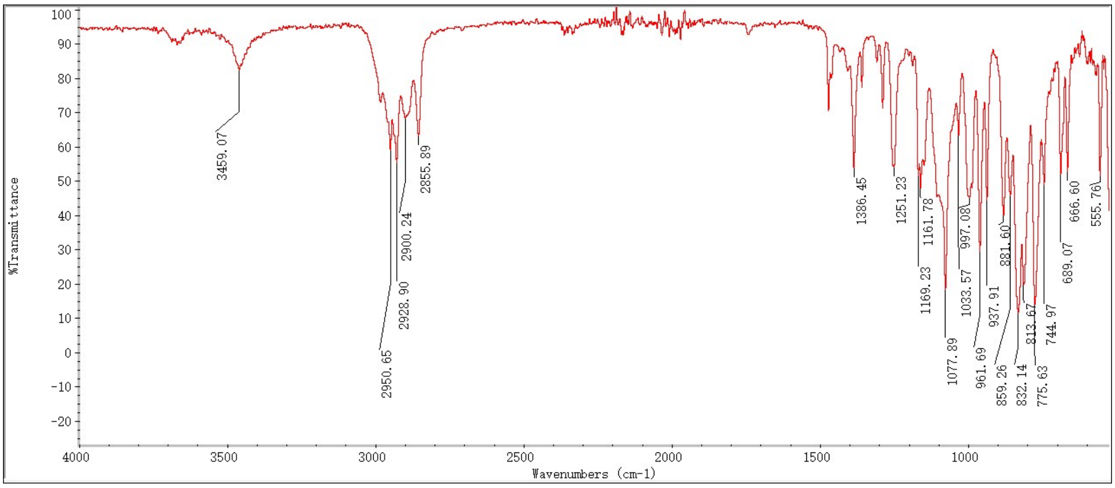

IR spectrum of compound **6**

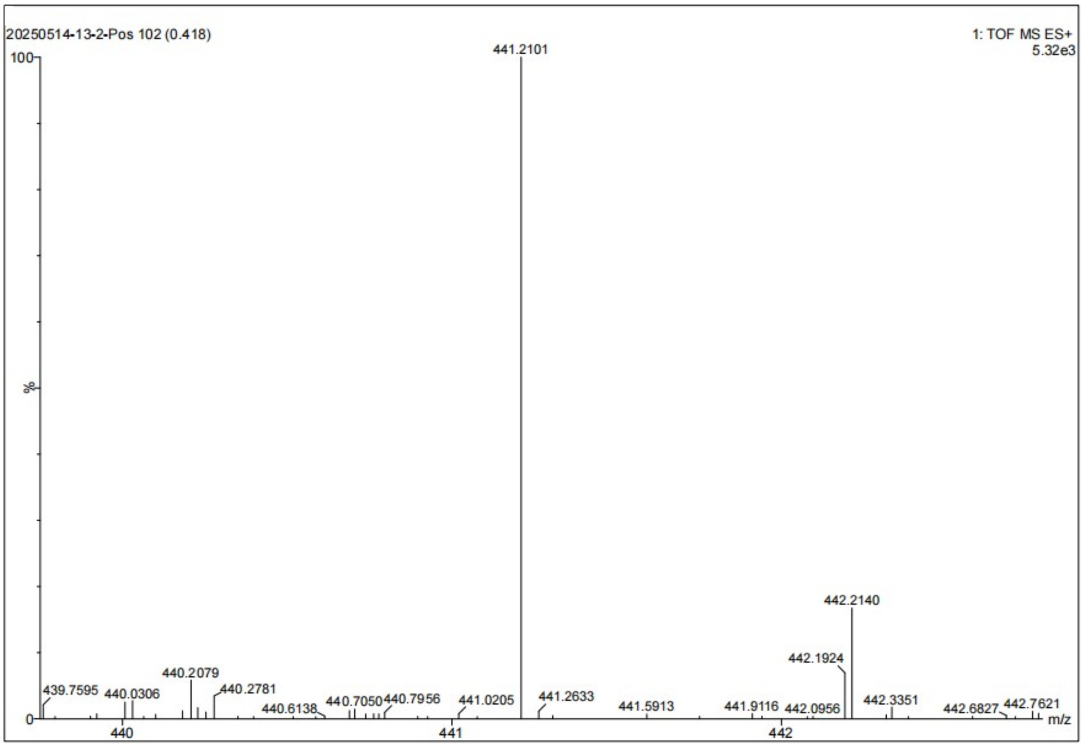

HMRS of compound **6**

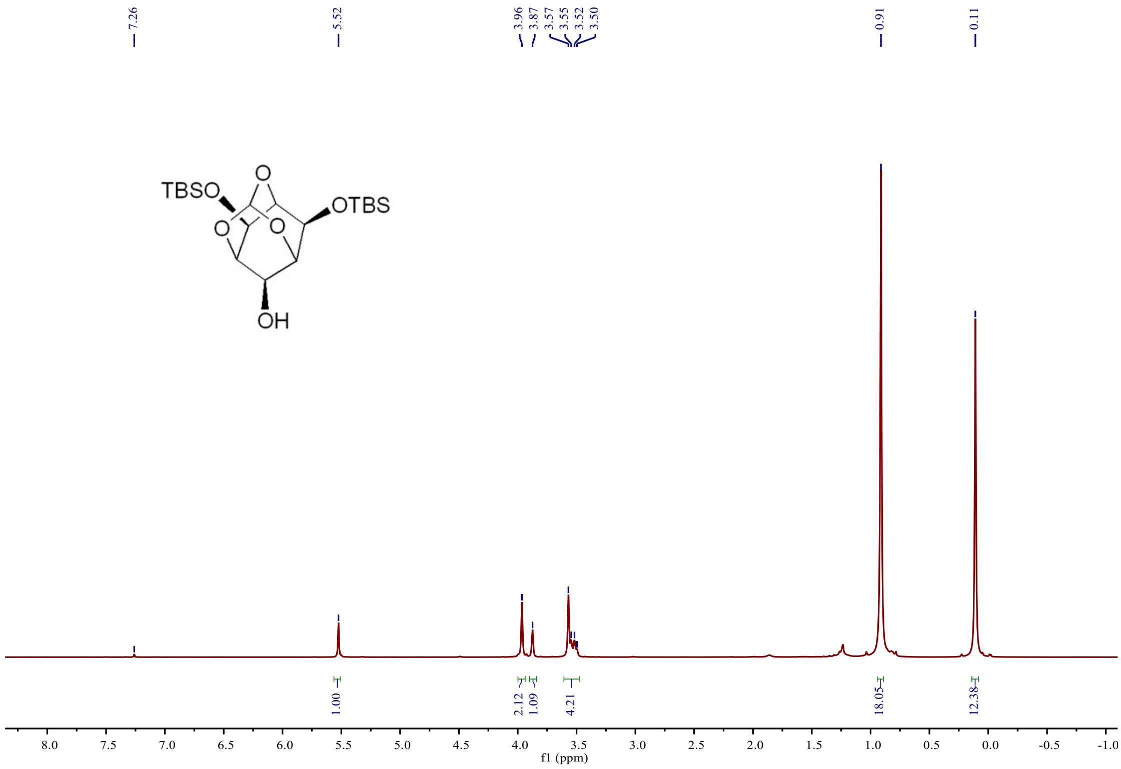

^{1}H NMR spectrum of compound **7** (Chloroform-*d*, 500 MHz)

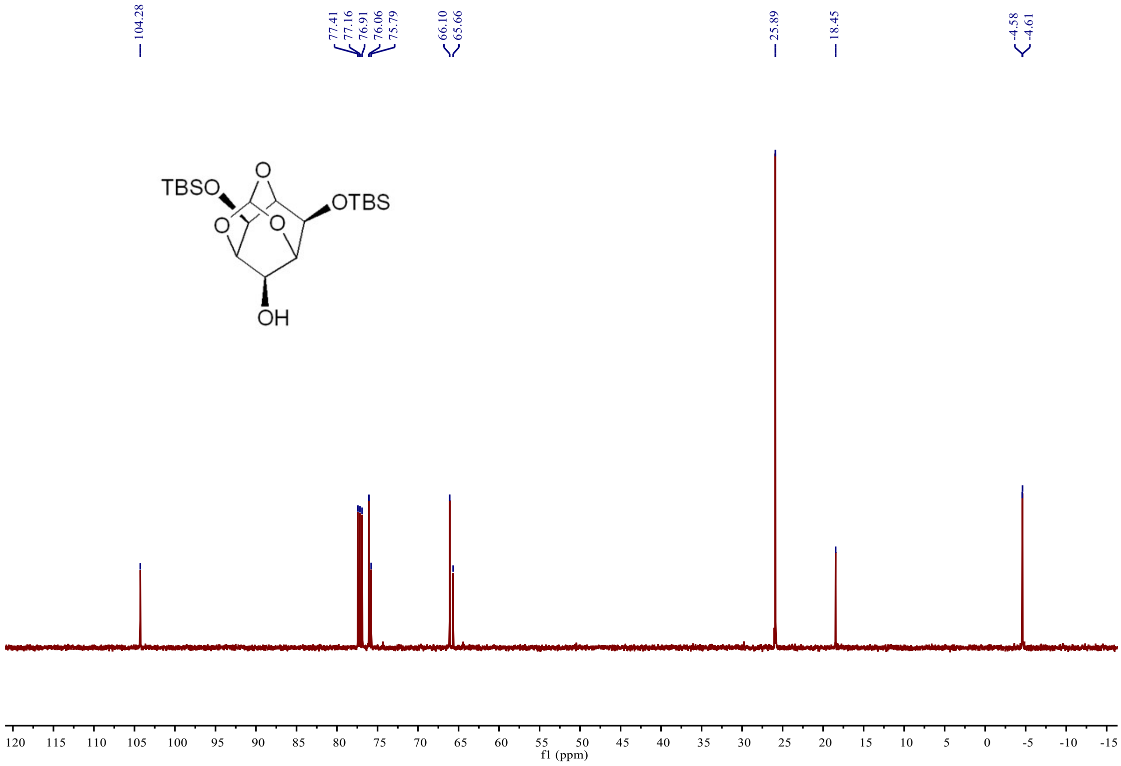

^{13}C NMR spectrum of compound **7** (Chloroform-*d*, 126 MHz)

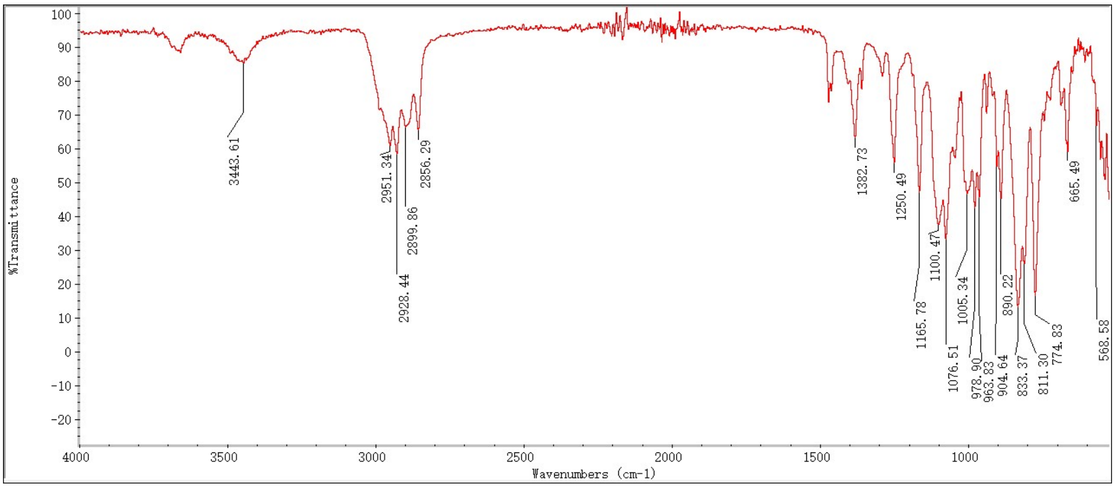

IR spectrum of compound **7**

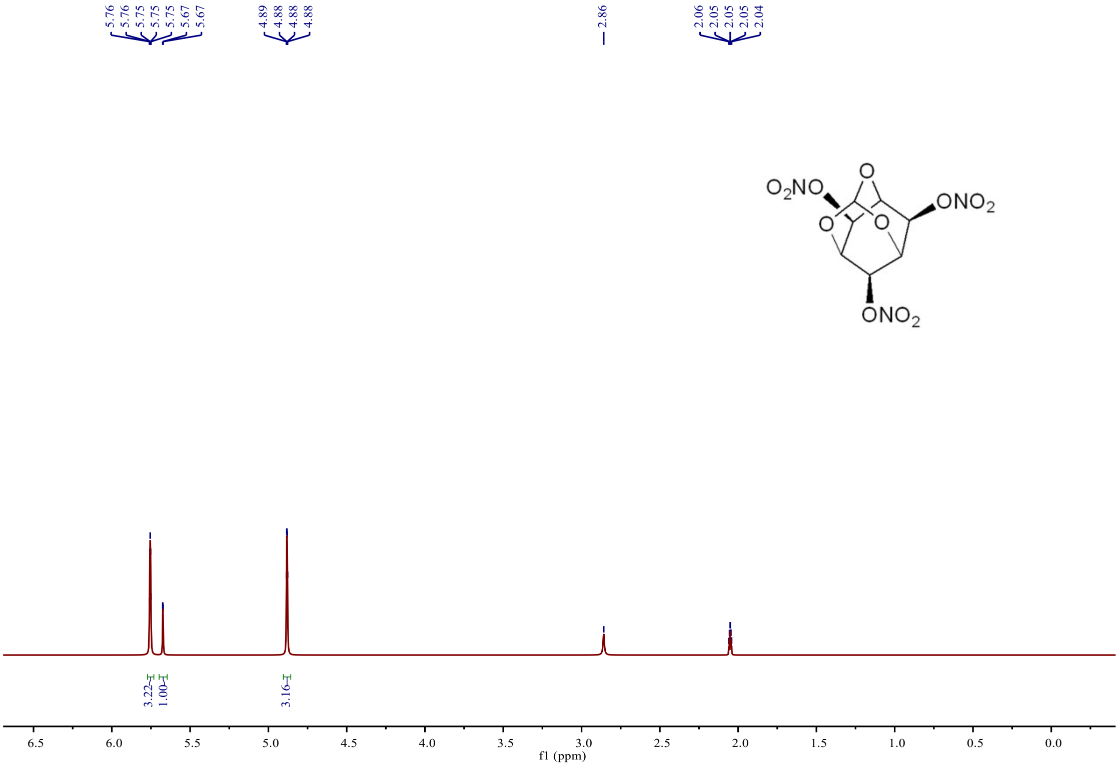

^{1}H NMR spectrum of compound **8** (Acetone-d_6, 500 MHz)

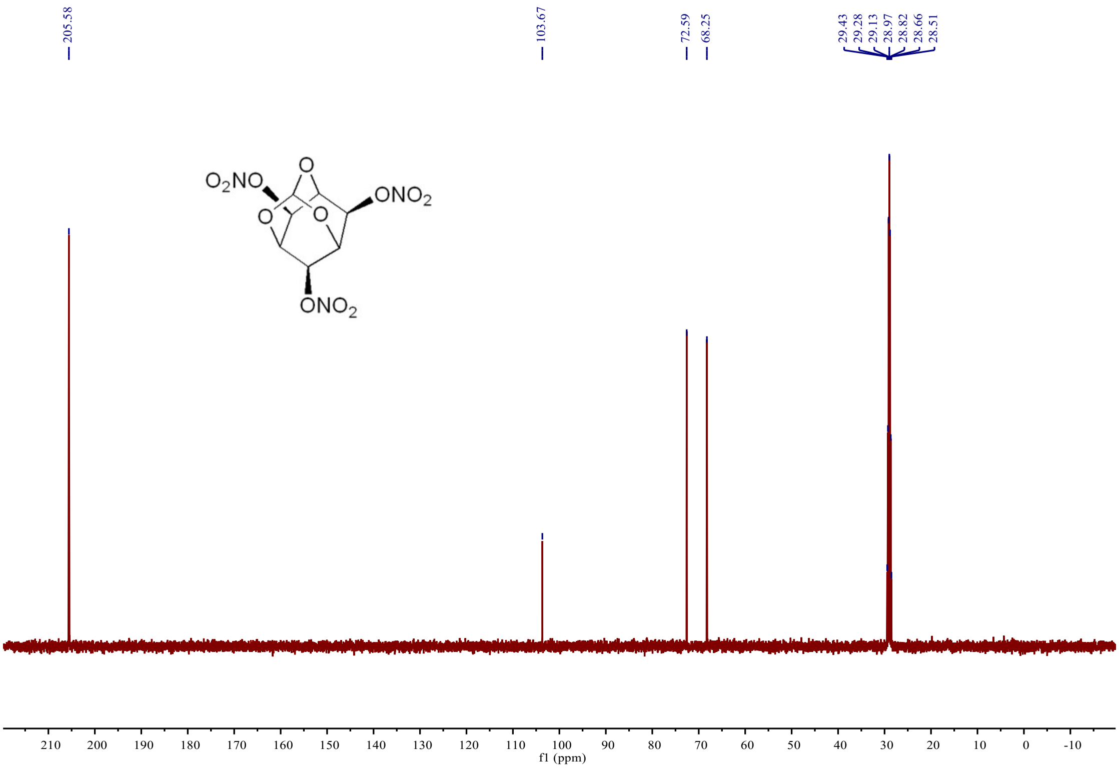

^{13}C NMR spectrum of compound **8** (Acetone-d_6, 126 MHz)

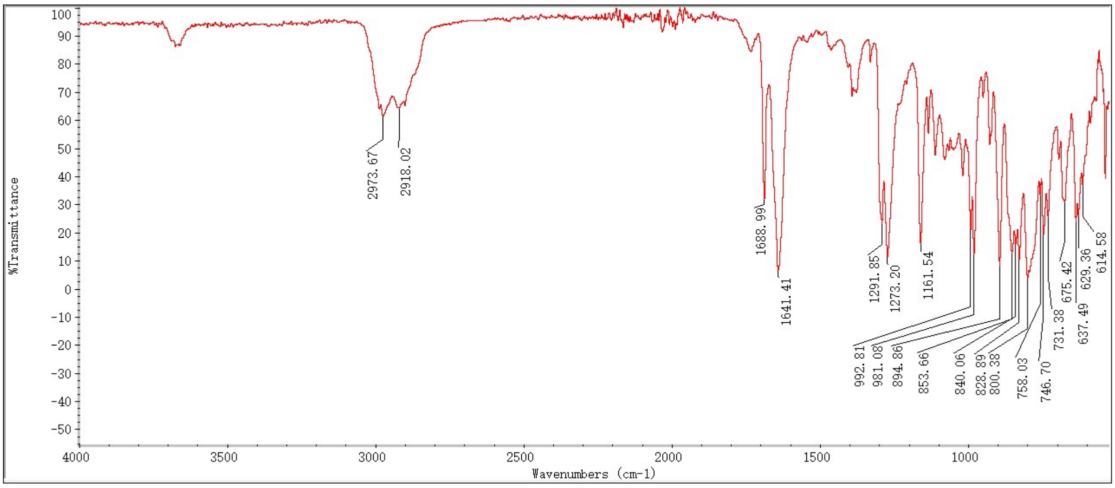

IR spectrum of compound **8**

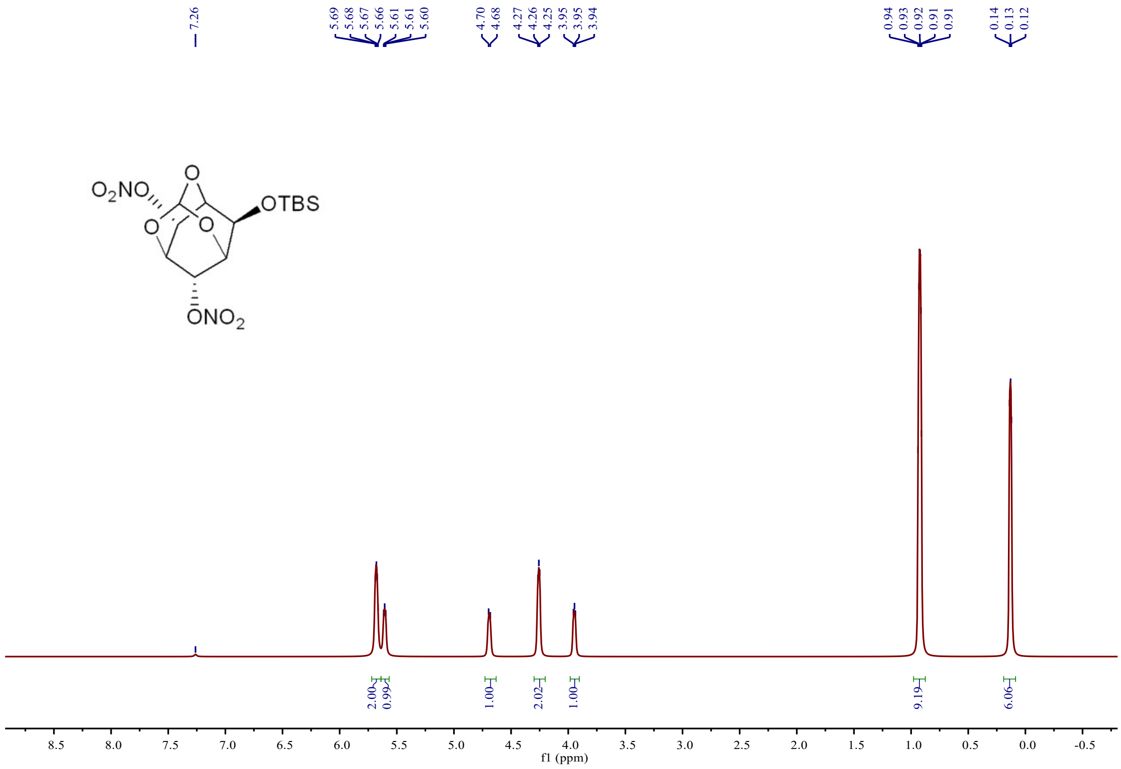

^{1}H NMR spectrum of compound **9** (Chloroform-*d*, 500 MHz)

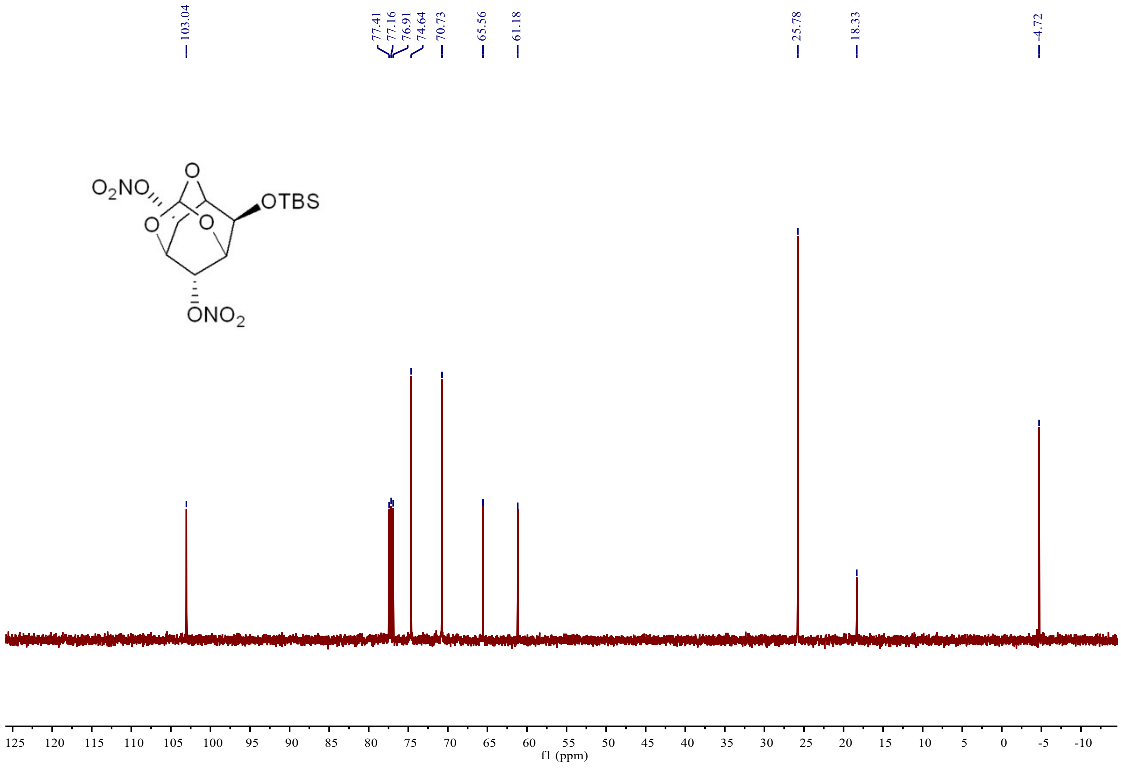

^{13}C NMR spectrum of compound **9** (Chloroform-*d*, 126 MHz)

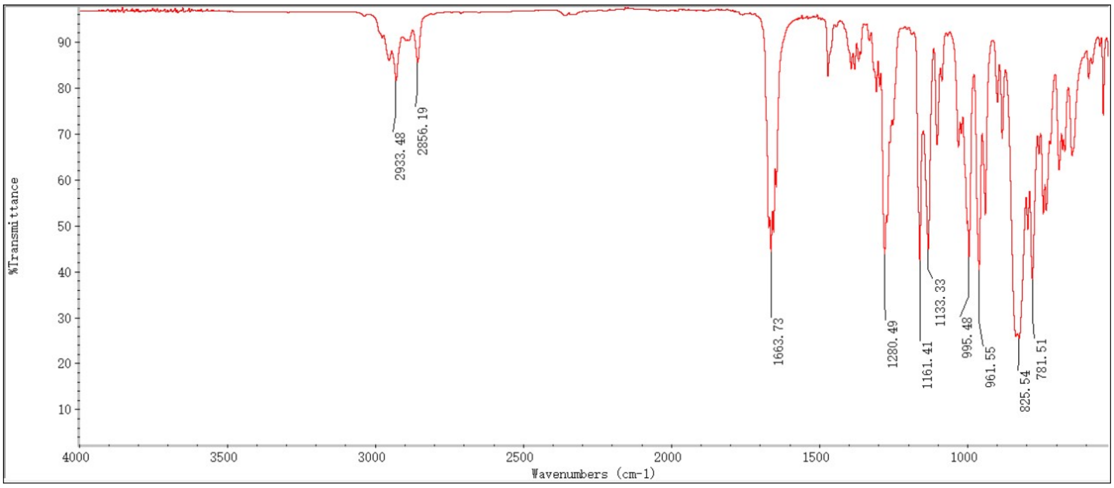

IR spectrum of compound **9**

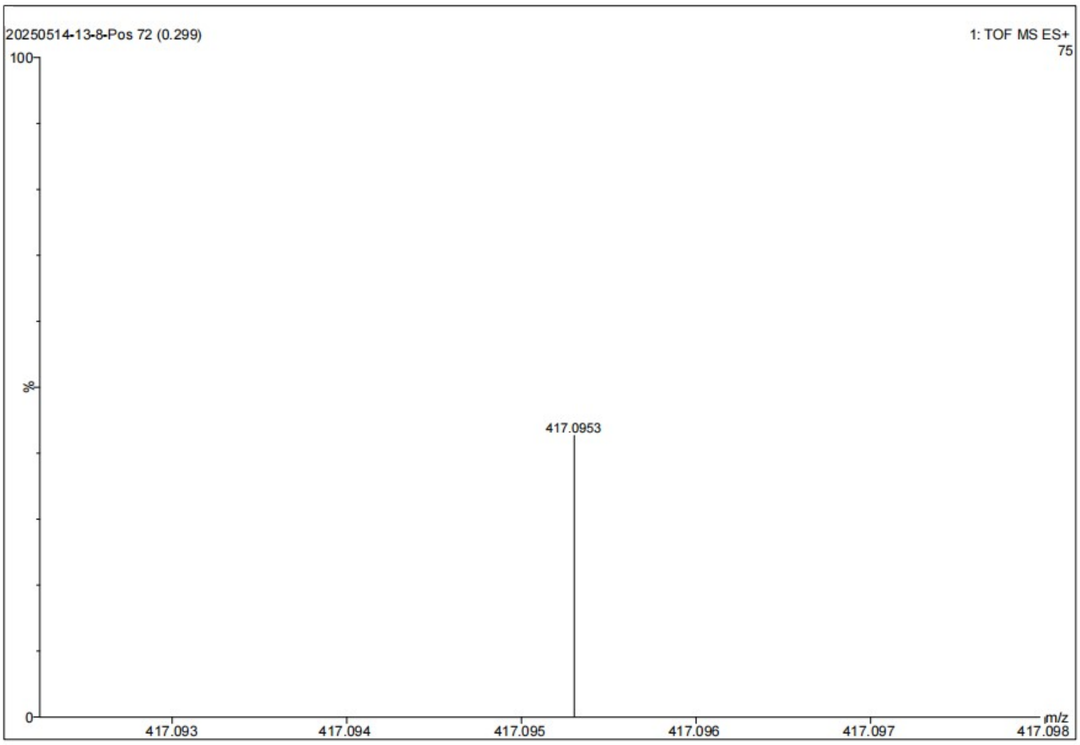

HMRS of compound **9**

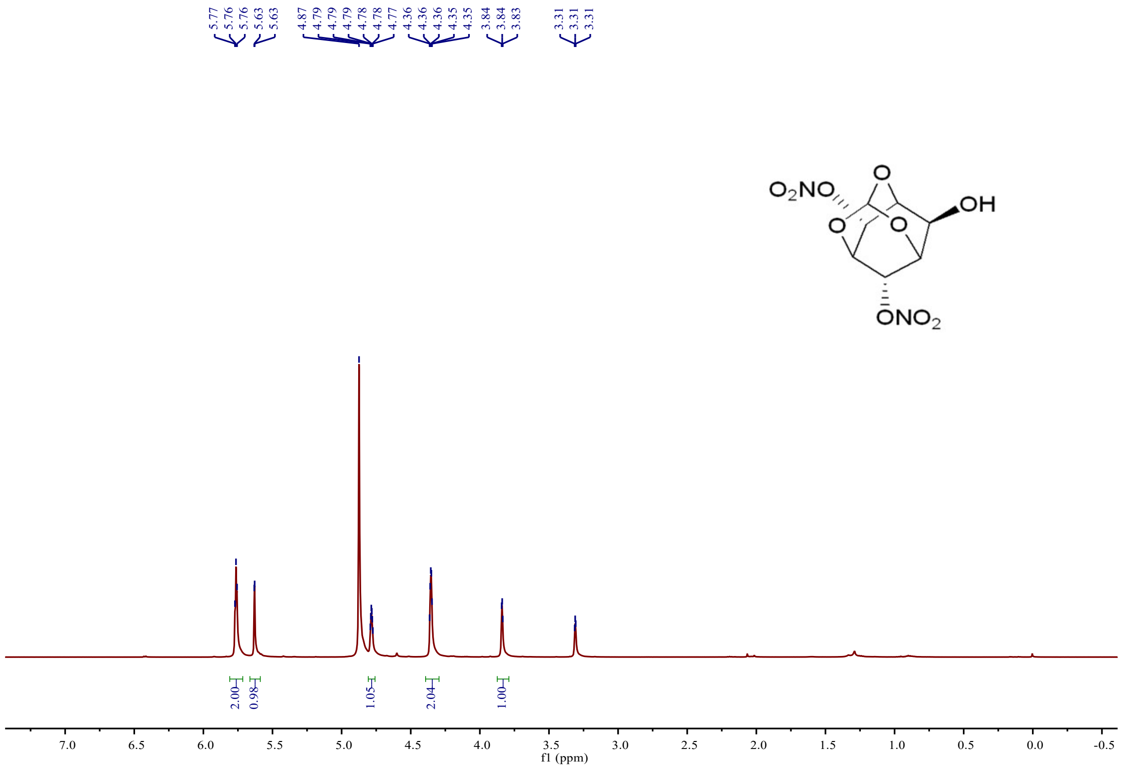

^{1}H NMR spectrum of compound **10** (Methanol-d_4, 500 MHz)

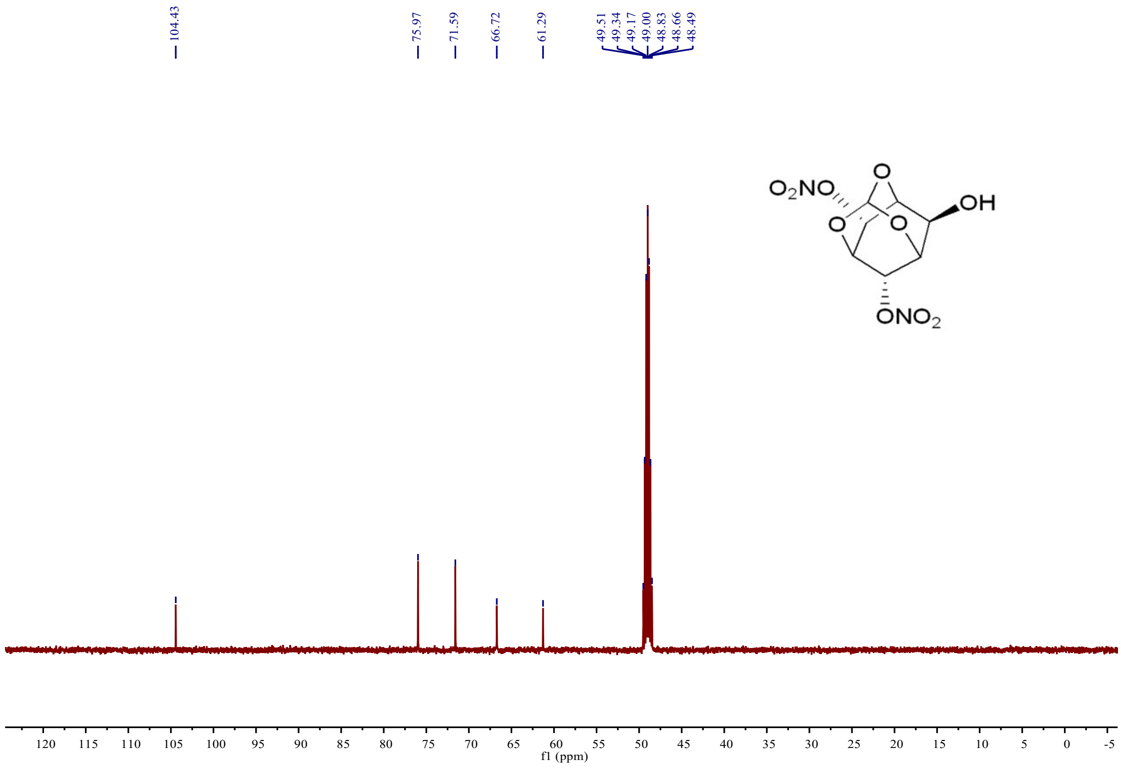

^{13}C NMR spectrum of compound **10** (Methanol-d_4, 126 MHz)

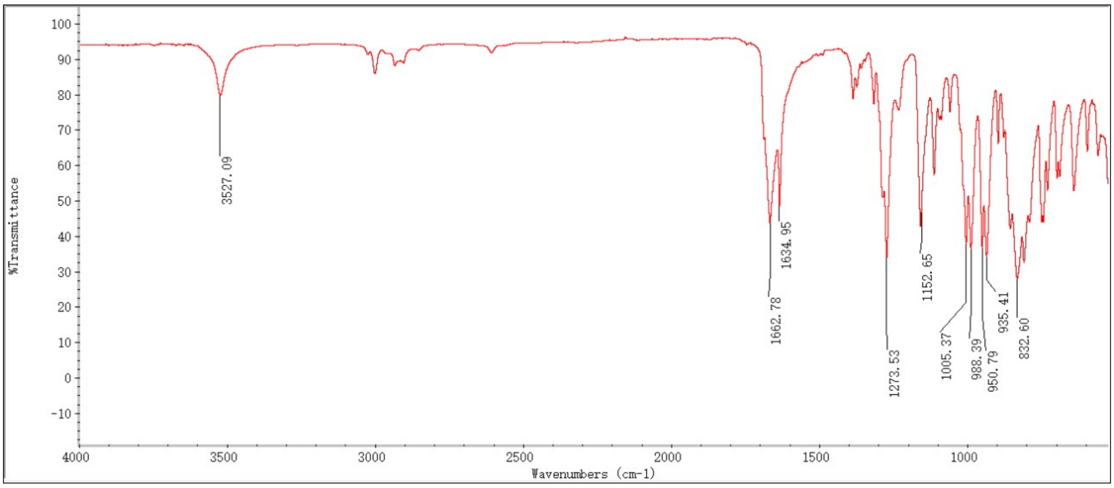

IR spectrum of compound **10**

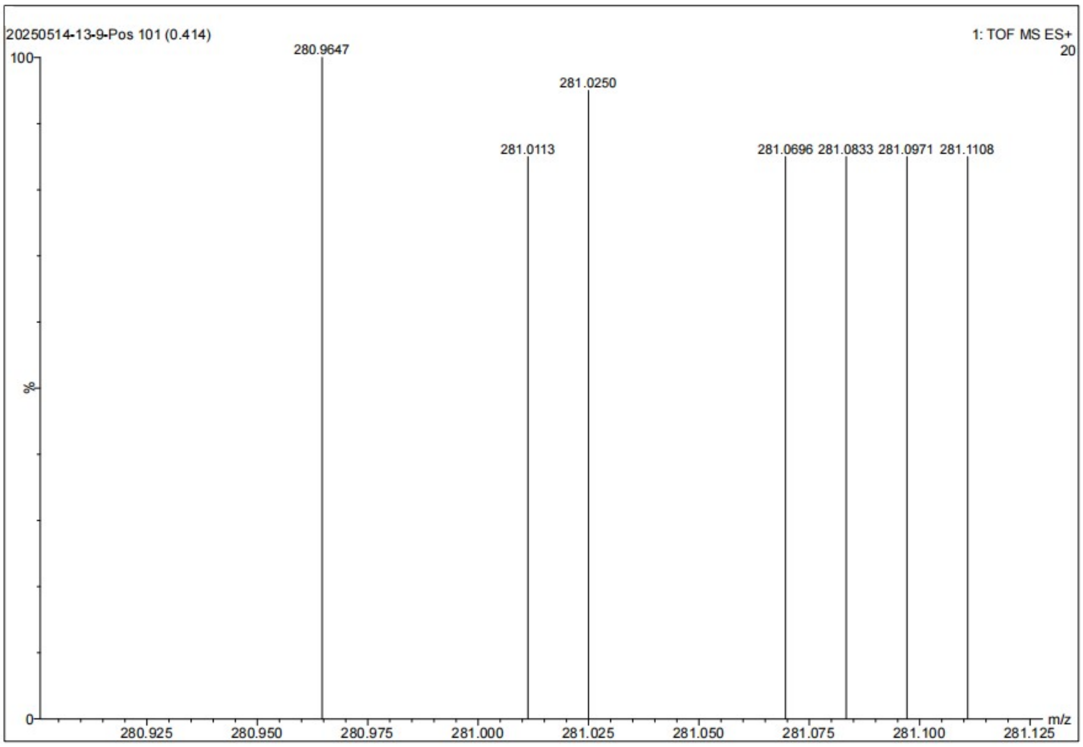

HMRS of compound **10**

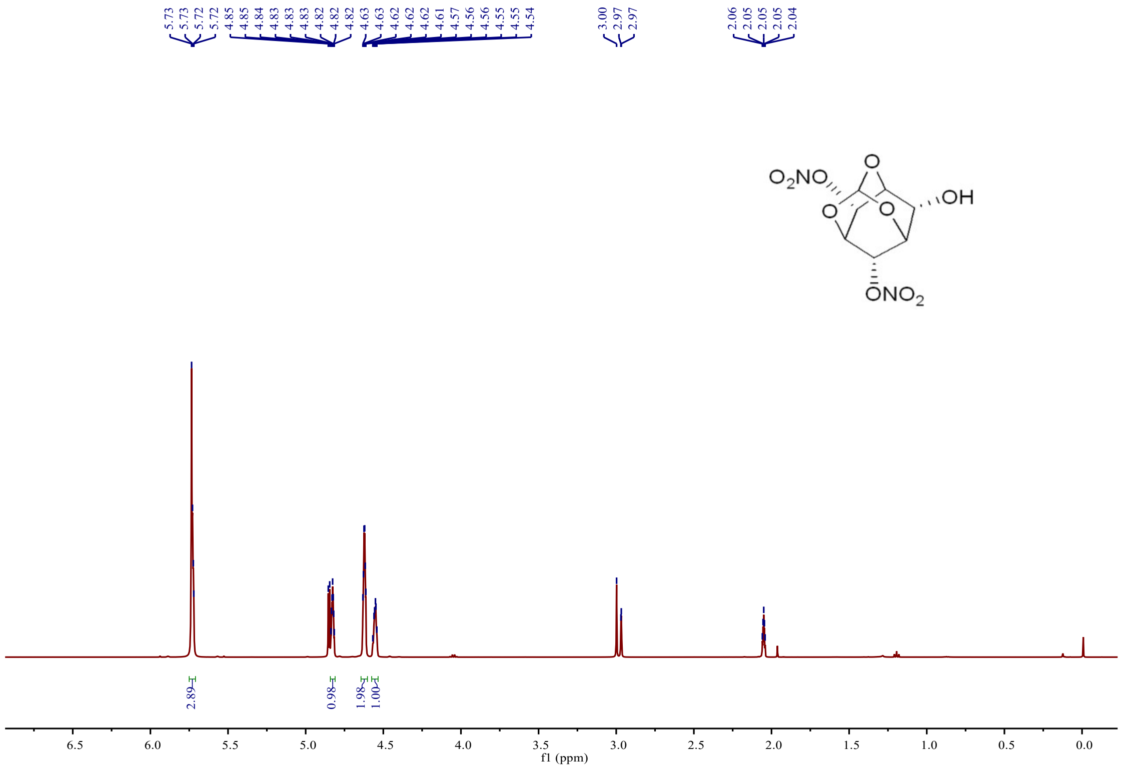

^{1}H NMR spectrum of compound **11** (Acetone-d_6, 500 MHz)

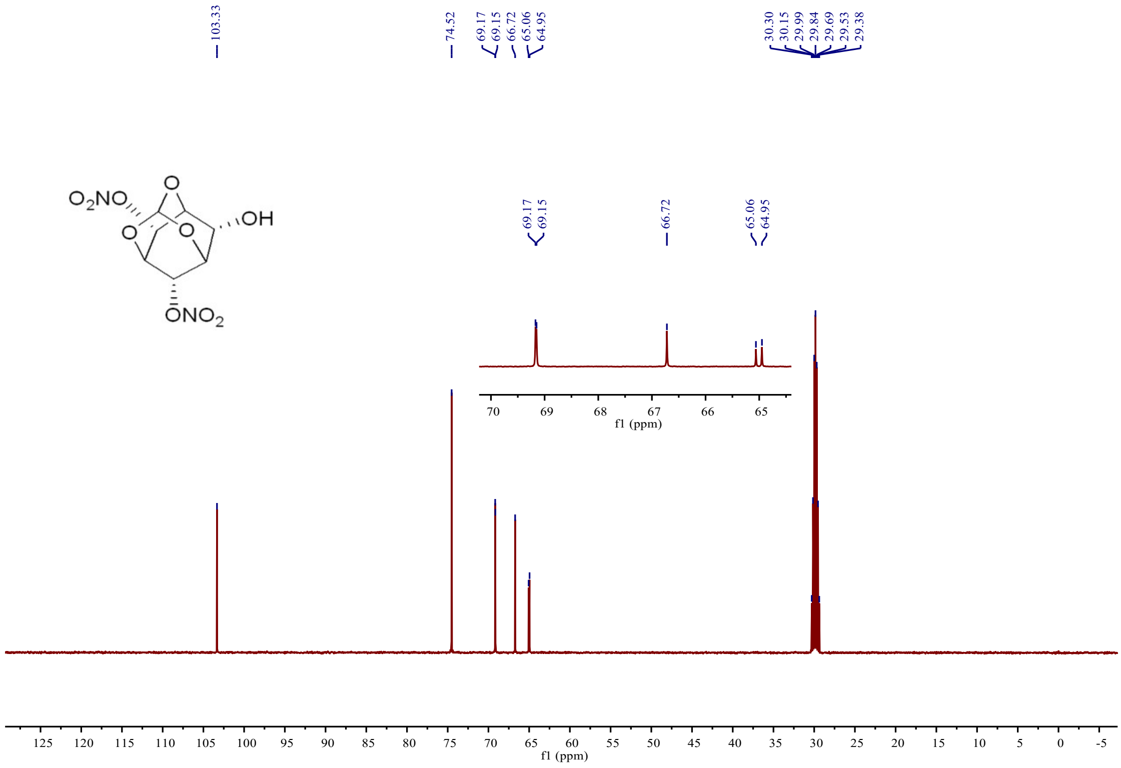

^{13}C NMR spectrum of compound **11** (Acetone-d_6, 126 MHz)

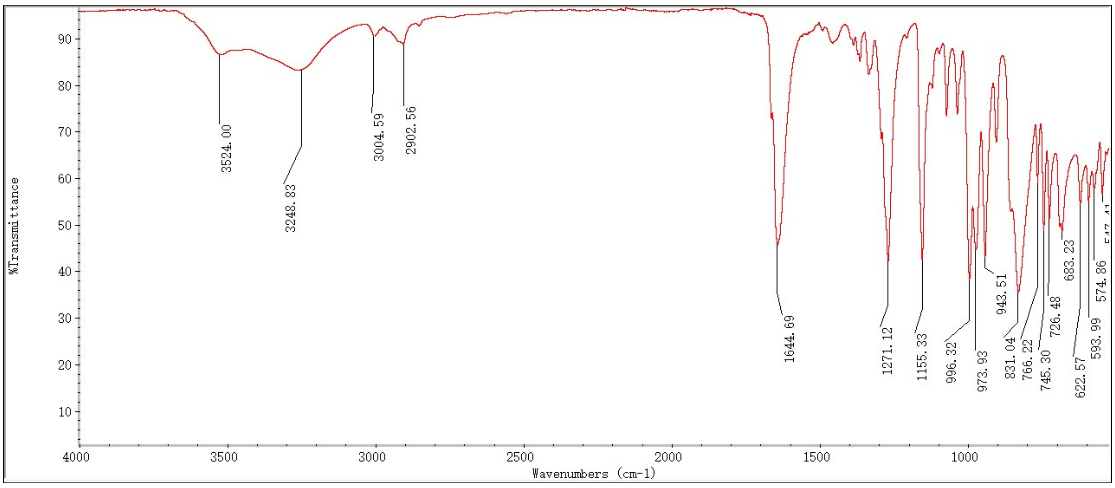

IR spectrum of compound **11**

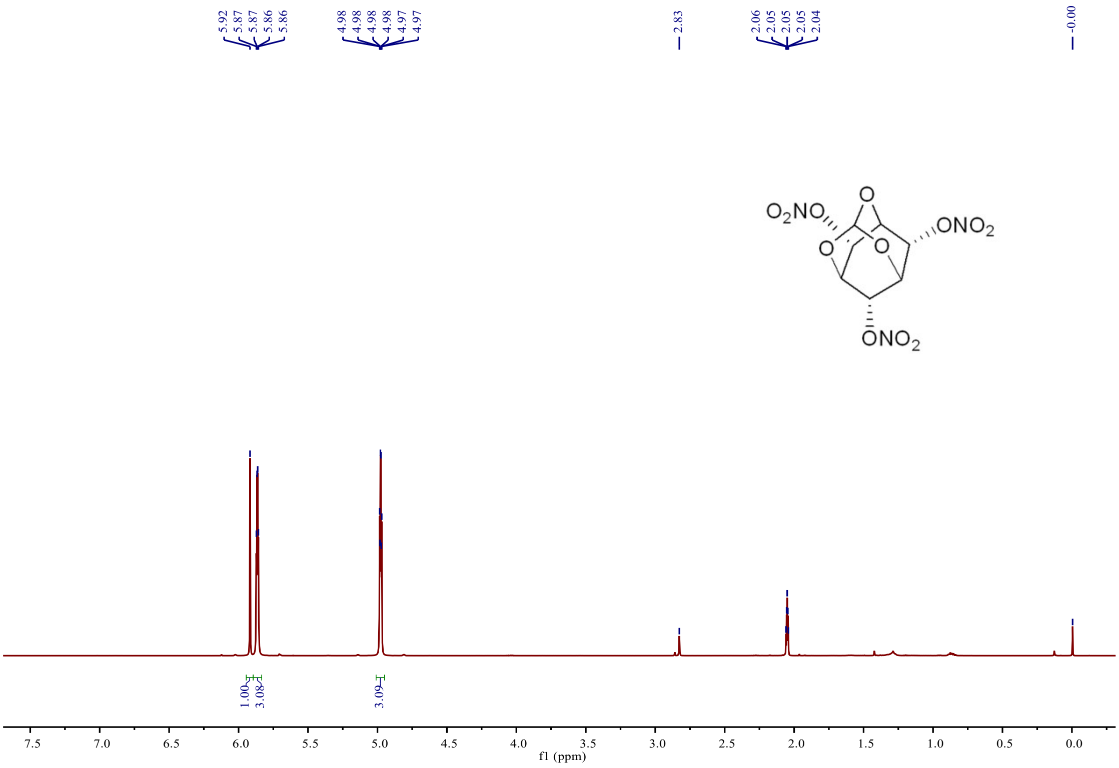

^{1}H NMR spectrum of compound **12** (Acetone-d_6, 500 MHz)

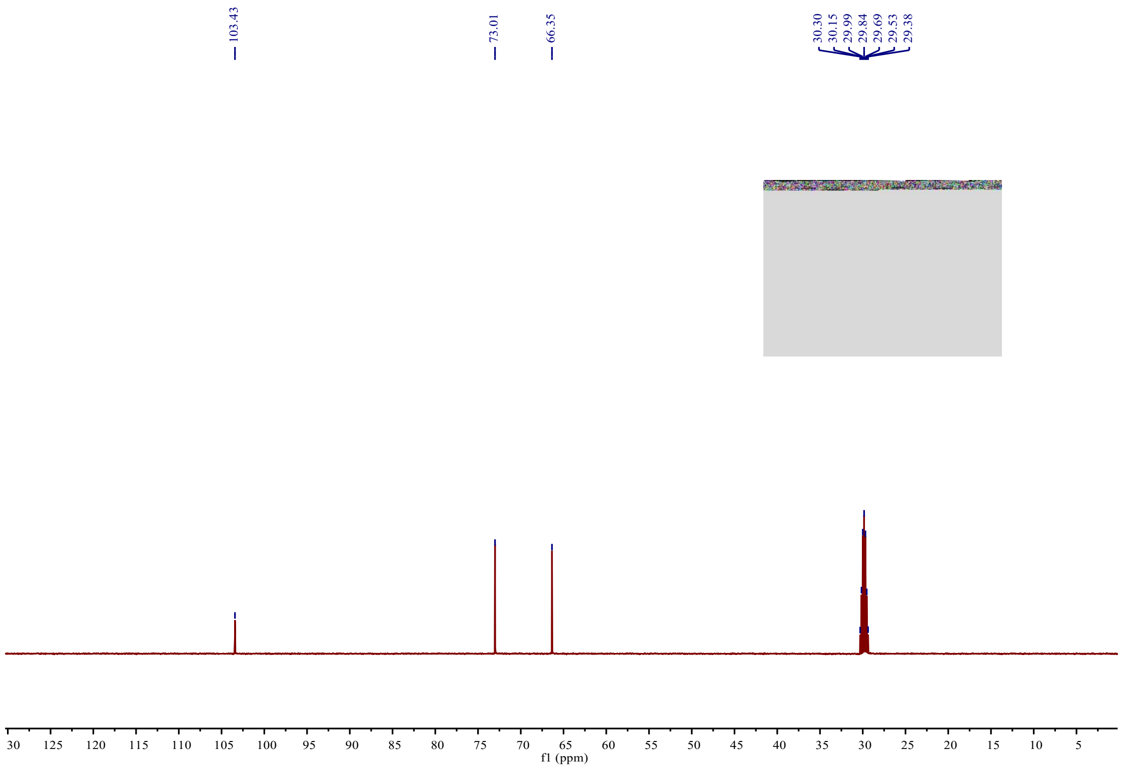

^{13}C NMR spectrum of compound **12** (Acetone-d_6, 126 MHz)

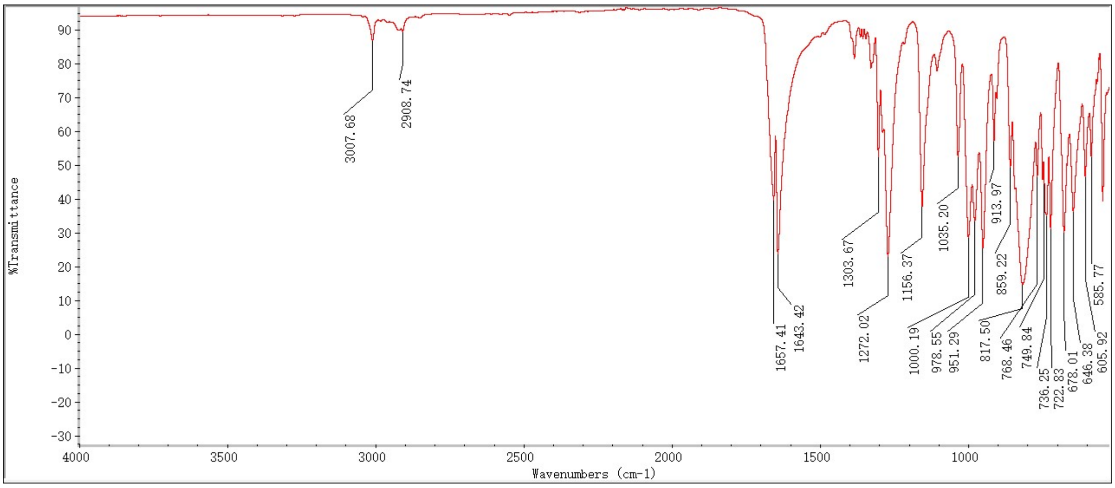

IR spectrum of compound **12**

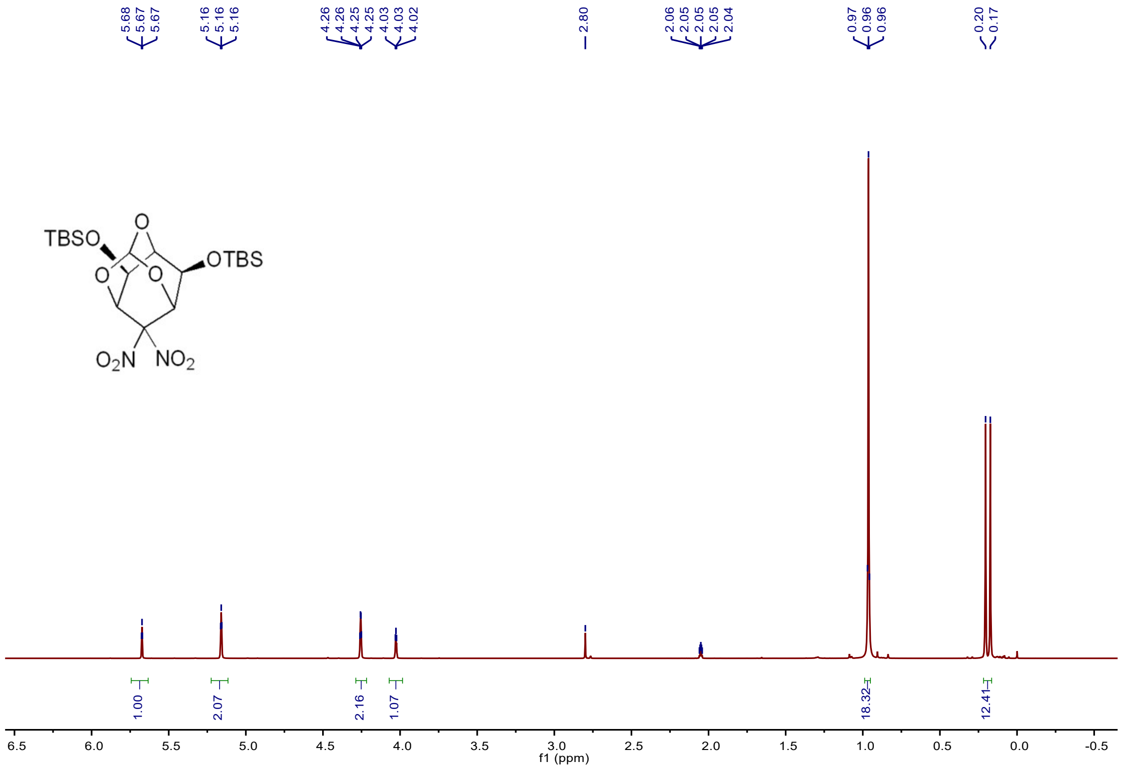

^1H NMR spectrum of compound **13** (Acetone-d_6,500 MHz)

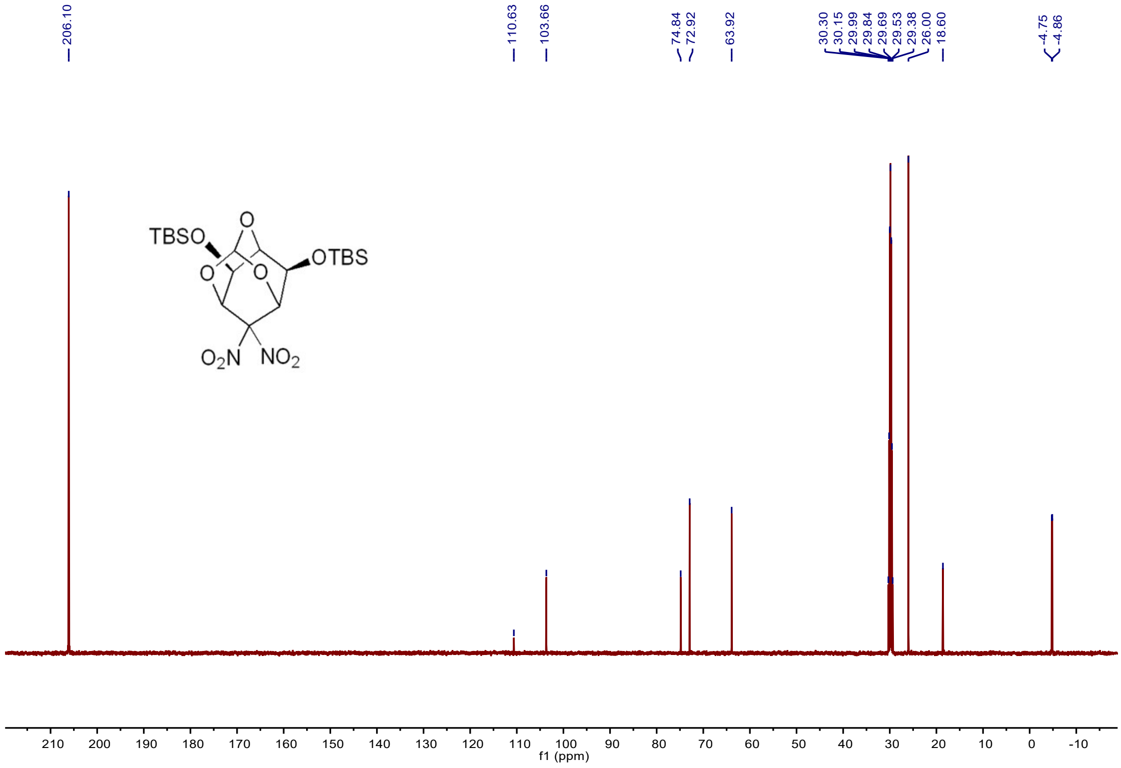

^{13}C NMR spectrum of compound **13** (Acetone-d_6, 126 MHz)

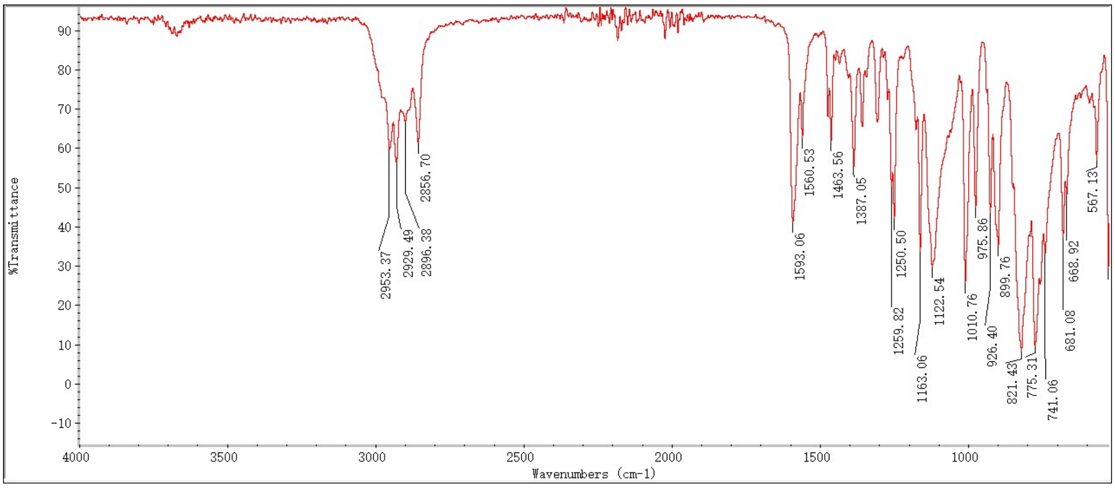

IR spectrum of compound **13**

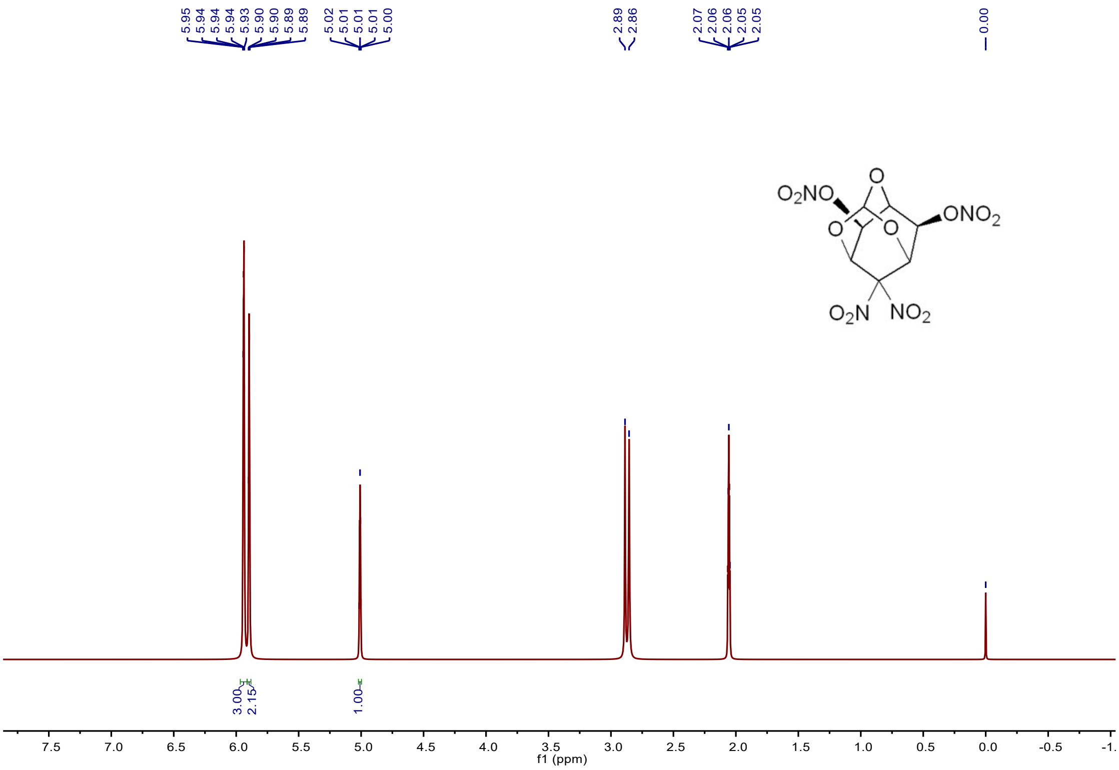

^{1}H NMR spectrum of compound **14** (Acetone-d_6, 500 MHz)

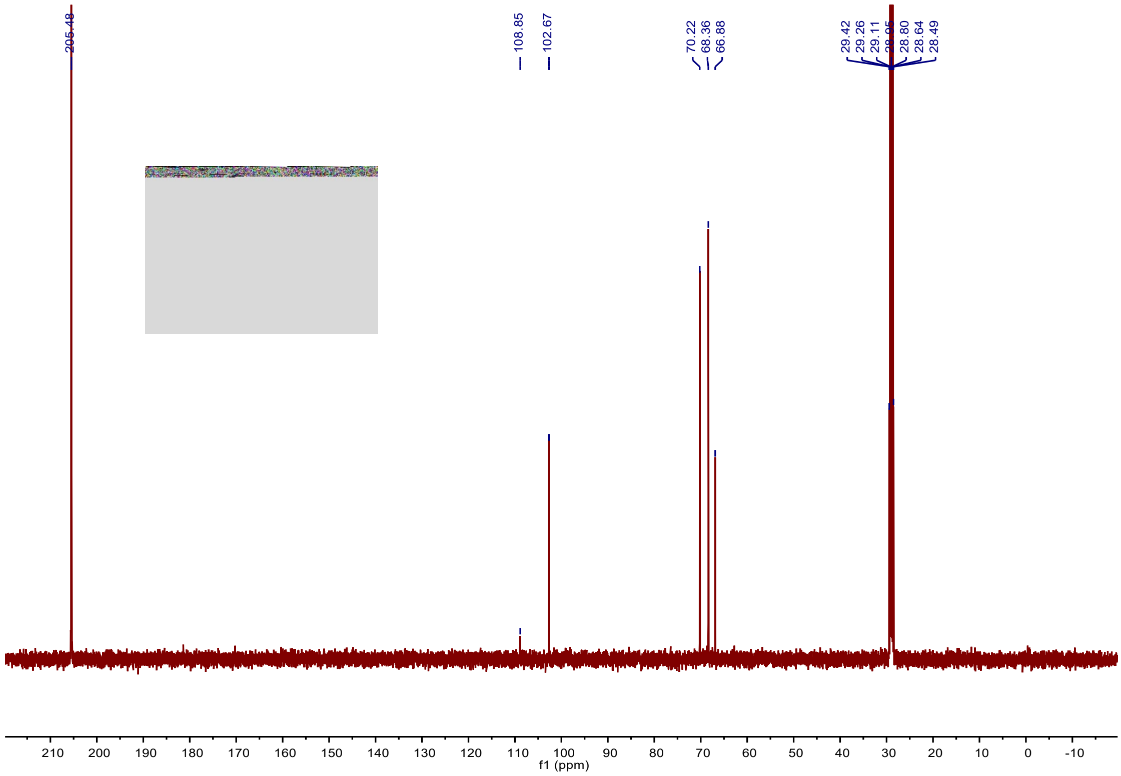

^{13}C NMR spectrum of compound **14** (Acetone-d_6, 126 MHz)

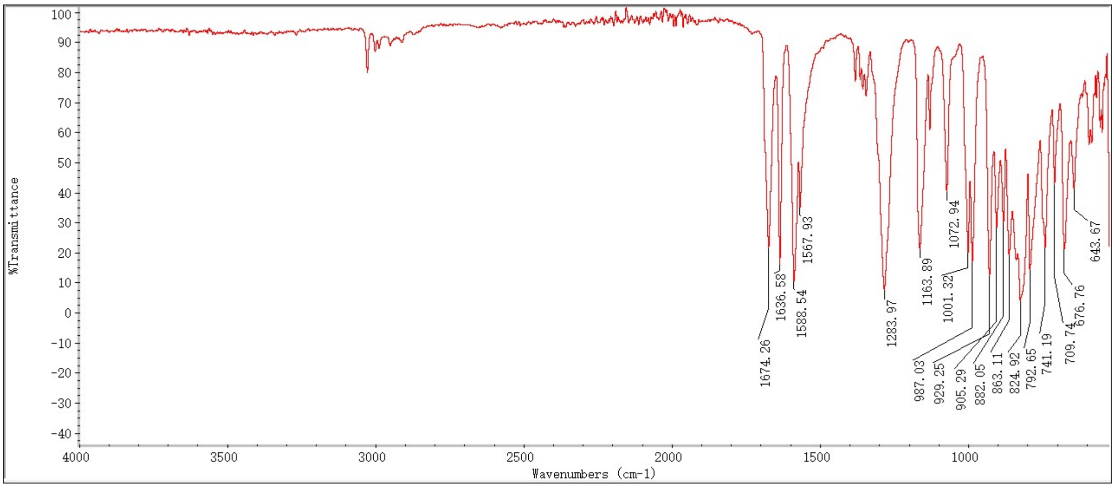

IR spectrum of compound **14**

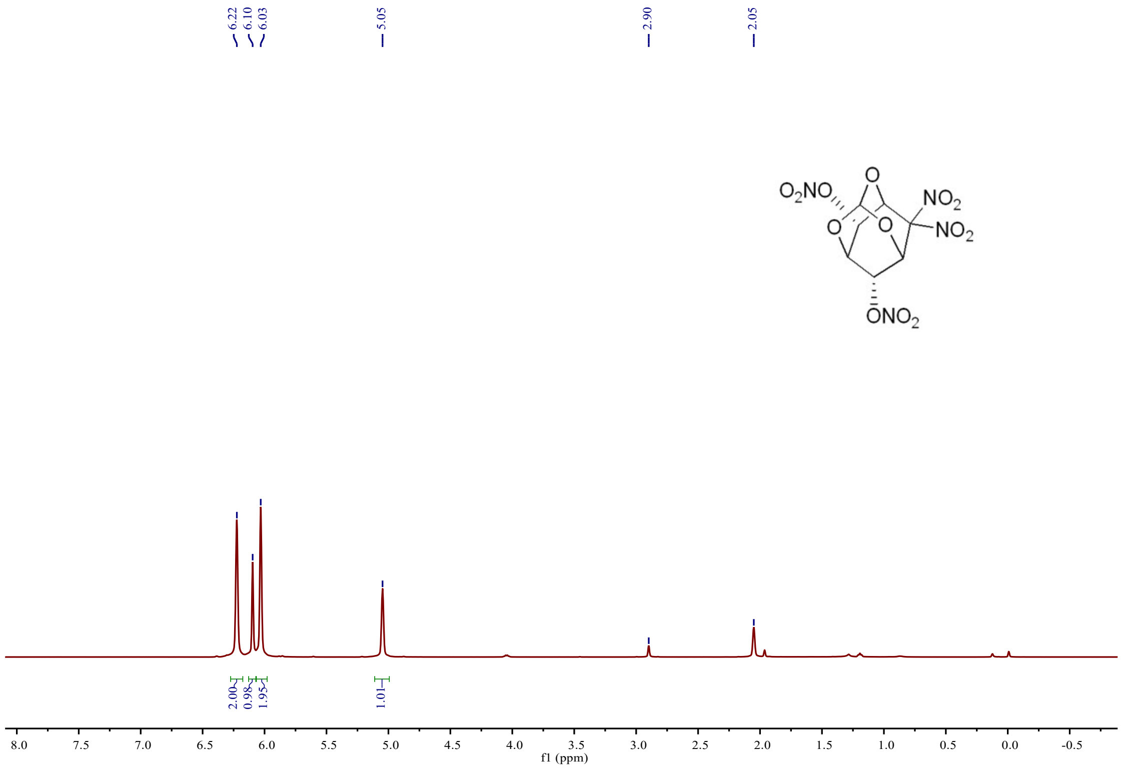

^{1}H NMR spectrum of compound **15** (Acetone-d_6, 500 MHz)

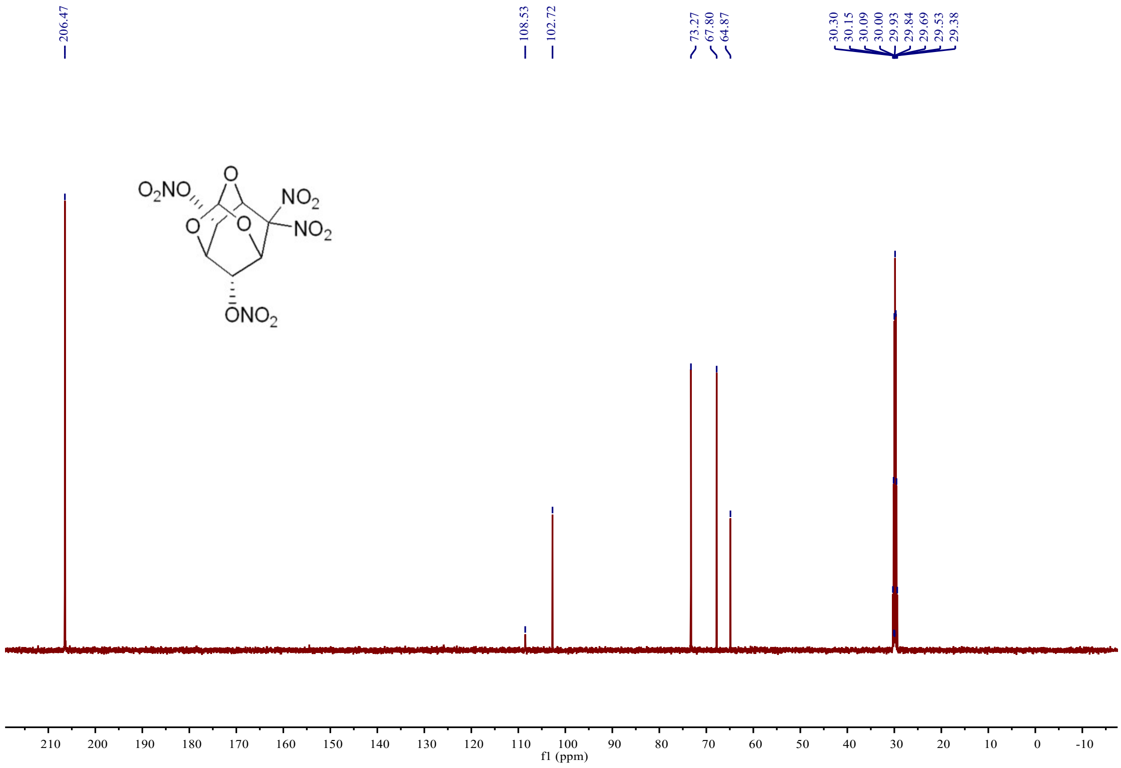

^{13}C NMR spectrum of compound **15** (Acetone-d_6, 126 MHz)

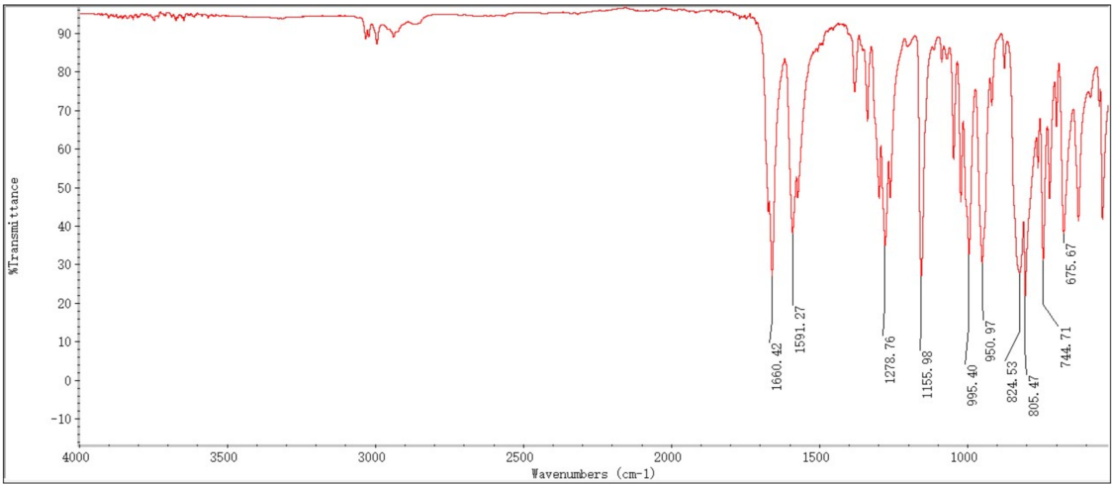

IR spectrum of compound **15**

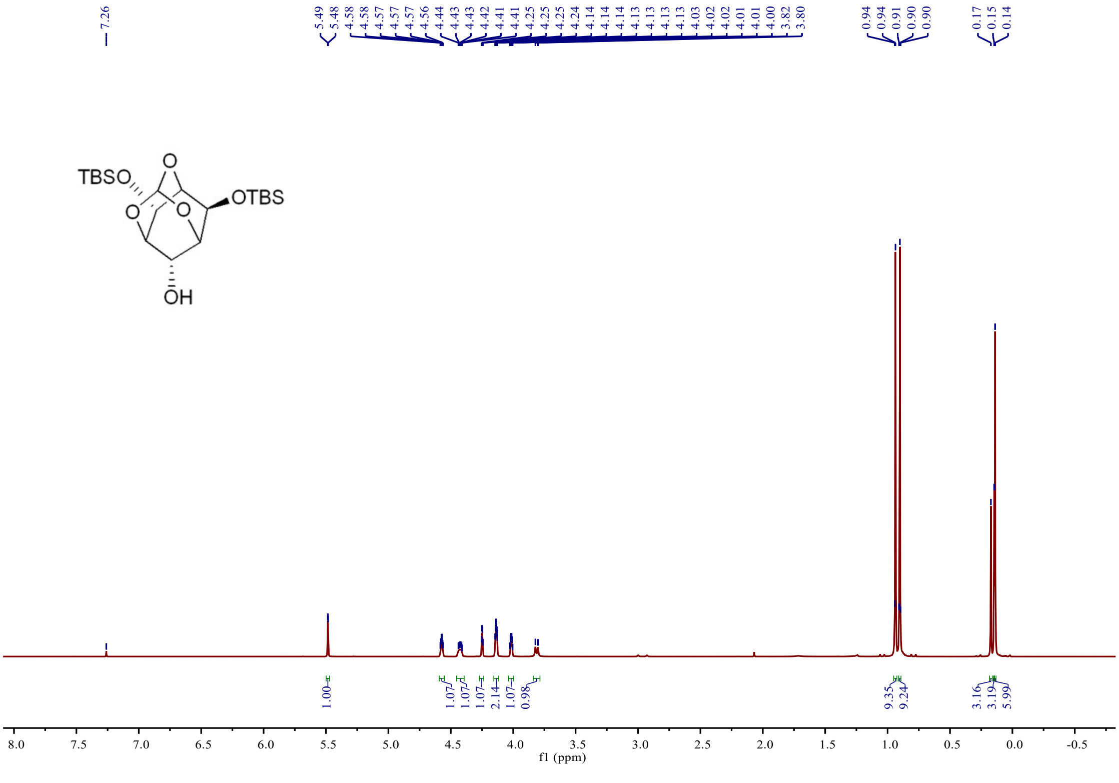

^{1}H NMR spectrum of compound **S1** (Chloroform-d, 500 MHz)

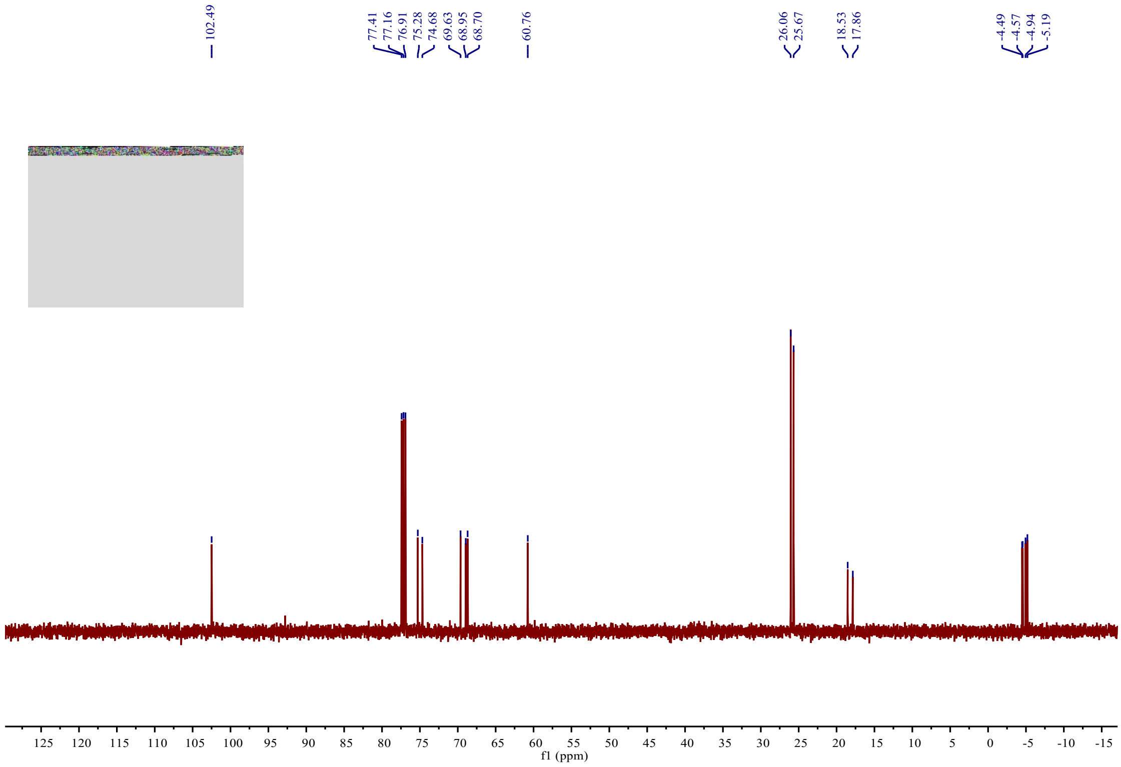

^{13}C NMR spectrum of compound **S1** (Chloroform-*d*, 126 MHz)

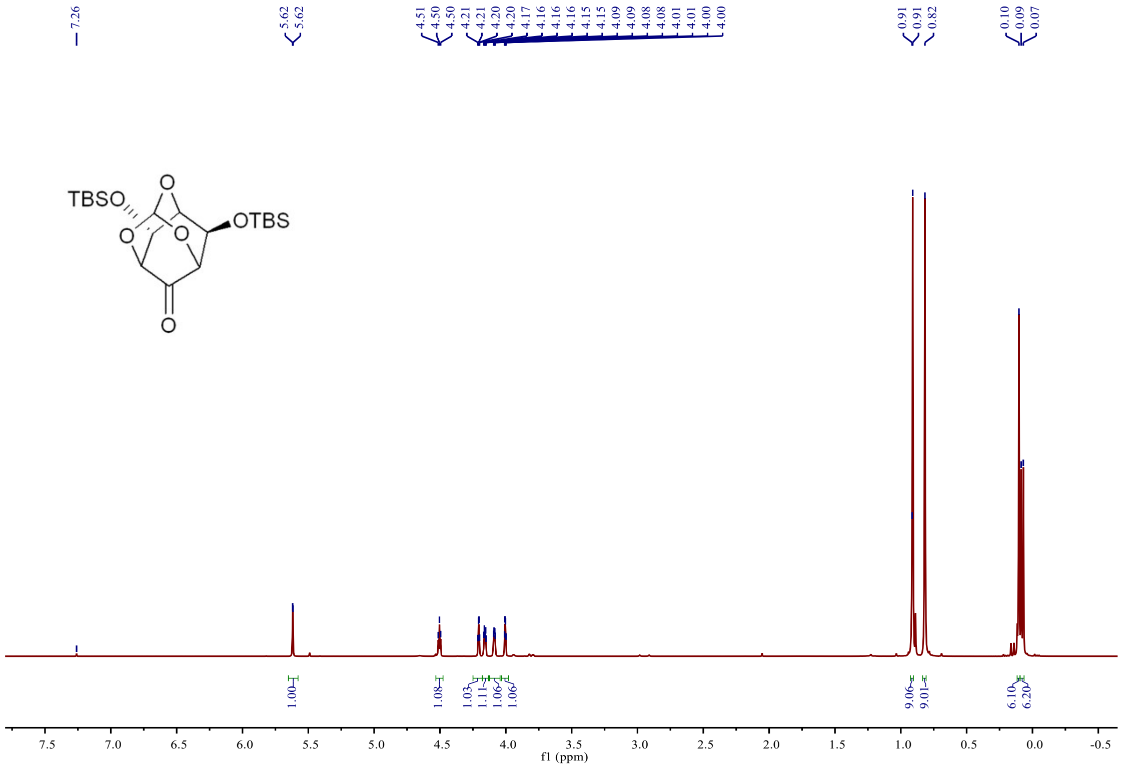

^{1}H NMR spectrum of compound **16** (Chloroform-*d*, 500 MHz)

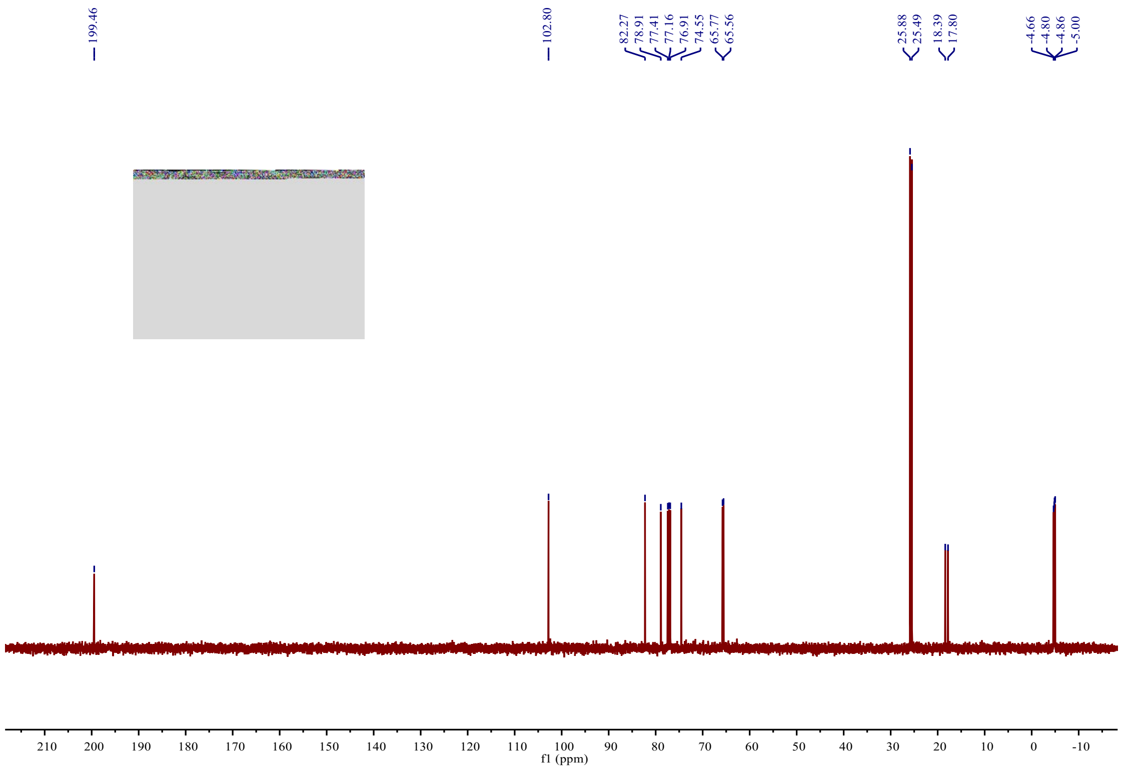

^{13}C NMR spectrum of compound **16** (Chloroform-*d*, 126 MHz)

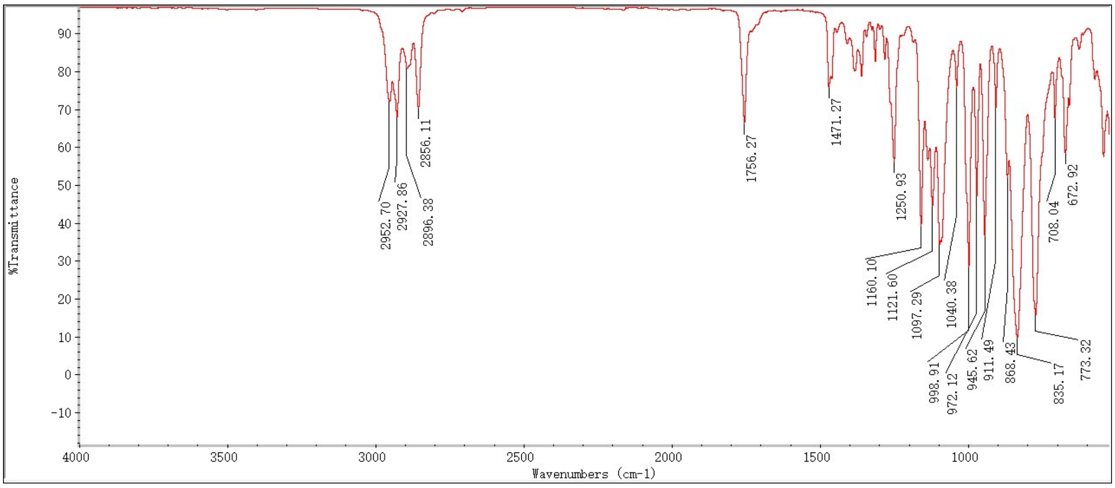

IR spectrum of compound **16**

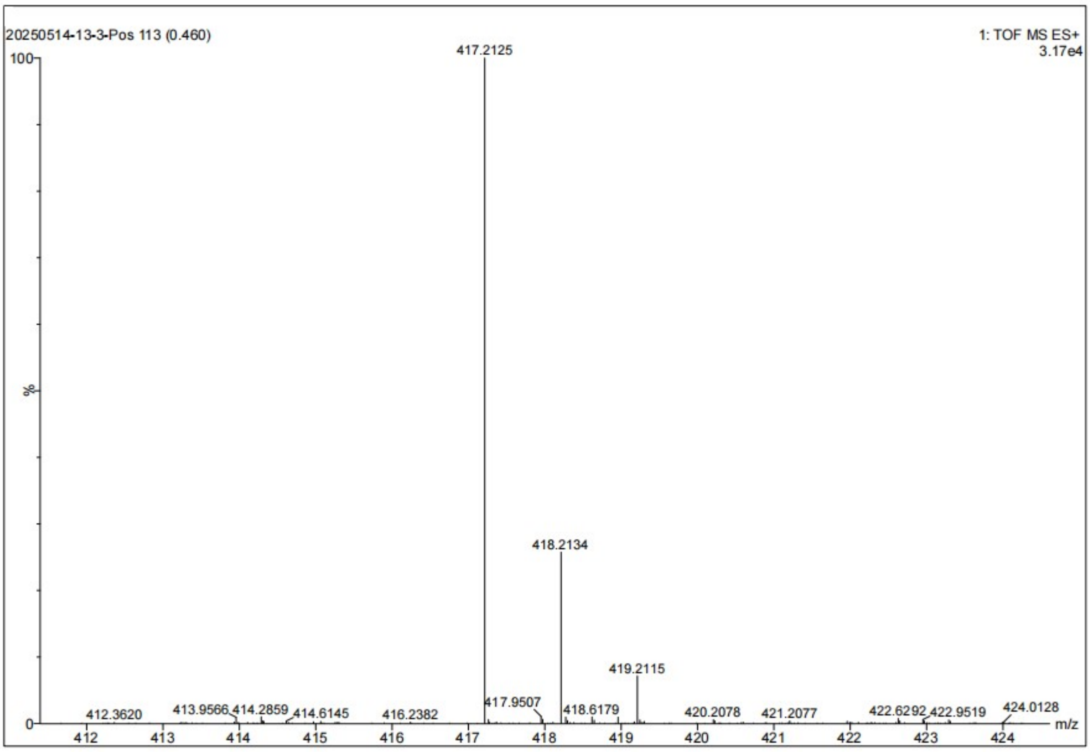

HRMS of compound **16**

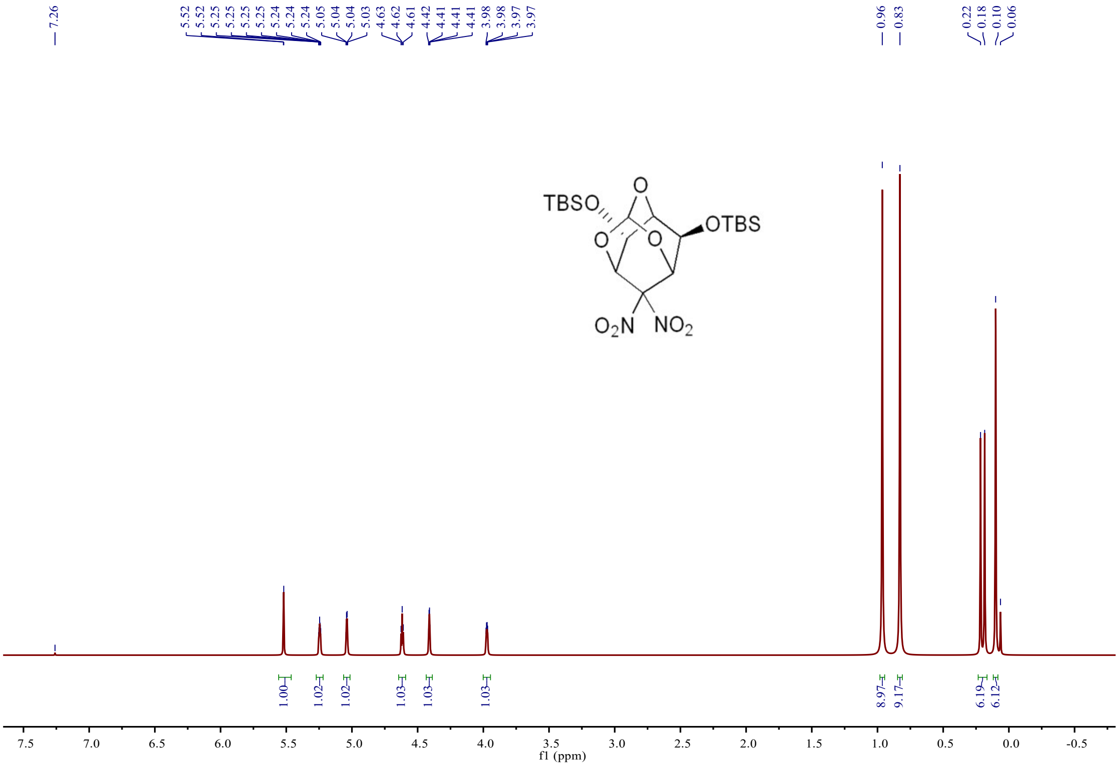

^{1}H NMR spectrum of compound **17** (Chloroform-*d*, 500 MHz)

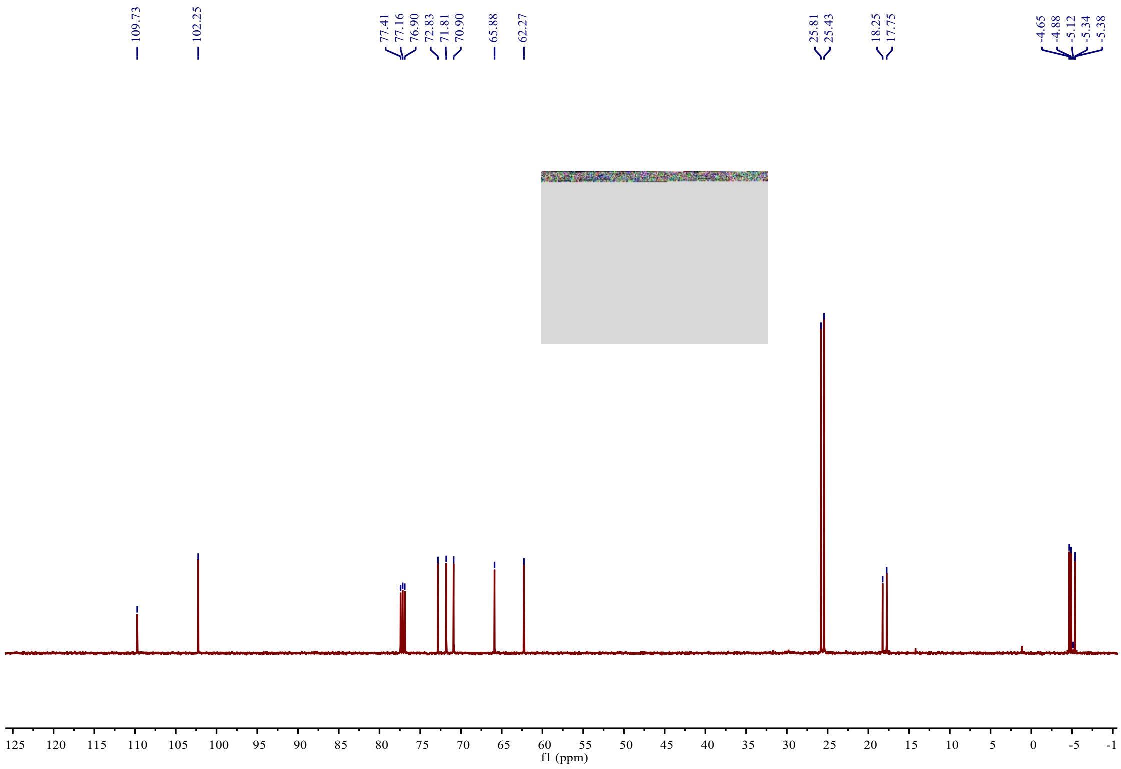

^{13}C NMR spectrum of compound **17** (Chloroform-*d*, 126 MHz)

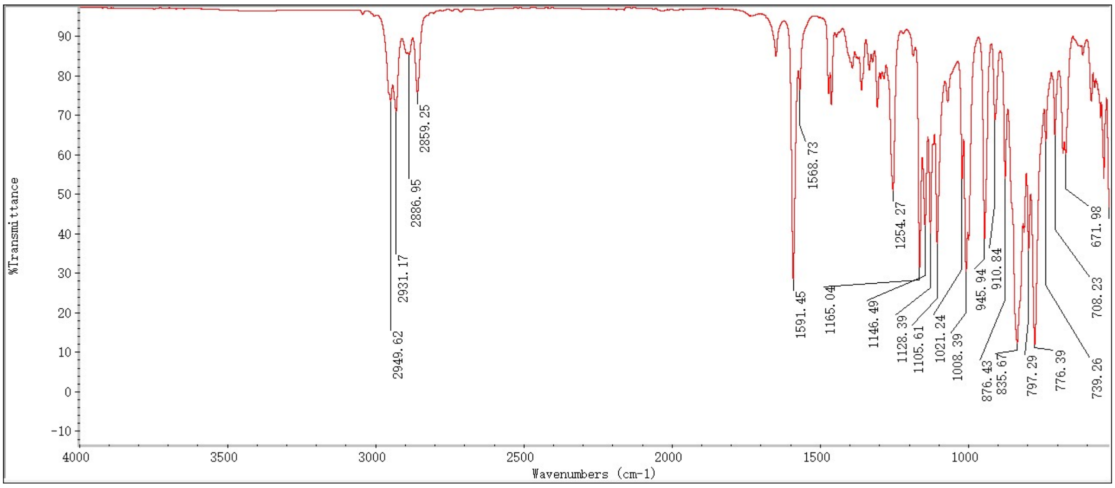

IR spectrum of compound **17**

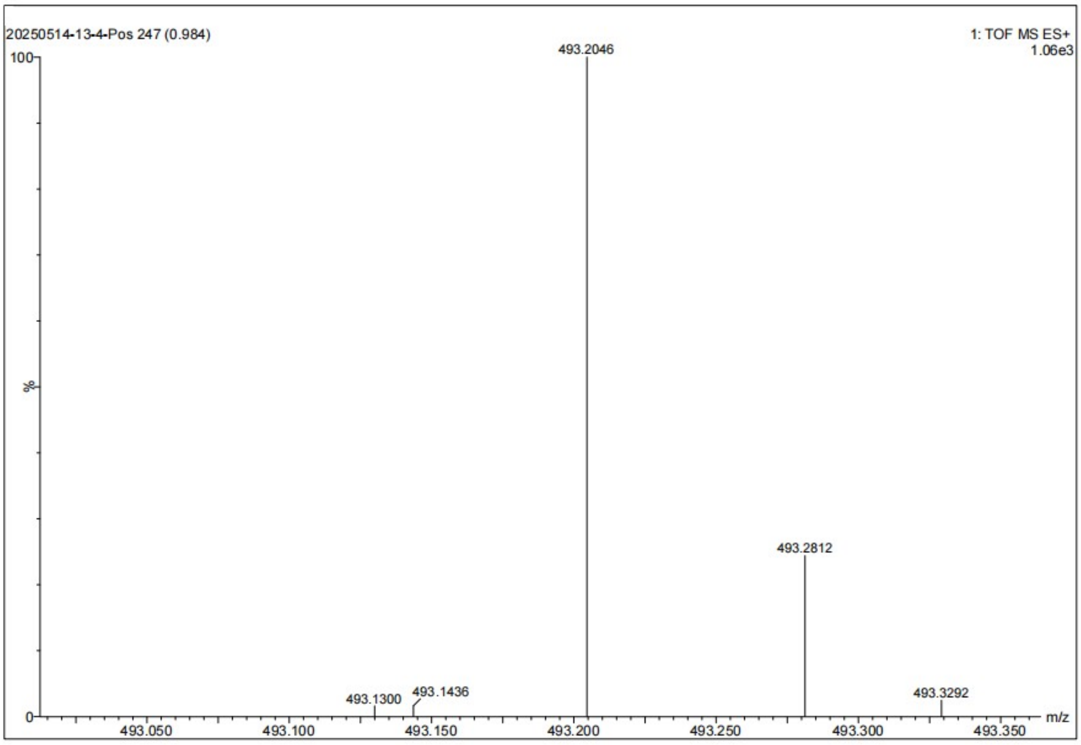

HRMS of compound **17**

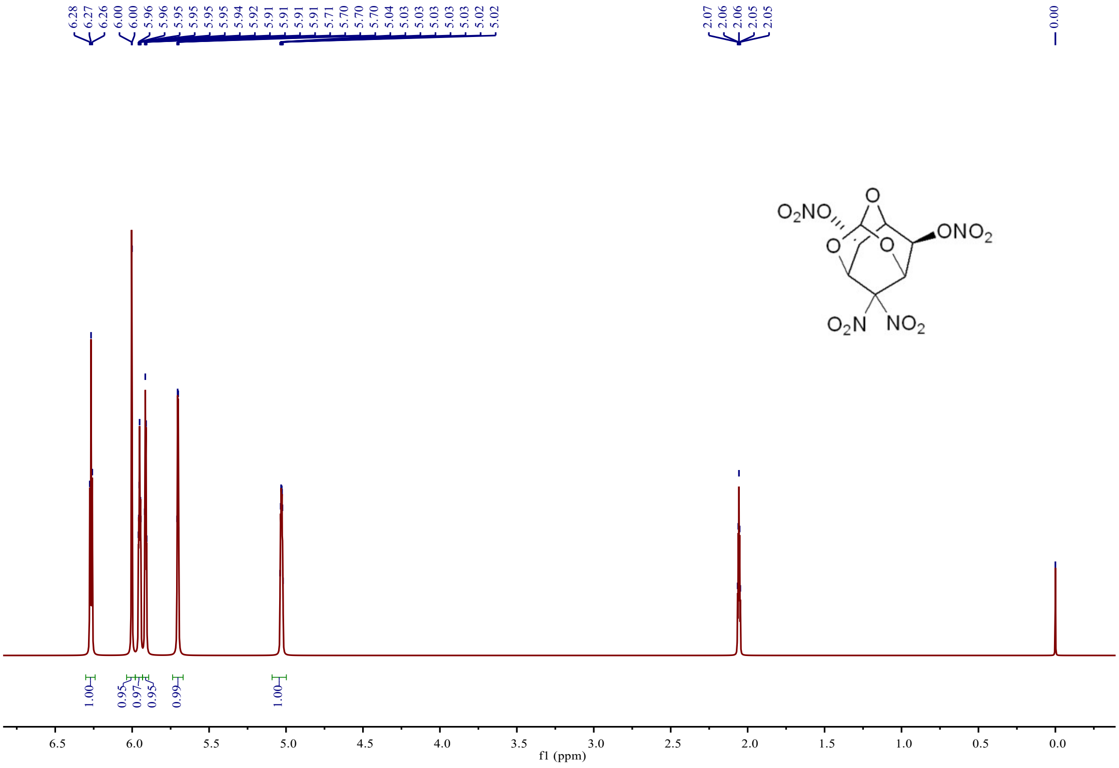

^{1}H NMR spectrum of compound **18** (Acetone-d_6, 500 MHz)

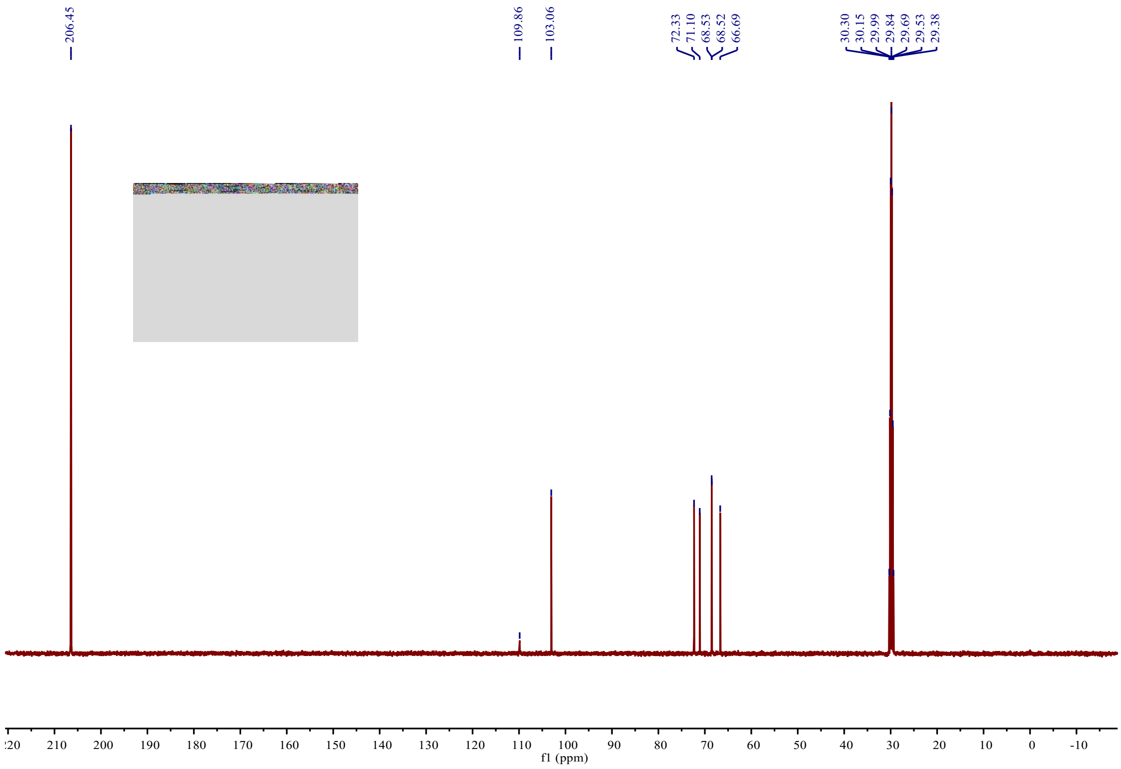

^{13}C NMR spectrum of compound **18** (Acetone-d_6, 126 MHz)

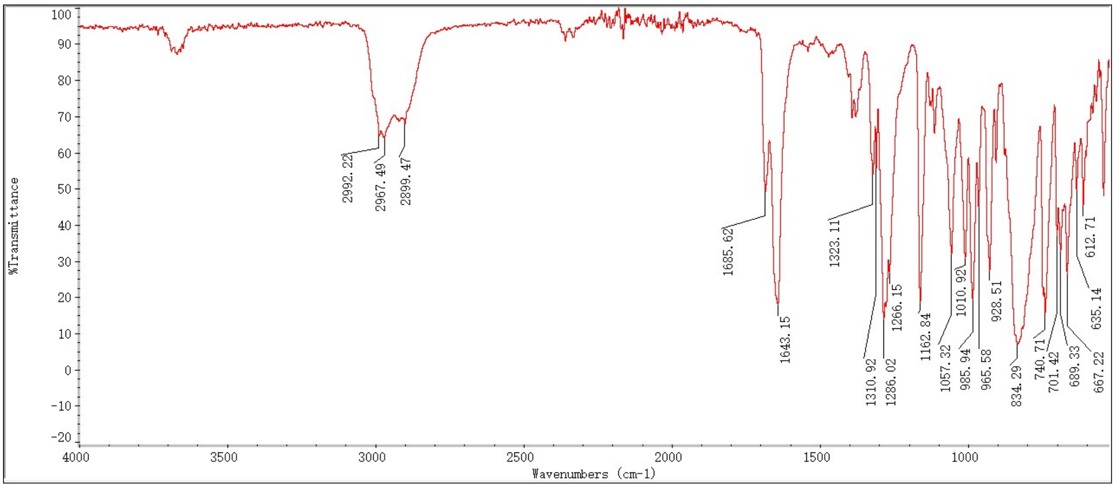

IR spectrum of compound **18**

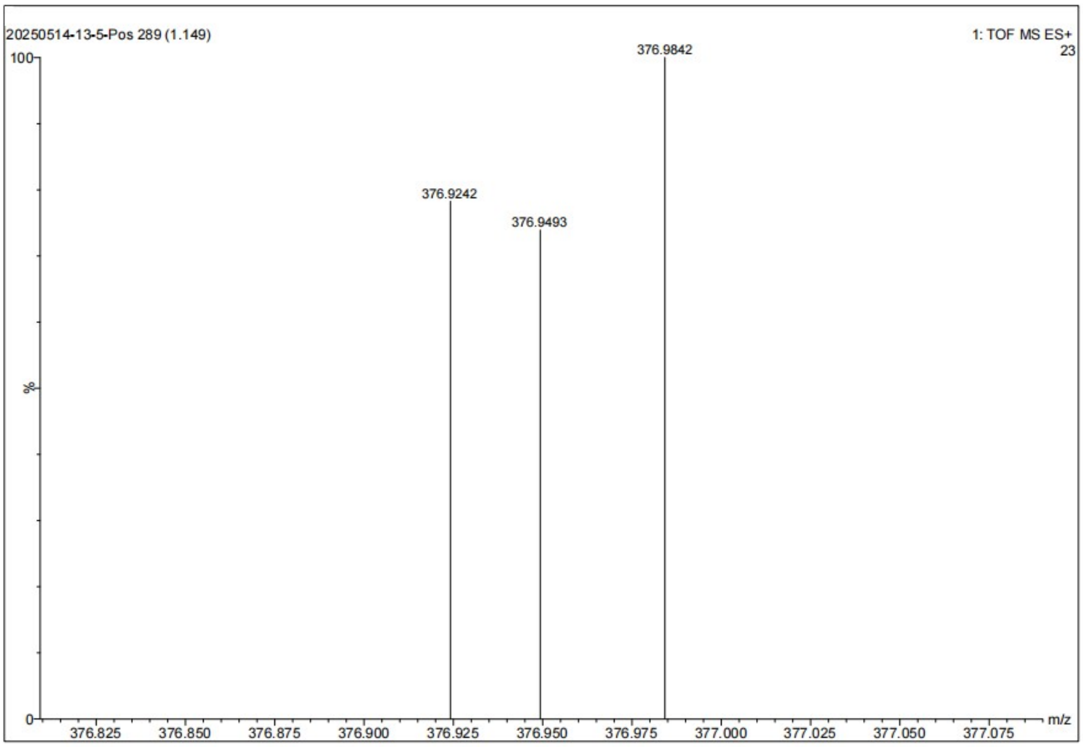

HRMS of compound **18**

5 The Heat of Formation

Because Q has much less effects than ρ on D and P, HOFs were estimated with the semi-empirical quantum chemical method PM3 from the atomization reactions, a default algorithm embedded in the software for of the semi-empirical method.

The HOF of the compound, for example, $C_7H_7N_3O_{12}$, at 298 K can be evaluated by the atomization reaction (1) and the formula (2).

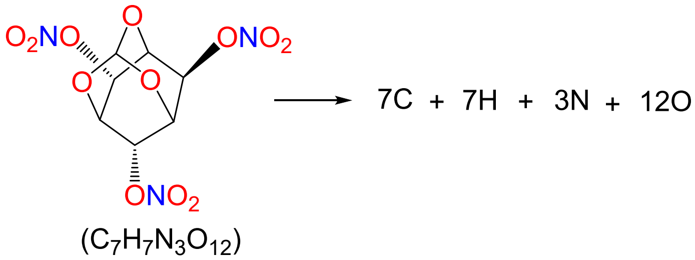

(1)

$$\Delta H_{298} = \sum H_P - \sum H_R = \sum HOF_P - \sum HOF_R \qquad (2)$$

where ΔH_{298} is enthalpy change. H_R and H_P are the enthalpies of reactant ($C_7H_7N_3O_{12}$) and products (C, H, N, and O) at 298 K respectively, and are calculated by PM3. HOF_R and HOF_P are the HOFs of the reactant and products at 298 K, respectively, and the experimental HOFs of C, H, O, and N are available. Therefore, the HOF of $C_7H_7N_3O_{12}$ can be evaluated.

6 TG-DSC curves of Energetic Compounds

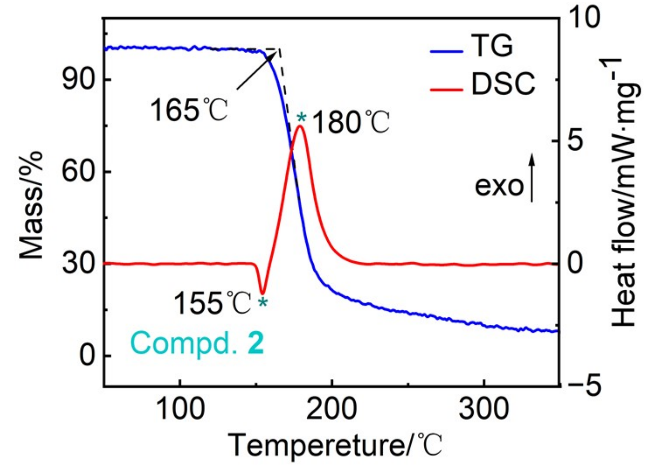

Figure S15. TG-DSC curves of compound **2**.

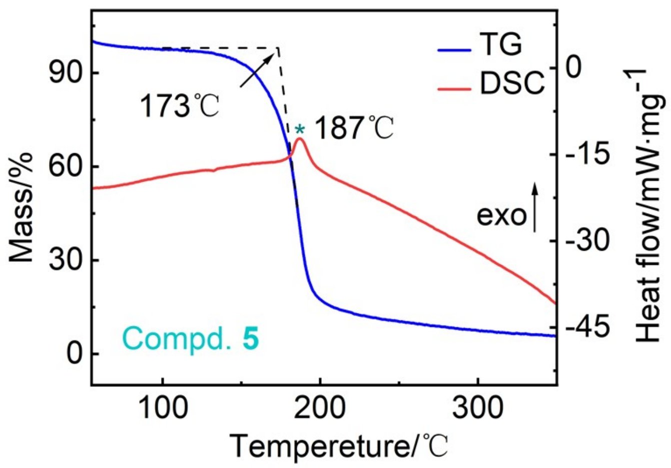

Figure S16. TG-DSC curves of compound **5**.

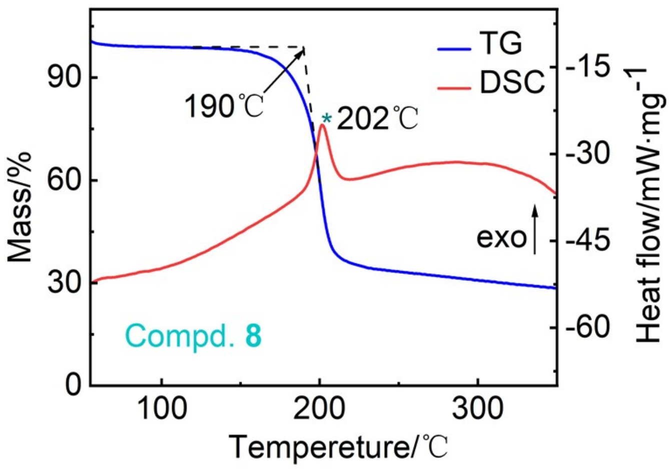

Figure S17. TG-DSC curves of compound **8**.

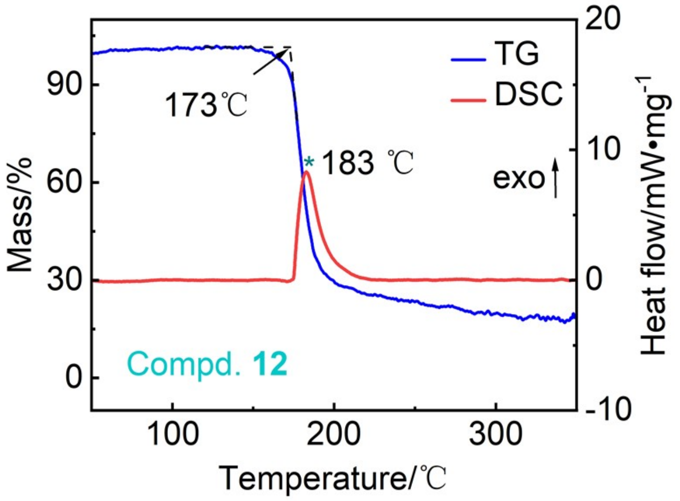

Figure S18. TG-DSC curves of compound **12**.

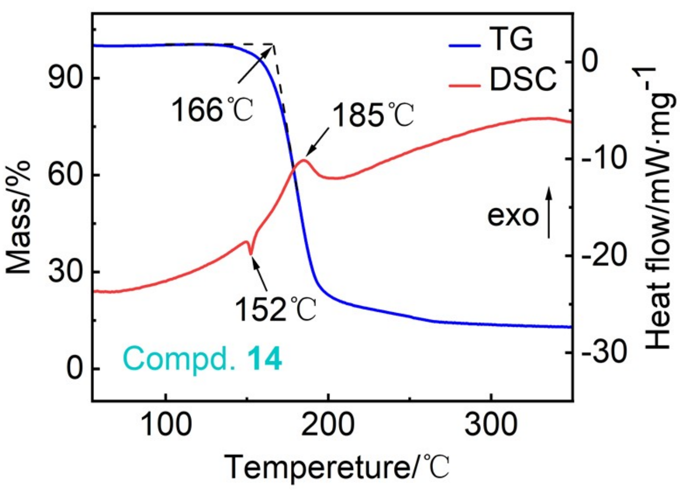

Figure S19. TG-DSC curves of compound **14**.

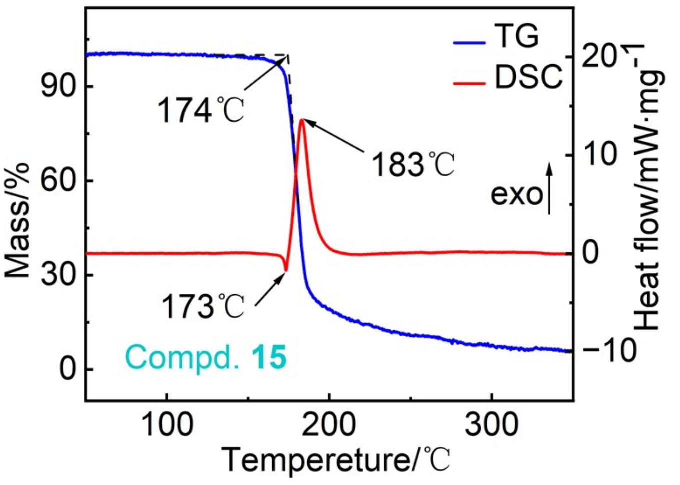

Figure S20. TG-DSC curves of compound **15**.

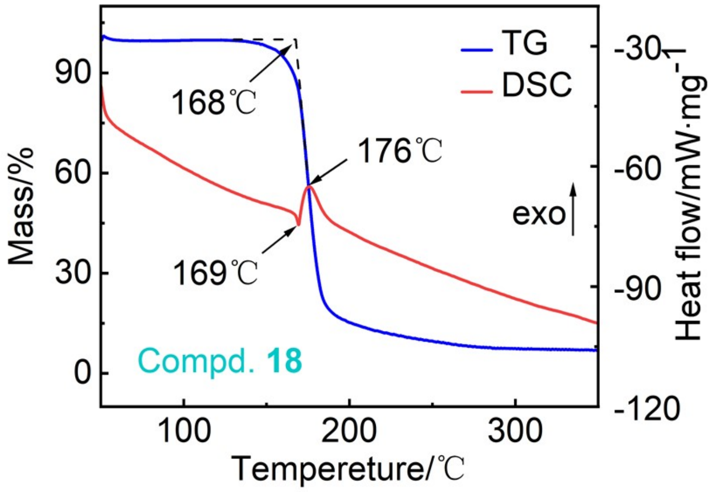

Figure S21. TG-DSC curves of compound **18**.
